# Phase-Separated Multienzyme Condensates for Efficient Synthesis of Imines from Carboxylic Acids with Enhanced Dual-Cofactor Recycling

**DOI:** 10.3390/ijms26104795

**Published:** 2025-05-16

**Authors:** Tingxiao Guo, Lifang Zeng, Jiaxu Liu, Xiaoyan Zhang, Yunpeng Bai

**Affiliations:** 1 State Key Laboratory of Bioreactor Engineering, Shanghai Collaborative Innovation Center for Biomanufacturing, East China University of Science and Technology, Shanghai 200237, China; 2 Shaanxi R&D Centre of Biomaterials and Fermentation Engineering, School of Chemical Engineering, Northwest University, Xi’an 710069, China

**Keywords:** multienzyme cascade, cofactor regeneration, intrinsically disordered protein, biocatalysis

## Abstract

Enzyme catalysis represents a promising approach for sustainable chemical synthesis, yet its industrial applications face limitations due to the inefficient regeneration and high cost of essential cofactors, such as adenosine-5′-triphosphate (ATP) and nicotinamide adenine dinucleotide phosphate (NADPH). While natural metabolic systems efficiently recycle cofactors through spatially organized enzymes, replicating this efficiency in vitro remains challenging. Here, we prepare a five-enzyme condensate system using liquid–liquid phase separation (LLPS) mediated by intrinsically disordered proteins (IDPs). By colocalizing a carboxylic acid reductase from *Norcadia iowensis* (*Ni*CAR) with a reductive aminase from *Aspergillus oryzae* (*Asp*RedAm) and three cofactor-regenerating enzymes, we generated a phase-separated catalytic condensate that enhanced ATP and NADPH recycling efficiency by 4.7-fold and 1.9-fold relative to free enzymes, respectively. Catalytic performance was correlated with the extent of phase separation, as confirmed by fluorescence microscopy, which revealed clear enrichment of ATP and NADPH within the condensates. This proximity effect enabled efficient cofactor turnover in the one-step reaction, achieving substrate conversion above 90% within 6 h and enhancing the space–time yield (STY) of the chiral imines 1.6-fold, with only one-fifth of the standard cofactor load. This approach creates a scalable and economic tool for performing multienzyme cascade reactions in vitro that are driven by the efficient recycling of multiple cofactors.

## 1. Introduction

Amines represent an important class of compounds in the chemical industry, widely used as intermediates in the synthesis of pharmaceuticals, agrochemicals, dyes, and materials. Their broad applicability continues to drive research efforts toward the development of more efficient and sustainable synthetic methodologies [1,2,3,4]. Traditional synthetic methods primarily relied on N-alkylation and reductive amination. N-alkylation, involving the reaction of amines with alkyl halides or sulfonates, often leads to over-alkylation and generates complex mixtures, complicating purification. Moreover, the toxicity and potential genotoxicity of alkylating agents present safety and environmental concerns [5,6,7]. Reductive amination, while more selective, typically requires stoichiometric reducing agents and excess amines, leading to side reactions and raising sustainability and cost issues [8,9].

In response to the limitations of traditional chemical methods, biocatalysis has emerged as a powerful and sustainable approach for amine synthesis, offering high regio- and stereoselectivity under mild conditions [10]. Enzymes such as transaminases [11,12,13], imine reductases (IREDs) [14,15,16], and reductive aminases (RedAms) [17,18] are widely applied in converting carbonyl compounds to chiral amines [19,20,21]. A particularly attractive and promising strategy is the combination of carboxylic acid reductases (CARs) with RedAms in cascade reactions, enabling the direct conversion of stable and renewable carboxylic acids into valuable chiral amines through intermediate aldehydes [22,23,24]. This approach not only avoids the need for hazardous chemical reductants, but also simplifies the process by reducing isolation steps, offering an atom-economical route from feedstocks to pharmaceutically relevant amines. Citoler et al. demonstrated a one-pot tandem cascade using CAR and ω-transaminase (ω-TA) for the amination of saturated and unsaturated fatty acids (C_6_ to C_18_), achieving conversions of up to 96% under mild conditions [22]. Similarly, Ramsden et al. developed a complementary one-pot system combining a RedAm with an alcohol oxidase (AO) for preparing primary alcohols, and a CAR with a RedAm for synthesizing carboxylic acids, achieving N-alkylation with high selectivity [23]. Despite these advancements, the dependence on costly cofactors, such as ATP and NADPH, together with their inefficient recycling under in vitro conditions, remains a major challenge for the practical application of these biocatalytic systems.

To overcome these limitations, inspiration can be drawn from the natural organization of biochemical reactions in living cells, such as liquid–liquid phase separation (LLPS) [25,26]. LLPS enriches biomolecules into concentrated droplets in aqueous phase, creating dynamic and membrane-free structures known as biomolecular condensates [27,28]. These condensates, unlike membrane-bound organelles, allow molecules to be rapidly exchanged between phases while concentrating specific biomolecules in the concentrated phase [29,30,31]. LLPS is primarily driven by multivalent weak interactions involving intrinsically disordered proteins (IDPs) or intrinsically disordered regions of proteins (IDRs), which lack stable secondary structures and are typically rich in polar and charged amino acids. These features allow IDPs to engage in multivalent, dynamic interactions that drive phase separation processes, which are essential for the formation and regulation of biomolecular condensates [32,33,34]. LLPS has recently emerged as a powerful strategy for enhancing intracellular biocatalysis and metabolic efficiency. By organizing enzymes into dynamic condensates, LLPS facilitates substrate channeling and increases local reactant concentrations. For example, Wan et al. explored the engineering of membraneless organelles using IDPs to enhance enzyme clustering, significantly improving the biosynthesis of 2′-fucosyllactose in *E. coli* 2.4-fold compared to traditional expression methods [35]. Wang et al. constructed synthetic multienzyme condensates in the prokaryote *E. coli* to enhance pathway efficiency, achieving a 5.7-fold increase in α-farnesene production [36].

In addition to LLPS-enhanced biosynthesis in vivo, recent studies have demonstrated that LLPS-based biocatalytic systems can accelerate enzymatic reactions in vitro [37,38,39]. For instance, Faltova et al. revealed that low-complexity domains can serve as molecular adhesives to induce the liquid–liquid phase separation of globular proteins, forming active microstructures with preserved enzymatic function and controlled protein release (diffusion coefficient ~1.1 × 10^−12^ m^2^/s) [38]. Liu et al. focused on constructing a phase-separated multienzyme system through high-affinity peptide interactions, demonstrating up to 70-fold enhancement in enzymatic catalytic efficiency when enzymes are compartmentalized within liquid condensates formed by postsynaptic density proteins [39]. Despite these advances, these LLPS systems predominantly focus on single enzymatic reactions and typically involve a single cofactor regeneration cycle. There remains a critical gap in the application of LLPS to more complex, multi-cofactor-driven enzymatic cascades, such as those required for amine synthesis involving both ATP- and NAD(P)H-dependent enzymes. Moreover, no reports to date have demonstrated the LLPS-mediated colocalization of more than three enzymes within a single condensate for cascade catalysis.

**Scheme 1:**
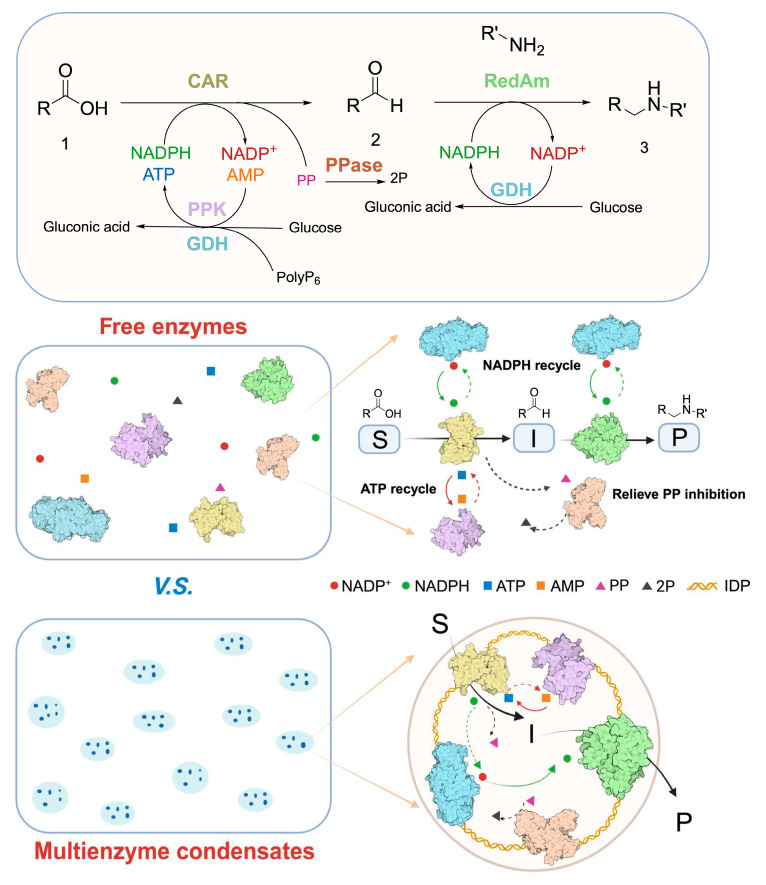
Diagram of the reactions in the free enzyme system and LLPS condensates. CAR = carboxylic acid reductase, PPK = polyphosphate kinase, GDH = glucose dehydrogenase, PPase = pyrophosphatase, RedAm = reductive aminase, PolyP_6_ = sodium hexametaphosphate, PP = pyrophosphate, P = ortho-phosphate, IDP = intrinsically disordered protein, S = carboxylic acid substrate, I = aldehyde intermediate, and P = amine product.

Herein, we present a modular strategy for the construction of multienzyme condensates using IDPs as scaffolds to achieve a spatially organized multienzyme catalytic system (Scheme 1). Our system integrates a carboxylic acid reductase from *Norcadia iowensis* (*Ni*CAR), a polyphosphate kinase from *Erysipelotrichaceae bacterium* (PPK12), a pyrophosphatase from *E. coli* (*Ec*PPase), and a glucose dehydrogenase from *Bacillus paranthracis* (*Bp*GDH), colocalized within condensates via IDP-mediated interactions [40,41,42]. *Ni*CAR drives the direct reduction of carboxylic acids to aldehydes without requiring substrate preactivation, which is exemplified by its high catalytic efficiency toward benzoic acid **1a**. PPK12 and *Bp*GDH can dynamically regenerate ATP and NADPH, respectively, enabling sustainable cofactor recycling. *Ec*PPase was used to prevent the inhibition caused by inorganic pyrophosphate (PP) from the reaction procedure. Following the construction and characterization of the four-enzyme LLPS catalytic system, the system was subsequently supplemented with *Asp*RedAm, an NADP(H)-dependent reductive aminase derived from *Aspergillus oryzae* [19,23]. This enzyme demonstrates catalytic versatility in mediating reductive coupling reactions between diverse carbonyl compounds and various primary/secondary amines. The integrated multienzyme system enables the direct one-step conversion of carboxylic acids to imines, thereby establishing an environmentally friendly biocatalytic platform for asymmetric amine synthesis.

## 2. Results and Discussion

### 2.1. Construction of IDP-Induced Self-Assembled Multienzyme Condensates

**Figure 1:**
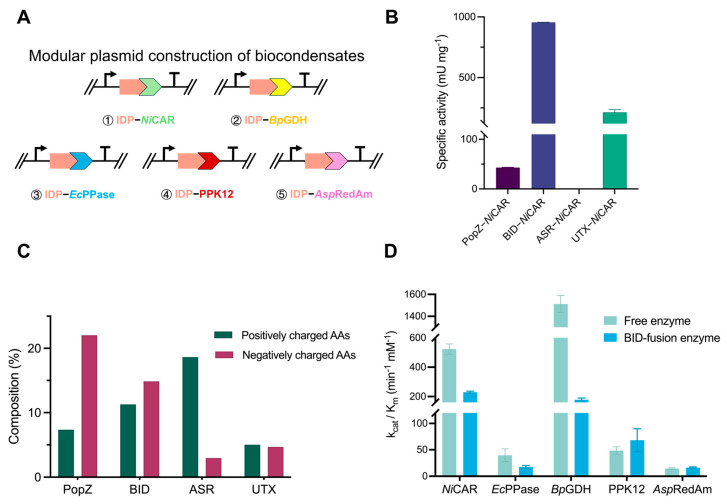
Construction and characterization of IDP-fused enzymes. (**A**) Plasmid construction of five IDP-fused enzymes. (**B**) Catalytic activities of four IDP-*Ni*CAR proteins. Assay conditions: 10 mM **1a**, 1 mM ATP, 0.2 mM NADPH, 10 mM MgCl_2_, 1 mM DTT, 100 mM MOPS pH 7.5, 30 °C. (**C**) Amino acid composition of four IDPs. (**D**) Catalytic activity of free enzymes and BID-fusion enzymes.

To induce enzyme colocalization via LLPS, we generated fusion proteins, which are single polypeptide chains created by genetically linking the coding sequence of an IDP scaffold to that of a target enzyme. The IDP serves as a phase-separation-inducing module to drive the self-assembly of enzymes into biomimetic condensates. To select the best IDP for efficient enzyme condensation without compromising catalytic activity, four different IDP–enzyme fusion proteins were systematically compared (Figure 1A and Figure S1). The panel included: UTX (also known as KDM6A), a histone H3K27 demethylase that functions as an important tumor suppressor; ASR, which acts as a chaperone to prevent protein aggregation and as an aggregate to promote it; polar organizing protein Z (PopZ) from *Caulobacter crescentus*, which assembles into microdomains at the poles of cells, playing a crucial role in regulating cell division by recruiting cell-cycle-regulating proteins; and BID, a proapoptotic member of the Bcl-2 family, which shares only the BH3 domain homology with other members of the family in its amino acid sequence and serves as an intracellular messenger [43,44,45,46,47,48]. Initially, all IDPs were fused to the N-terminus of *Ni*CAR, as the reduction reaction serves as the rate-limiting step. Moreover, prior studies have indicated that fusing IDPs to the C-terminus can significantly diminish enzymatic activity, likely due to the proximity of active sites to the C-terminal region. This reduction in activity may result from fewer accessible active sites in C-terminal fusion proteins [37]. Among the purified IDP-*Ni*CAR fusion proteins, BID-*Ni*CAR exhibited the highest catalytic activity (Figure 1B). The difference in the activity of the fusion proteins is suspected to be related to the different compositions of amino acid residues in the IDPs, resulting in different isoelectric points (PIs) of the IDPs (PopZ 4.02, BID 5.25, ASR 10.57, UTX 7.95) (Figure 1C). As the enzymatic reactions were conducted at pH 7.5, and CARs typically prefer mildly acidic conditions, the difference in PI and physiochemical properties likely created microenvironments that favored the relatively less negatively charged BID and the near-neutral UTX. In contrast, enzyme activities were substantially reduced when fused to ASR or PopZ, whose PIs are markedly low or high, respectively. Thus, BID was selected as the best IDP for subsequent multienzyme condensate construction, balancing catalytic activity and condensate stability. Although structural techniques, such as X-ray crystallography or NMR, were not employed in this study, we evaluated the structural integrity and functional preservation of the fusion proteins through enzymatic assays. All BID-fusion proteins exhibited activity comparable to that of their free-enzyme counterparts (Figure 1D and Table S2).

### 2.2. Characterization of Fluorescent Protein Localization with Enzyme Condensates

**Figure 2:**
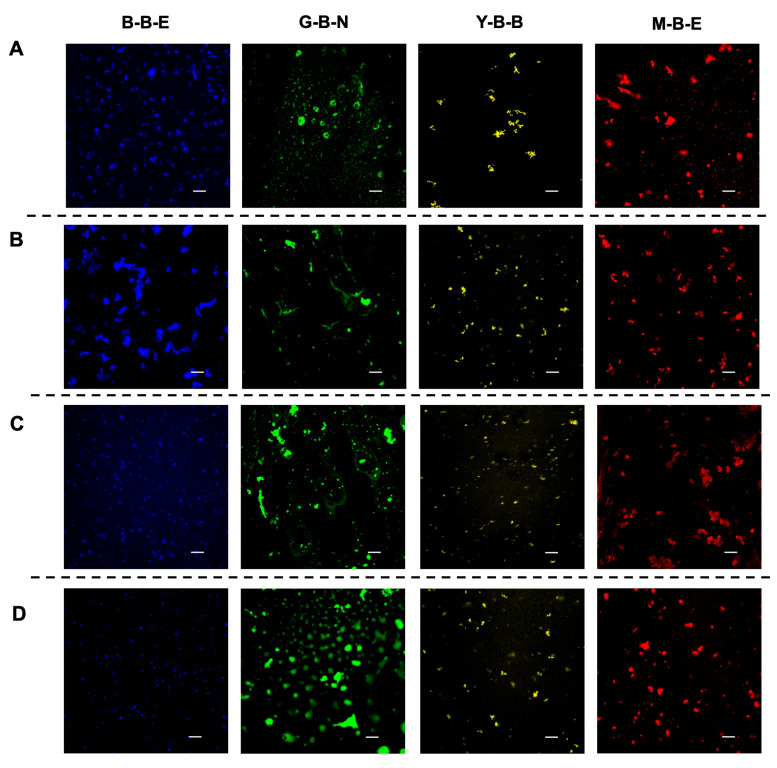
Confocal fluorescence images of fluorescent fusion proteins under identical conditions (10 μM protein, 30 min incubation, pH 7.5, 30 °C). Each panel was selected from a different experimental group in the supplementary figures, where a single variable was changed. Panel (**A**) was obtained from the 10 μM group in Figure S2 (protein concentration variation), (**B**) from the 30 min incubation group in Figure S3 (incubation time variation), (**C**) from the pH 7.5 group in Figure S4 (pH variation), and (**D**) from the group at 30 °C in Figure S5 (temperature variation). All panels represent the same set of conditions and were chosen to illustrate consistent phase behavior across independent experiments. Scale bar 10 μm.

To visualize phase separation, four fluorescently tagged fusion proteins were constructed: BFP-BID-*Ec*PPase (referred to as B-B-E), sfGFP-BID-*Ni*CAR (referred to as G-B-N), EYFP-BID-*Bp*GDH (referred to as Y-B-B), and mCherry-BID-PPK12 (referred to as M-B-P). The confocal fluorescence images confirmed that all four BID-fused enzymes could form condensates via liquid–liquid phase separation in vitro, and BID-linked enzymes demonstrated phase separation across a range of protein concentrations, pH levels, temperatures, and incubation times, as depicted in the phase diagram (Figure 2 and Figure S2–S5). The confocal fluorescent images demonstrated that all fusion proteins exhibited robust phase separation under a 10 μM protein concentration (Figure 2A), and as the concentration increased, the fluorescent condensates grew in size (Figure S2). Additionally, after 30 min incubation at 30 °C, all fluorescent proteins formed uniform condensates (Figure 2B), and well-dispersed droplets were consistently observed across all enzymes regardless of incubation time, further demonstrating the stability and durability of LLPS (Figure S3). All fluorescent-tagged fusion proteins underwent phase separation across a broad pH range (5.0–10.0), with the most stable phase separation at pH 7.5 (Figure 2C), indicating that the process is pH-tolerant (Figure S4). Under varying incubation temperatures, all proteins formed LLPS condensates, with the highest condensate formation observed at 30 °C (Figure 2D and Figure S5).

**Figure 3:**
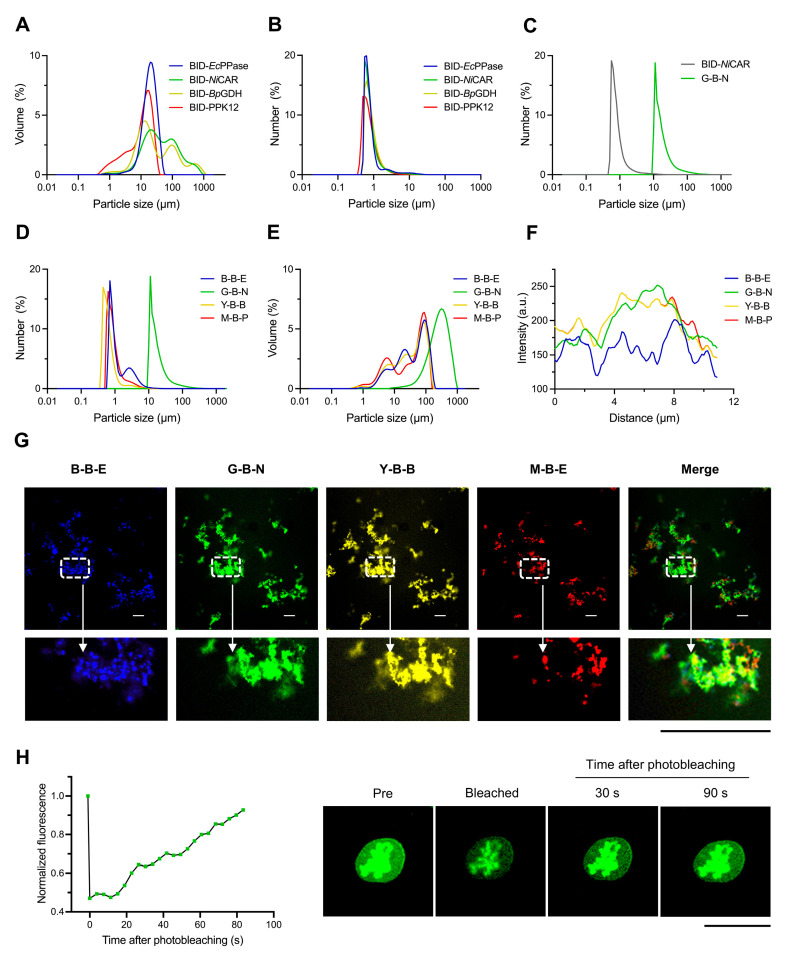
Characterization of free enzyme and phase-separated condensates. (**A**) Analysis of the particle size of four BID-fusion enzymes with the same concentration of 1 mg/mL. (**B**) Size distribution of condensates generated by four BID-fusion enzymes with the same concentration of 1 mg/mL. (**C**) Size distribution of condensates generated by 1 mg/mL BID-*Ni*CAR and 1 mg/mL sfGFP-BID-*Ni*CAR. (**D**) Analysis of the particle size of four fluorescent-tagged BID-fusion enzymes with the same concentration of 1 mg/mL. (**E**) Size distribution of condensates generated by four fluorescent-tagged BID-fusion enzymes with the same concentration of 1 mg/mL. (**F**) Fluorescence intensity analysis of the protein-rich phase (dashed-line box) of (**G**). (**G**) Confocal images of equal mixing of four fluorescent-tagged BID-fusion enzymes in different channels. Scale bar 10 μm. Each sample contained 5% PEG. (**H**) FRAP experiments using sfGFP-BID-*Ni*CAR. Scale bar 10 μm. Each sample contained 5% PEG.

The size distribution of the condensates at the same protein concentrations was determined using laser particle size analysis. For all the BID-fused proteins, as shown in Figure 3A, when performing volume-based analysis, BID-*Ni*CAR and BID-*Bp*GDH condensates exhibited diameters ranging from 1 μm to 1000 μm, while the other two condensates primarily formed smaller condensates with diameters ranging from 1 μm to 100 μm. The other, smaller, peak indicates that a fraction of condensates with smaller diameters was also formed (Figure S6A–D). In contrast, the number-based distributions indicated that most condensates were around 1 μm in diameter (Figure 3B). For all fluorescent-tagged fusion proteins, the results showed that diameters may change after fluorescent fusion (Figure 3C–G). The number distribution graph shows that G-B-N fusion proteins exhibited a marked size increase, shifting from a median diameter of 1 μm to >10 μm (Figure 3C). This observation suggests that fusion proteins with higher molecular weights tend to form larger condensates with a broader size distribution. In contrast, other fluorescently tagged constructs showed minimal changes in size distribution, underscoring the specificity of the molecular weight effects on condensate assembly (Figure S6E–G). These findings collectively indicate that fusion partner identity and molecular weight critically govern condensate dimensions, with implications for tailoring phase-separated systems in biocatalytic applications.

To demonstrate that all proteins can be recruited into the same condensate, equal quantities of purified B-B-E, G-B-N, Y-B-B, and M-B-P were mixed and incubated for 15 min before fluorescent measurement. Most of the condensates displayed the corresponding fluorescence in the same area, suggesting that they consisted of four proteins under the conditions tested (Figure 3F,G). As shown in Figure 3F, the fluorescence analysis of the area outlined by the dashed box in Figure 3G revealed that the four proteins exhibited overlapping positions with highly similar fluorescence intensities. This confirmed that all four proteins were indeed in the same condensate. Fluorescence recovery after photobleaching (FRAP) experiments revealed efficient fluorescence recovery within the condensates formed by the G-B-N fusion protein (Figure 3H), indicating the high internal mobility characteristic of liquid-like behavior in LLPS. This dynamic molecular exchange suggests that the BID-formed condensates possess the fluidity necessary to function as microreactors, enabling enzymatic reactions to occur efficiently within their interiors.

### 2.3. Aldehyde Production via a Phase-Separated NiCAR System

**Figure 4:**
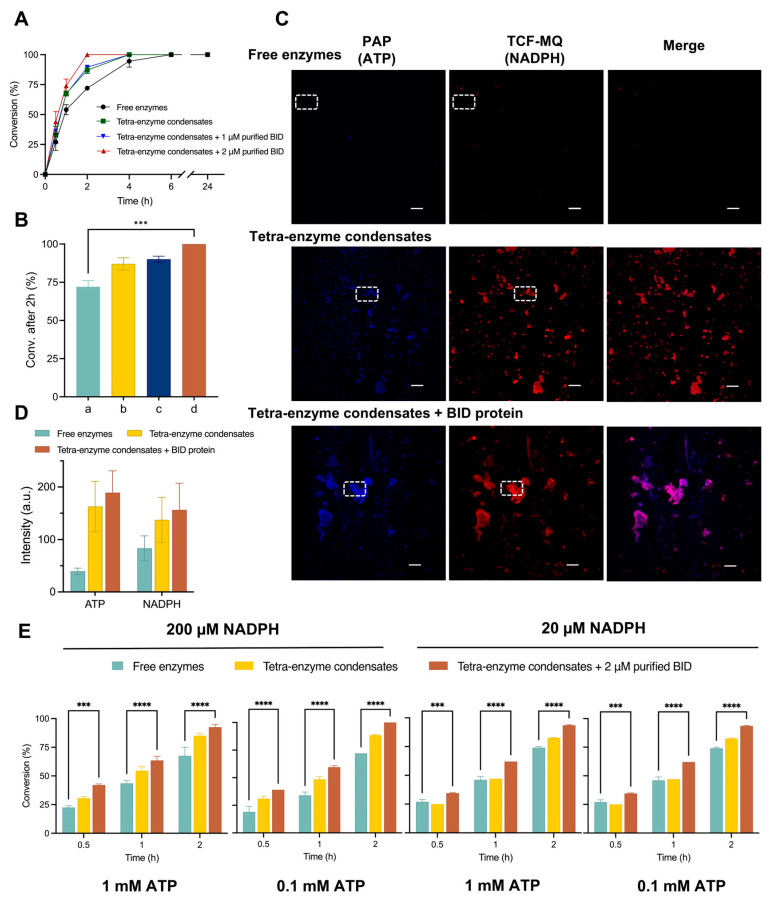
Reaction process of the free enzyme system and tetra-enzyme condensate systems. (**A**) Conversions of substrate **1a** in the free enzyme and tetra-enzyme condensates. Reaction conditions: substrate **1a** (15 mM), ATP (1 mM), NADPH (0.2 mM), MgCl_2_ (25 mM), PolyP_6_ (8 mM), β-d-glucose (100 mM), *Ni*CAR/BID-*Ni*CAR (0.2 U/mL), *Bp*GDH/BID-*Bp*GDH (0.2 U/mL), PPK12/BID-PPK12 (5 U/mL), *Ec*PPase/BID*-E*cPPase (5 U/mL), MOPS Buffer (100 mM, pH 7.5), at 30 °C. (**B**) Conversions of substrate **1a** in the free enzyme and tetra-enzyme condensates after 2 h. a: free enzymes; b: tetra-enzyme condensates; c: tetra-enzyme condensates with 1 µM purified BID; d: tetra-enzyme condensates with 2 µM purified BID. Data are presented as mean ± SD (n = 3). Statistical significance was determined by t-test. Asterisks indicate significant differences compared to the free enzyme system under identical conditions (*p* < 0.05; *** *p* < 0.001; **** *p* < 0.0001). (**C**) Distribution of ATP and NADPH in different reaction systems. The concentrations of PAP and TCF-MQ in the reaction system were 5 µM and 10 µM, respectively. Scale bar 10 μm. (**D**) Fluorescence analysis of the dashed-line box in (**C**). (**E**) The conversion of **1a** over time in various ATP and NADPH concentrations. Reaction conditions: substrate **1a** (15 mM), ATP (1 or 0.1 mM), NADPH (20 or 200 µM), MgCl_2_ (25 mM), PolyP_6_ (8 mM), β-_D_-glucose (100 mM), *Ni*CAR/BID-*Ni*CAR (0.2 U/mL), *Bp*GDH/BID-*Bp*GDH (0.2 U/mL), PPK12/BID-PPK12 (5 U/mL), *Ec*PPase/BID-*Ec*PPase (5 U/mL), 2 µM BID protein, MOPS buffer (100 mM, pH 7.5), at 30 °C. Data are presented as mean ± SD from three independent experiments (n = 3). Statistical significance was determined by two-way ANOVA followed by Sidak’s multiple comparisons test. Asterisks indicate significant differences compared to the free enzyme system at the corresponding time point (*** *p* < 0.001; **** *p* < 0.0001).

The application of CARs is constrained by their reliance on expensive cofactors [42,49]. Moreover, the reaction byproducts, such as pyrophosphate, AMP, and NADP^+^ are known to act as inhibitors [50]. Herein, we utilized an established cell-free regeneration system to recycle both ATP and NADPH, thereby driving aldehyde production in both the free enzyme system and the LLPS multienzyme condensate system. To evaluate the effect of condensate-mediated enrichment on enzymatic reactions, we first compared the substrate conversion efficiency of two systems—the free enzyme system (*Ni*CAR, *Bp*GDH, PPK12, and *Ec*PPase) and the tetra-enzyme condensate system (BID-*Ni*CAR, BID-*Bp*GDH, BID-PPK12, and BID-*Ec*PPase)—under identical reaction conditions using substrate **1a**. The results in Figure 4A show that at all measured time points, the condensate system exhibited higher substrate conversion than the free enzyme system. Specifically, in the free enzyme system, the conversions at 1 h and 2 h were 54.0% and 72.1%, respectively. In contrast, the tetra-enzyme condensate system achieved conversions of 67.5% and 87.0% at equivalent enzymatic activity (Figure 4B).

Previous studies have suggested that the addition of purified IDP promotes protein condensation [37]. To further investigate the role of BID, we supplemented the tetra-enzyme condensate system with 1 μM and 2 μM of purified BID. The results indicated that exogenous BID markedly enhanced the reaction rate: the tetra-enzyme condensate system with 1 μM and 2 μM BID exhibited higher conversion than the previously mentioned two systems at all time points, with >99% conversion of substrate **1a** within 2 h when BID addition was 2 μM (Figure 4A,B). This effect is likely attributable to a proximity enhancement, whereby the high concentration of BID brings enzymes and substrates into closer spatial confinement. The reduced diffusion distance facilitates cofactor accumulation within the condensates, thereby accelerating cofactor recycling and overall catalytic efficiency compared to the free enzyme system. To further elucidate the potential proximity effect conferred by enzyme condensates, kinetic analyses were conducted on the free enzyme system and the tetra-enzyme condensate system (Table S2, entry 11 and 12). Specifically, the tetra-enzyme condensate system significantly reduced the *K*_m_ from 3.41 ± 0.12 mM (for the free enzyme system, entry 11) to 0.48 ± 0.03 mM, and enhanced the catalytic efficiency (*k*_cat_*/K*_m_) to 594.69 ± 38.62 min^−1^ mM^−1^ (entry 12), accompanied by a 2.1-fold enhancement in catalytic efficiency, aligning with previous studies on dual-enzyme condensates [37]. The marked decrease in *K*_m_ suggests enhanced substrate affinity, which is likely attributable to local substrate enrichment within the confined microenvironment of the condensates. These findings suggest that the accelerated reaction rate observed is primarily due to a reduction in the apparent *K*_m_, facilitating the tetra-enzyme condensate system to achieve maximum activity in a shorter time.

The type of buffer can influence enzyme activity, phase behavior, and cofactor stability, while the concentration of PolyP_6_ is important for ATP regeneration via PPK12. We next evaluated the catalytic performance of the free enzyme system and tetra-enzyme condensate systems under different buffer systems and PolyP_6_ concentrations (Figures S7 and S8). According to Figure S7, the tetra-enzyme condensate systems consistently outperformed the free enzyme system across all tested buffers. The BID-enhanced tetra-enzyme condensates achieved >99% conversion within 2 h in MOPS buffer, exceeding the performance observed in HEPES (97%) and Tris-HCl (88%) under identical conditions. Similarly, tetra-enzyme condensate systems consistently outperformed the free enzyme system when tested under various PolyP_6_ concentrations (Figure S8). Incomplete conversions were observed at 2 mM and 4 mM PolyP_6_ across all systems tested, with tetra-enzyme condensates with BID addition achieving 54.5% conversion and the free enzyme system only 27.5%. At 8 mM PolyP_6_, the tetra-condensate system with 2 μM purified BID achieved full conversion within 2 h. Further increasing the concentration to 20 mM had a limited impact on conversion. Thus, 8 mM PolyP_6_ offers a balance between reaction efficiency and economic feasibility. These results suggest that the incorporation of IDPs facilitates the assembly of enzyme-rich condensates, which are associated with improved catalytic performance and tunable reaction kinetics.

### 2.4. Impact of ATP and NADPH Recycling on the Conversion of Benzoic Acid **1a**

To investigate the distribution and recycling efficiency of ATP and NADPH during the reaction, fluorescent probes TCF-MQ (for NADPH) and PAP (for ATP) were applied in the LLPS system. TCF-MQ is a dicyanomethylene-based small-molecule fluorophore, while PAP is a pyrene-based pincer sensor that binds phosphate anions with high affinity [51,52]. By labeling NADPH and ATP using TCF-MQ and PAP, respectively, during the reaction, we found that NADPH and ATP were enriched in tetra-enzyme condensates during catalytic reactions, and the condensates with the addition of purified BID exhibited a greater local concentration elevation of NADPH and ATP inside these condensates, with the formation of larger-sized condensates (Figure 4C). Fluorescence analysis in the dashed-line boxes reveals that the concentration of ATP and NADPH in the LLPS reaction system is approximately 4.7-fold and 1.9-fold higher than in the free enzyme reaction system, respectively (Figure 4D). These findings validate our hypothesis that enzyme condensation, driven by spatial proximity effects, significantly enhances cofactor enrichment within biomolecular condensates. Moreover, the degree of condensation is positively correlated with the overall reaction rate.

To evaluate cofactor recycling within LLPS condensates, the conversion of benzoic acid **1a** by the free enzyme and tetra-enzyme condensates was measured under various ATP and NADPH concentrations. As shown in Figure 4E, a comparison among the three systems revealed that the two condensate-based systems consistently exhibited higher substrate conversion rates than the free enzyme system across all time points, with the differences being particularly pronounced within the first two hours. Under the conditions of 200 μM NADPH, the tetra-enzyme condensate system with BID addition exhibited better catalytic performance across different ATP concentrations. At 1 mM ATP, full substrate conversion was achieved within 2 h, compared to only 72.1% using the free enzyme system. Even when the ATP concentration was reduced to 0.1 mM, the condensate system with BID addition maintained high conversion (94%), whereas the free enzyme system was merely 78%. Under conditions with limited NADPH (20 μM), the performance gap remained substantial. At 1 mM ATP, the BID-added tetra-enzyme condensate achieved 95% conversion within 2 h, while the free enzyme system reached only 60%. Reducing ATP to 0.1 mM under the same NADPH level resulted in 93% conversion by the condensate system, compared to 73% by the free enzyme system. Notably, even at reduced levels (2 μM NADPH, 1mM/0.1 mM ATP), the tetra-enzyme condensate systems still achieved higher conversion than the free enzyme counterpart (Figure S9). These results confirm that the condensate architecture enables efficient catalysis under cofactor-limited conditions, likely due to improved cofactor recycling and local substrate availability. Differences in conversions represent differences in ATP and NADPH recycling efficiency (substrate converted per cofactor per unit time), with an up to 1.9-fold increase in the condensates compared to free enzymes within 0.5 h (1 mM ATP and 20 μM NADPH). These findings suggest that condensate-based systems can improve cofactor recycling efficiency, particularly for NADPH and ATP. In the free enzyme system, cofactor recycling relies on the random diffusion of NADPH and ATP among dispersed enzymes, leading to inefficiencies at low concentrations. In contrast, the LLPS tetra-enzyme condensates confine cofactors within a dense local environment, facilitating rapid exchange and enabling efficient catalysis even under limited cofactor conditions. This enhanced catalytic efficiency is attributed to the proximity effect, where BID-fused enzymes self-assemble into dynamic condensates that promote rapid cofactor exchange and substrate processing. Compared to the free enzyme system, the tetra-enzyme condensates sustain higher reaction rates under low cofactor concentrations, highlighting the role of phase separation in improving local cofactor availability and recycling. This confined organization enhances molecular proximity among enzymes, substrates, and cofactors, thereby promoting catalytic synergy and improving overall reaction rates. Comprehensive enzymatic characterization revealed that BID-fused enzymes retained properties similar to those of their free forms in terms of pH, temperature, and solvent tolerance, with only minor changes in thermostability (Figure S10–S12). Overall, IDP fusion had a limited impact, and the enzymes remained well suited for subsequent applications.

### 2.5. Substrate Scope and Scale-Up Amine Production

**Figure 5:**
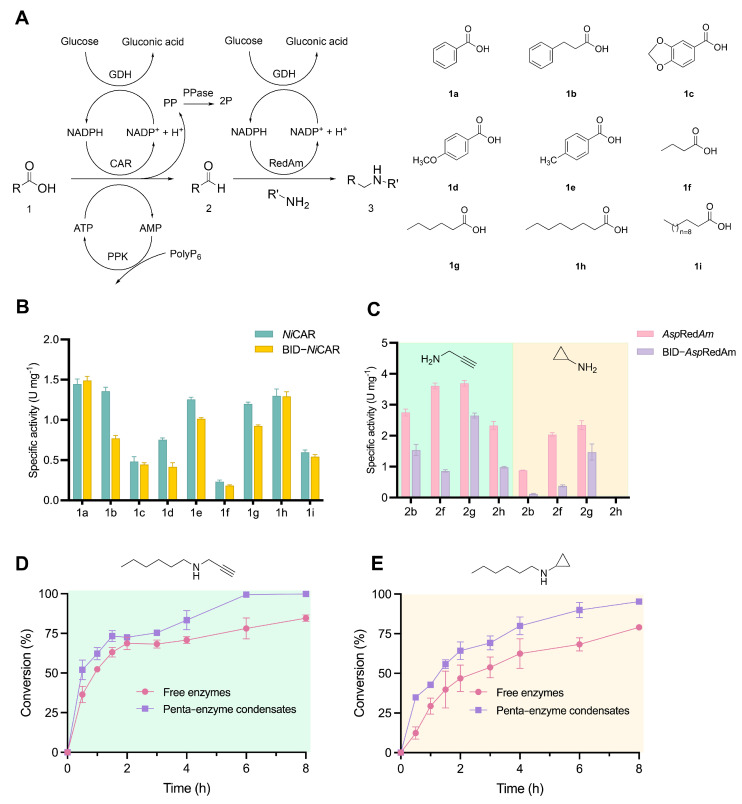
Substrate scope of CARs, RedAms, and enzyme-catalyzed reactions. (**A**) Reaction scheme for the in vitro production of amines and substrates selected for CAR substrate scope analysis. PP: pyrophosphate, P: *ortho*-phosphate. (**B**) Specific activity of *Ni*CAR and BID-*Ni*CAR toward selected substrates. Reaction condition: MOPS buffer (100 mM, pH 7.5), MgCl_2_ (10 mM), DTT (1 mM), ATP (1 mM), NADPH (0.2 mM), substrate (10 mM), at 30 °C. (**C**) Specific activity of *Asp*RedAm and BID-*Asp*RedAm toward selected aldehydes and amine donors. Reaction condition: MOPS buffer (100 mM, pH 7.5), aldehyde (10 mM), amine (20 mM), NADPH (0.2 mM), at 30 °C. (**D**) Time courses of reduction of substrate **1g** and propargylamine catalyzed by free enzymes and penta-enzyme condensates. Reaction condition: substrate **1g** (10 mM), propargylamine (20 mM), ATP (0.2 mM), NADPH (0.04 mM), MOPS buffer (100 mM, pH 7.5), MgCl_2_ (25 mM), β-_D_-glucose (100 mM), PolyP_6_ (8 mM), *Ni*CAR/BID-*Ni*CAR (1 U/mL), *Asp*RedAm/BID-*Asp*RedAm (1 U/mL), PPK12/BID-PPK12 (5 U/mL), *Ec*PPase/BID-*Ec*PPase (5 U/mL), *Bp*GDH/BID-*Bp*GDH (1 U/mL), and BID protein (2 μM). (**E**) Time courses of reduction of substrate **1g** and cyclopropylamine catalyzed by free enzymes and penta-enzyme condensates. Reaction condition: substrate **1g** (10 mM), cyclopropylamine (20 mM), ATP (0.2 mM), NADPH (0.04 mM), MOPS buffer (100 mM, pH 7.5), MgCl_2_ (25 mM), β-d-glucose (100 mM), PolyP_6_ (8 mM), *Ni*CAR/BID-*Ni*CAR (1.25 U/mL), *Asp*RedAm/BID-*Asp*RedAm (1.25 U/mL), PPK12/BID-PPK12 (5 U/mL), *Ec*PPase/BID-*Ec*PPase (5 U/mL), *Bp*GDH/BID-*Bp*GDH (1.25 U/mL), and BID protein (2 μM).

Assembling both cofactor regeneration reactions and the carboxylate reduction in vitro, the system was first tested with several carboxylic acid substrates (Figure 5A). Both *Ni*CAR and BID-*Ni*CAR showed activity toward all selected substrates (Figure 5B and Figure S13), efficiently reducing them to their corresponding aldehydes. Kinetic analysis revealed that BID-*Ni*CAR exhibits catalytic efficiency (*k*_cat_/*K*_m_) similar to that of its free enzyme counterpart across all tested substrates, indicating preserved enzymatic functionality and structural stability (Table S3).

To enable direct one-pot amination from carboxylic acids, *Asp*RedAm and its BID-fused variant (BID-*Asp*RedAm) were integrated into the tetra-enzyme system, forming a penta-enzyme cascade. The substrate scope of the free *Asp*RedAm and the fusion BID-*Asp*RedAm was evaluated against a panel of selected aldehydes and two amine donors. As shown in Figure 5C, both enzymes exhibited catalytic activity toward all tested substrates, except for substrate **2h** with cyclopropylamine. Among the aldehydes, aldehyde **2g** consistently displayed the highest activity with both amine donors.

The catalytic performance of the penta-enzyme condensate system was assessed under cofactor-limiting conditions (0.2 mM ATP and 0.04 mM NADPH) using carboxylic acid substrate **1g** and two different amine donors. With propargylamine as the amine donor, the penta-enzyme condensate system achieved >99% conversion within 6 h, whereas the free enzyme system reached only 77.3% conversion under the same conditions (Figure 5D). When cyclopropylamine was used as the amine donor, the condensate system consistently outperformed the free enzyme system, reaching 94.5% conversion within 8 h compared to 77.9% for the free enzyme system under the same conditions (Figure 5E). This enhanced efficiency suggests that condensate-based systems may offer greater productivity in industrial applications by enabling higher product yields within a shorter time. To evaluate the scalability and industrial potential of the penta-enzyme system, scale-up reactions were conducted under reduced cofactor concentrations for amine production (Table 1). With 10 mM **1g**, 20 mM propargylamine, 0.2 mM ATP, and 0.04 mM NADPH in the 5 mL reaction system, the free enzyme system achieved 83% substrate conversion after 8 h (Table 1, entry 1) with an STY of 144 mg L^−1^ h^−1^. In contrast, the penta-enzyme condensate system under the same conditions achieved a conversion of >99% after 6 h (Table 1, entry 2) and an STY of 232 mg L^−1^ h^−1^; when cyclopropylamine was selected as the amine donor, the free enzyme system achieved 79% conversion after 8 h (Table 1, entry 3) with an STY of 132 mg L^−1^ h^−1^, while the penta-enzyme system reached 90% conversion after 6 h (Table 1, entry 4) with an STY of 212 mg L^−1^ h^−1^. In all cases, the STY of the LLPS penta-enzyme system was 1.6-fold higher than that of the free enzyme system. These findings further suggest that the penta-enzyme condensate accelerated the reaction rate through a proximity effect, offering a promising strategy for large-scale biocatalytic synthesis. Even when the cofactor input was reduced to one-fifth of that used in conventional reaction setups, the penta-enzyme system efficiently converted substrates with 1.6-fold higher STY than that of the free enzyme system, providing an effective solution to reduce cofactor costs in industrial amine production. Compared to traditional free enzyme systems, penta-enzyme condensates offer benefits such as reduced mass transfer limitations and improved microenvironment control, making them well suited for scalable biocatalytic applications. Although our scale-up reactions demonstrated enhanced catalytic performance, industrial implementation may require further optimization, such as continuous-flow reactors or enzyme immobilization strategies, to stabilize condensates during prolonged or large-scale operations.

## 3. Materials and Methods

### 3.1. General Information

ASR, BID, and UTX strains were retained from the laboratory, and PopZ, *Ni*CAR, PPK12, *Ec*PPase, *Bp*GDH, and *Asp*RedAm were synthesized by Beijing Tsingke Biotech Co., Ltd. (Beijing, China). DNA primers were also synthesized from Beijing Tsingke Biotech Co., Ltd. (Beijing, China). All plasmid vectors, restriction enzymes, and ligases were purchased from Takara Bio (Beijing, China). DNA gel extraction kits and plasmid extraction kits were purchased from Yeasen Biotech (Shanghai, China). The reagents for bacterial culture were purchased from Oxide and ThermoFisher (Shanghai, China). All chemicals were purchased from Titan Chemical (Shanghai, China) and Aladdin (Shanghai, China) and used without further treatment unless otherwise indicated. NADPH and NADP^+^ were purchased from Bontac Bioengineering (Shenzhen, China). HPLC analysis was performed on a Shimadzu LC-2010A HT apparatus equipped with an Agilent XDB-C18 column (5.0 μm, 4.6 × 250 mm). Fluorescence images of condensate formation and cofactor recycling in a microreactor were taken using a Nikon A1R^+^ laser scanning confocal microscope (Nikon Instruments, Tokyo, Japan). All experiments were performed in triplicate unless otherwise stated. Data are presented as mean ± standard deviation (SD).

### 3.2. Preparation of Fusion Proteins

All proteins used in this study were codon-optimized for *Escherichia coli* expression. To induce enzyme colocalization via LLPS, we generated fusion proteins, which are single polypeptide chains created by genetically linking the coding sequence of an IDP scaffold to the coding sequence of a target enzyme. Sequences for all fusion proteins are provided in the Supplementary Materials. Genes encoding for PopZ-*Ni*CAR, ASR-*Ni*CAR, BID-*Ni*CAR, UTX-*Ni*CAR, and sfGFP-BID-*Ni*CAR were cloned into the pRSFDuet-1 vector, while BID-*Ec*PPase, BID-PPK12, BID-*Bp*GDH, BID-*Asp*RedAm, and all the other fluorescent tag genes (BFP, sfGFP, EYFP, mCherry) were cloned into a pET-28a(+) vector. Target genes were amplified via polymerase chain reaction (PCR) using PrimeSTAR Max DNA Polymerase (Takara Bio, Beijing, China) and the primer pairs listed in Table S1. Amplified DNA fragments were purified by agarose gel electrophoresis, digested with *EcoR*I and *Hind*III (Takara Bio), and ligated into the linearized pET-28a(+) vector using a 2×Hieff Clone^®^ One Step Cloning Kit (Yeasen Biotech, Shanghai, China), following the manufacturer’s protocols (all fusion constructs were generated analogously). Transformed colonies were screened using colony PCR, and positive clones were verified through Sanger sequencing. Validated recombinant plasmids were transformed into *E. coli* BL21(DE3) for protein expression. The enzyme expressions are described in the following sections.

#### 3.2.1. Carboxylate Reductase

Codon-optimized gene encoding *Nocardia iowensis* carboxylic acid reductase (*Ni*CAR; UniProt ID: Q6RKB1.1) was cloned into the first multiple cloning site (MCS 1) of the pRSFDuet-1 vector, incorporating an N-terminal His-tag. To enable the posttranslational phosphopantetheinylation of *Ni*CAR, *Escherichia coli* phosphopantetheinyl transferase (PPTase; NCBI accession: CAQ31055.1) was co-expressed from MCS 2. Recombinant *E. coli* BL21(DE3) cells were cultivated in lysogeny broth supplemented with 100 mg/L kanamycin at 37 °C and 220 rpm until mid-log phase was reached (OD_600_ = 0.6). Protein expression was induced with 0.2 mM isopropyl-β-D-thiogalactoside (IPTG), followed by incubation at 16 °C and 220 rpm for 24 h. The cells were collected via centrifugation (7000 rpm, 15 min, 4 °C), resuspended in lysis buffer A (500 mM NaCl, 16 mM Na_2_HPO_4_, 4 mM NaH_2_PO_4_, 10 mM imidazole, 5 mM β-mercaptoethanol, pH 7.4), and lysed via sonication. Debris was removed using centrifugation (12,000 rpm, 15 min, 4 °C), and the supernatant was filtered (0.22 μm) prior to Ni-NTA affinity chromatography. The affinity column was equilibrated with lysis buffer, washed with 10 column volumes (CV) of lysis buffer, and eluted with elution buffer B (500 mM NaCl, 16 mM Na_2_HPO_4_, 4 mM NaH_2_PO_4_, 250 mM imidazole, 5 mM β-mercaptoethanol, pH 7.4). The eluted protein was concentrated via centrifugal ultrafiltration (10 kDa cutoff) and buffer-exchanged into storage buffer C (150 mM NaCl, 16 mM Na_2_HPO_4_, 4 mM NaH_2_PO_4_, 10 mM dichloro-diphenyltrichloroethane, pH 7.4) to remove imidazole. The protein concentration was quantified spectrophotometrically using Nanodrop One (Thermo Fisher Scientific, Shanghai, China), and aliquots were flash frozen in liquid nitrogen for storage at −80 °C.

*Ni*CAR activity was assayed spectrophotometrically by monitoring NADPH oxidation at 340 nm (*ε* = 6220 M^−1^ cm^−1^) in triplicate at 30 °C. Varying substrate concentrations were dissolved in 250 mM NaOH. Substrate-free controls were included for baseline correction. Kinetic parameters were derived via the nonlinear regression of Michaelis–Menten plots using OriginPro 2021 SR2 (64-bit) Build 9.8.0.240.

#### 3.2.2. Polyphosphate Kinase

His-tagged polyphosphate kinase from *Erysipelotrichaceae bacterium* (PPK12) was expressed from the pET-28a(+) vector. *E. coli* BL21 (DE3) served as the expression host. Cells were grown in LB medium, and PPK12 expression was induced by the addition of 0.2 mM IPTG at 37 °C. Cell disruption, purification, and storage procedures were performed as described for *Ni*CAR. The protein concentrations were determined using Nanodrop One (Thermo Fisher Scientific, Shanghai, China). Polyphosphate kinase activity was assessed using a spectrophotometric assay, as previously described [53].

#### 3.2.3. Pyrophosphatase

Pyrophosphatase from *Escherichia coli* (*Ec*PPase) was expressed in *E. coli* BL21(DE3) using the pET-28a(+) vector. Cells were cultivated in LB medium at 37 °C, 220 rpm, and protein expression was induced by the addition of 0.2 mM IPTG. Cell disruption, purification, and storage were performed as described for *Ni*CAR. Pyrophosphatase activity was quantified using a modified inorganic pyrophosphatase assay adapted from Sigma-Aldrich (Product Number I5907, Merck kGaA, Darmstadt, Germany).

#### 3.2.4. Glucose Dehydrogenase

His-tagged glucose dehydrogenase from *Bacillus paranthracis* (*Bp*GDH) was expressed in *E. coli* BL21(DE3) using the pET-28a(+) vector. Cells were cultivated in LB medium at 37 °C with shaking (220 rpm), and protein expression was induced at the mid-log phase (OD_600_ = 0.6) by adding 0.2 mM IPTG. Cell disruption, purification, and storage were performed as described for *Ni*CAR. *Bp*GDH activity was assayed spectrophotometrically by monitoring NADPH generation at 340 nm (*ε* = 6220 M^−1^ cm^−1^), as previously described. Reactions contained 100 mM Tris-HCl buffer (pH 7.5), 100 mM glucose, 0.2 mM NADP^+^, and purified enzyme, at 30 °C.

#### 3.2.5. Reductive Aminase

His-tagged reductive aminase from *Aspergillus oryzae* (*Asp*RedAm) was expressed in *E. coli* BL21(DE3) using the pET-28a(+) vector. The cells were cultivated in LB medium at 37 °C with shaking (220 rpm), and protein expression was induced at the mid-exponential phase (OD_600_ = 0.6) by the addition of 0.2 mM IPTG. Cell disruption, purification, and storage were performed as described for *Ni*CAR. *Asp*RedAm activity was assayed spectrophotometrically by monitoring NADPH generation at 340 nm (*ε* = 6220 M^−1^ cm^−1^), as previously described. Reactions contained 100 mM Tris-HCl buffer (pH 9.0), 10 mM hexanol, 20 mM propargylamine, 0.2 mM NADPH, and purified enzyme, at 30 °C.

### 3.3. Phase Separation Assay

#### 3.3.1. Fluorescent-Tagged Plasmid Construction

The design and construction of fluorescent fusion proteins followed the same methodology detailed in Section 3.2, with all the fluorescent proteins incorporated at the N-terminus of the fusion constructs to enable visualization of condensate formation.

#### 3.3.2. Phase Separation Confocal Imaging

Confocal imaging was performed using an inverted Nikon A1R^+^ laser scanning microscope equipped with a 100× oil-immersion objective. The samples were excited using diode lasers at 405 nm, 488 nm, and 561 nm, with corresponding dichroic mirrors and emission filters optimized for minimal spectral overlap. For imaging, a 50 μL aliquot of the sample solution was mounted on a glass slide, and the images were captured. Condensate formation assays were conducted using enzymes fused to fluorescent tags (e.g., sfGFP). Fluorescently labeled enzymes (10 μM) were incubated with 10% (*w*/*v*) PEG-8000 in 100 mM MOPS buffer (pH 7.5) at 30 °C for 15 min to induce phase separation. The real-time visualization of condensate assembly was performed using a Nikon A1R^+^ laser scanning confocal microscope equipped with a 100× oil-immersion objective. To evaluate kinetic dependencies, the equilibration times were systematically varied (5, 15, 30, and 60 min) following PEG addition. For parametric optimization, phase separation was assessed under varying pH conditions (pH 4.0, 7.5, and 10.0) and enzyme concentrations (5, 10, 20, and 40 μM), with imaging initiated after 15 min of incubation. Fluorescence intensity profiles and condensate size distributions were quantified using NIS-Elements Viewer 5.22 64-bit, with triplicate measurements performed for all experimental conditions to ensure statistical robustness.

### 3.4. Fluorescent Detection of ATP and NADPH Recycling in Condensates

#### 3.4.1. Preparation of ATP and NADPH Fluorescent Probe

The synthesis of the NADPH fluorescent probe TCF-MQ and the ATP probe PAP followed the method reported previously [51,52]. The stock solutions of TCF-MQ (2 mM) and PAP (5 mM) were prepared in DMSO (Titan Chemical, Shanghai, China) for further NADPH and ATP sensor assays.

#### 3.4.2. Fluorescent Detection of Cofactors in Condensates

The fluorescence detection of the cofactors in the condensates was performed using confocal microscopy. The concentrations in the reactions were as follows: carboxylic acid (15 mM), ATP (1.0 mM), NADPH (0.2 mM), MOPS buffer (100 mM, pH 7.5), MgCl_2_ (25 mM), β-_D_-glucose (50 mM), sodium hexametaphosphate (PolyP_6_, 8 mM), TCF-MQ (10 μM), PAP (10 μM), *Ni*CAR/BID-*Ni*CAR (0.2 U/mL), *Bp*GDH/BID-*Bp*GDH (0.2 U/mL), PPK12/BID-PPK12 (5 U/mL), and *Ec*PPase/BID-*Ec*PPase (5 U/mL), resulting in a total reaction volume of 500 μL. All reactions were shaken at 30 °C at 750 rpm in a thermomixer. After 1 h, 100 μL of the reaction mixture was mounted on a glass slide and imaged using a Nikon A1R^+^ laser scanning microscope (Nikon Instruments, Tokyo, Japan).

### 3.5. Enzyme-Catalyzed Reactions for Aldehyde Production

The concentrations in the reactions were as follows—carboxylic acid (15 mM), ATP (1.0 mM), NADPH (0.2 mM), MOPS buffer (100 mM, pH 7.5), MgCl_2_ (25 mM), β-_D_-glucose (50 mM), sodium hexametaphosphate (PolyP_6_, 8 mM), *Ni*CAR/BID-*Ni*CAR (0.2 U/mL), *Bp*GDH/BID-*Bp*GDH (0.2 U/mL), PPK12/BID-PPK12 (5 U/mL), and *Ec*PPase/BID-*Ec*PPase (5 U/mL)—resulting in a total reaction volume of 500 μL. All reactions were shaken at 30 °C at 750 rpm in a thermomixer. Samples were taken over time, and the reactions were stopped by the addition of quenching buffer (containing acetonitrile/formic acid at a 19:1 ratio). Subsequently, the samples were centrifuged (12,000 rpm, 10 min, 4 °C) and transferred to HPLC vials for measurement.

### 3.6. HPLC and GC Analysis

HPLC analysis was performed on a Shimadzu LC-2010A HT system equipped with an Agilent XDB-C18 column (5.0 μm, 4.6 × 250 mm). Mobile phases of aqueous phase A (0.5% acetic acid and 5 mM ammonium acetate) and organic phase B (acetonitrile) were used at a flow rate of 1 mL/min. The column temperature was maintained at 35 °C, and detection was conducted at 254 nm using a UV detector. A 10 μL injection volume was employed for all analyses. Quantification of conversion was performed based on UV absorbance at 254 nm using external calibration curves for benzoic acid and benzaldehyde.

GC-FID analysis was performed on a Shimadzu GC-2014 Pro gas chromatograph with a flame ionization detector (FID) equipped with a 25 m CP-Chirasil-DEX CB column with a 0.25 mm inner diameter and 0.25 μm film thickness. All samples were analyzed using a temperature program of 50 °C for 0 min, 5 °C/min until 200 °C, and then held for 2 min. Quantification was based on calibration curves using authentic standards, with peak identity confirmed through a comparison of retention times with these standards.

### 3.7. Preparation of Scale-Up Amine Products

The reaction mixture (5 mL) was prepared as follows: 10 mM carboxylic acid, 20 mM amine donor, 0.2 mM ATP, 0.04 mM NADPH, 25 mM MgCl_2_, 8 mM PolyP_6,_ 100 mM glucose, and cell lysate of all enzymes: *Asp*RedAm*/*BID-*Asp*RedAm, *Ni*CAR/BID-*Ni*CAR, *Bp*GDH/BID-*Bp*GDH, PPK12/BID-PPK12, and *Ec*PPase/BID-*Ec*PPase. All reactions were performed in 100 mM MOPS buffer, pH 7.5, at 30 °C; 250 rpm for 8 h; and then quenched using 5 M NaOH. The reaction mixture was extracted twice with equal volumes of tert-butyl methyl ether (TBME), followed by centrifugation (7000 rpm, 5 min, 4 °C) to separate phases. The organic layer was dried over anhydrous MgSO_4_ and evaporated under reduced pressure before column chromatography to obtain the final product. The structural characterization of the purified product was performed using ^1^H NMR (400 MHz) and ^13^C NMR (100 MHz) spectroscopy in CDCl_3_.

## 4. Conclusions

Cofactor regeneration presents a critical challenge in the practical application of enzymatic cascades in industrial biocatalysis, mainly attributed to the high cost and inefficient recycling of key cofactors, such as ATP and NADPH. Inspired by natural biological compartmentalization, our study employed liquid–liquid phase separation (LLPS) to construct biomimetic multienzyme condensates, aiming to overcome these limitations.

Through systematic screening, the BID domain was identified as an optimal intrinsically disordered protein (IDP) scaffold, offering favorable physicochemical properties while preserving enzyme activity. Compared to other tested IDP-*Ni*CARs, BID-*Ni*CAR exhibited significantly higher catalytic efficiency, demonstrating the importance of scaffold selection in maintaining structural integrity and enzymatic function. The experimental results displayed a 4.7-fold and 1.9-fold enhancement in ATP and NADPH recycling efficiencies, respectively, when enzymes were colocalized within BID-mediated condensates. Confocal fluorescence microscopy provided evidence of elevated local concentrations of enzymes and cofactors within the condensates, supporting the mechanism of proximity-enhanced catalysis. Importantly, the integration of the reductive aminase *Asp*RedAm into the condensates enabled the direct one-step synthesis of complex amines from carboxylic acids under cofactor concentrations reduced to one-fifth of the standard level, while achieving a 1.6-fold higher STY compared to the free enzyme system, simplifying synthetic pathways and aligning with green chemistry principles.

Overall, LLPS-enabled multienzyme condensates offer a feasible and scalable platform for overcoming the cofactor-dependent limitations inherent in conventional biocatalytic processes. The findings suggest significant potential for practical industrial implementation, encouraging future exploration into optimizing condensate conditions and scalability, and extending the methodology to diverse enzymatic systems to further enhance industrial applicability and sustainability.

## Figures and Tables

**Table 1:** Scale reactions of free enzyme and penta-enzyme condensates.

Entry	Product	Catalyst (U/mL)	System	Time (h)	Conv. (%)	Isolated Yield (%)	STY (mg L^−1^ h^−1^)
1	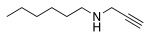	1 ^a^	free enzymes	8	83	58	144
2		1 ^b^	penta-enzyme condensates	6	>99	79	232
3	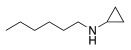	1.25 ^c^	free enzymes	8	79	50	132
4		1.25 ^d^	penta-enzyme condensates	6	90	64	212

Reaction conditions: 10 mM **1g**, 0.2 mM ATP, 0.04 mM NADPH, 25 mM MgCl_2_, 8 mM PolyP_6,_ 100 mM glucose, a: 20 mM propargylamine, *Asp*RedAm (1 U/mL), *Ni*CAR (1 U/mL), *Bp*GDH (1 U/mL), PPK12 (5 U/mL), *Ec*PPase (5 U/mL); b: 20 mM propargylamine, BID-*Asp*RedAm (1 U/mL), BID-*Ni*CAR (1 U/mL), BID-*Bp*GDH (1 U/mL), BID-PPK12 (5 U/mL), BID-*Ec*PPase (5 U/mL); c: 20 mM cyclopropylamine, *Asp*RedAm (1.25 U/mL), *Ni*CAR (1.25 U/mL), *Bp*GDH (1.25 U/mL), PPK12 (5 U/mL), *Ec*PPase (5 U/mL); d: 20 mM propargylamine, BID-*Asp*RedAm (1.25 U/mL), BID-*Ni*CAR (1.25 U/mL), BID-*Bp*GDH (1.25 U/mL), BID-PPK12 (5 U/mL), BID-*Ec*PPase (5 U/mL). All 5 mL scale-up reactions were performed in 100 mM MOPS buffer at 30 °C, 250 rpm.

## Data Availability

Data will be made available on request.

## Supplementary Materials

The following supporting information can be downloaded at: https://www.mdpi.com/article/10.3390/ijms26104795/s1.
