# Supplementary material for: Phase-Separated Multienzyme Condensates for Efficient Synthesis of Imines from Carboxylic Acids with Enhanced Dual-Cofactor Recycling
Source: Int J Mol Sci. 2025 May 16;26(10):4795. doi: 10.3390/ijms26104795 (PMC12111831; doi:10.3390/ijms26104795)
Supplement: Supplementary file 1 [file ijms-26-04795-s001.zip › ijms-3613570-supplementary.pdf]

Supporting Information

Phase-Separated Multienzyme Condensates for Efficient Synthesis of Imines from Carboxylic Acids with Enhanced Du-al-Cofactor Recycling

Tingxiao Guo 1,†, Lifang Zeng 1,†, Jiaxu Liu 1, Xiaoyan Zhang 1 and Yunpeng Bai 1,2,*

1 State Key Laboratory of Bioreactor Engineering, Shanghai Collaborative Innovation Center for Biomanufacturing, East China University of Science and Technology, Shanghai 200237, China

2 Shaanxi R&D Centre of Biomaterials and Fermentation Engineering, School of Chemical Engineering, Northwest University, Xi'an 710069, China

* Correspondence: ybai@ecust.edu.cn

† These authors contributed equally to this work.

Contents

1. Experimental procedures 3
2. Nucleotide sequence information 10
3. Figures and tables 23
4. HPLC and GC chromatograms 42
5. Calibration curves and representative HPLC traces 44
6. ^{1}H-NMR and ^{13}C-NMR 46
7. References.. 48

1. Experimental procedures

1.1. General information

ASR, BID and UTX strain were retained from the laboratory, and PopZ, *Ni*CAR, PPK12, *Ec*PPase, and *Bp*GDH were synthesized by Beijing Tsingke Biotech Co., Ltd. (Beijing, China). DNA primers were also synthesized from Beijing Tsingke Biotech Co., Ltd. (Beijing, China). All plasmid vectors, restriction enzymes, and ligases were purchased from Takara Bio (Shanghai, China). DNA gel extraction kits, plasmid extraction kits were purchased from Yeasen Biotech (Shanghai, China). Reagents for bacteria culture were purchased from Oxide and ThermoFisher (Shanghai, China). All chemicals were purchased from Titan Chemical (Shanghai, China) and used without further treatment unless otherwise indicated. NADPH and $NADP^+$ were purchased from Bontac Bioengineering (Shenzhen, China). HPLC analysis was performed on a Shimadzu LC-2010A HT apparatus equipped with an Agilent XDB-C18 column (5.0 μm, 4.6 × 250 mm). GC-FID analysis was performed on a Shimadzu GC-2014 Pro gas chromatography with a flame ionization detector (FID) equipped with a 25 m CP-Chirasil-DEX CB column with 0.25 mm inner diameter and 0.25 μm film thickness. Fluorescence images of condensate formation and cofactor recycling were captured using a in a microreactor were taken using a Nikon $A1R^+$ laser scanning confocal microscope (Japan). All experiments were performed in triplicate unless otherwise stated. Data are presented as mean ± standard deviation (SD).

1.2. Enzyme-catalyzed reactions

1.2.1. Investigation of the in vitro carboxylate reduction

The concentrations in the reactions were as follows: benzoic acid **1a** (15 mM), ATP (1.0 mM), NADPH (0.2 mM), MOPS buffer (100 mM, pH 7.5), $MgCl_2$ (25 mM), β-D-glucose (100 mM), sodium hexametaphosphate (8 mM), *Ni*CAR/ BID-*Ni*CAR (0.2 U/mL), PPK12/BID-PPK12 (5 U/mL), *Ec*PPase/BID-*Ec*PPase (5 U/mL) and *Bp*GDH/BID-*Bp*GDH (0.2 U/mL). All reactions were shaken at 30°C at 750 rpm in an Eppendorf thermomixer. Acetonitrile/formic acid 19:1 was added to aliquots to stop the reaction over due time and these solutions were analyzed by HPLC after prior centrifugation.

1. Enzyme stock solution used for the investigation of the in vitro carboxylate reduction.

Entry	Volume
*Ni*CAR/BID-*Ni*CAR	1.25 U/mL
PPK12/BID-PPK12	5 U/mL
*Ec*PPase/BID-*Ec*PPase	5 U/mL
*Bp*GDH/BID-*Bp*GDH	1.25 U/mL
Water	Up to 400 µL

2. Reaction mixture used for the investigation of the *in vitro* carboxylate reduction.

Entry	Volume
$MgCl_2$/ β-D-glucose/ MOPS buffer[1)]	250 µL
ATP/ NADPH stock solution[2)]	40 µL
Enzyme stock[3)]	80 µL
100 mM $PolyP_6$ pH 7.5[4)]	40 µL
250 mM carboxylic acid stock[5)]	30 µL
Water	60 µL
Total reaction volume	500 µL

1) $MgCl_2$ (47.6 mg, 50 mM in stock solution) + β-D-glucose (360.3 mg, 200 mM in stock solution) + 200 mM MOPS buffer (10 mL); 2) ATP (12.5 mg, 12.5 mM in stock solution) + NADPH (8.33 mg, 6.25 mM in stock solution) + water (1600 µL); 3) see **1**; 4) prepared by dissolving sodium hexametaphosphate (183.5 mg, 100 mM) in water (3.0 mL total volume); 5) carboxylic acids dissolved in 250 mM NaOH.

*1.2.2. Impact of substrate concentration on the conversion of benzoic acid **1a***

The standard reaction conditions for *in vitro* reduction of carboxylic acid **1a** were applied at increased substrate concentration. The concentrations in the reactions were as follows: benzoic acid **1a** (5, 10, 20, 50 mM), ATP (1 mM), NADPH (0.2 mM), MOPS buffer (100 mM, pH 7.5), $MgCl_2$ (25 mM), β-D-glucose (100 mM), sodium hexametaphosphate (8 mM), *Ni*CAR/BID-*Ni*CAR (0.2 U/mL), PPK12/BID-PPK12 (5 U/mL), *Ec*PPase/BID-*Ec*PPase (5 U/mL) and *Bp*GDH/BID-*Bp*GDH (0.2 U/mL). All reactions were shaken at 30°C at 750 rpm in an Eppendorf thermomixer. Acetonitrile/formic acid 19:1 was added to aliquots to stop the reaction over due time and these solutions were analyzed by HPLC after prior centrifugation.

*1.2.3. Impact of various buffers on the conversion of benzoic acid **1a***

Three distinct kinds of buffer were selected to investigate whether they have an effect on the multienzyme reaction. The concentrations in the reactions were as follows: benzoic acid **1a** (15 mM), ATP (1 mM), NADPH (0.2 mM), HEPES/Tris-HCl/MOPS buffer (100 mM, pH 7.5), $MgCl_2$ (25 mM), β-D-glucose (100 mM), sodium hexametaphosphate (8 mM), *Ni*CAR/BID-*Ni*CAR (0.2 U/mL), PPK12/BID-PPK12 (5 U/mL), *Ec*PPase/BID-*Ec*PPase (5 U/mL) and *Bp*GDH/BID-*Bp*GDH (0.2 U/mL). All reactions were shaken at 30°C at 750 rpm in an Eppendorf thermomixer. Acetonitrile/formic acid 19:1 was added to aliquots to stop the reaction over due time and these solutions were analyzed by HPLC after prior centrifugation.

*1.2.4. Impact of sodium hexametaphosphate on the conversion of benzoic acid **1a***

To estimate the limits for ATP regeneration under the general reaction conditions, further investigations with varying $PolyP_6$ concentrations were made. The concentrations in the reactions were as follows: benzoic acid **1a** (15 mM), ATP (1 mM), NADPH (0.2 mM), MOPS buffer (100 mM, pH 7.5), $MgCl_2$ (25 mM), β-D-glucose (100 mM), sodium hexametaphosphate (2, 4, 8, 20 mM), *Ni*CAR/BID-*Ni*CAR (0.2 U/mL), PPK12/BID-PPK12 (5 U/mL), *Ec*PPase/BID-*Ec*PPase (5 U/mL) and *Bp*GDH/BID-*Bp*GDH (0.2 U/mL). All reactions were shaken at 30°C at 750 rpm in an Eppendorf thermomixer. Acetonitrile/formic acid 19:1 was added to aliquots to stop the reaction over due time and these solutions were analyzed by HPLC after prior centrifugation.

*1.2.5. Impact of various ATP and NADPH concentrations on the conversion of benzoic acid **1a***

The concentrations in the reactions were as follows: benzoic acid **1a** (15 mM), ATP (100 or 1000 μM), NADPH (2, 20 or 200 μM), MOPS buffer (100 mM, pH 7.5), $MgCl_2$ (25 mM), β-D-glucose (100 mM), sodium hexametaphosphate (8 mM), *Ni*CAR/BID-*Ni*CAR(0.2 U/mL), PPK12/BID-PPK12 (5 U/mL), *Ec*PPase/BID-*Ec*PPase (5 U/mL) and *Bp*GDH/BID-*Bp*GDH (0.2 U/mL) All reactions were shaken at 30°C at 750 rpm in an Eppendorf thermomixer. Acetonitrile/formic acid 19:1 was added to aliquots to stop the reaction over due time and these solutions were analyzed by HPLC after prior centrifugation.

3. Reaction mixtures used for the investigation of the *in vitro* carboxylate reduction of **1a** under varied ATP and NADPH concentrations.

Entry	ATP (mM)	NADPH (mM)	ATP stock conc. used[1)] (mM)	NADPH stock conc. used[2)] (mM)	A/N stock used in reactions ATP (μL)	NADPH (μL)	Main stock (μL)	Water (μL)
1	1	0.2	10	1	40	80	400	280
2	1	0.02	10	1	40	8	400	352
3	1	0.002	10	1	40	0.8	400	359.2
4	0.1	0.2	10	1	4	80	400	316
5	0.1	0.02	10	1	4	8	400	388
6	0.1	0.002	10	1	4	0.8	400	395.2

1) 10 mM ATP: ATP (6 mg, 10 mM) dissolved in water (1000 μL); 2) 10 mM NADPH: NADPH (8.3 mg, 10 mM) dissolved in water (1000 μL).

1.3. Procedures for biocatalytic N-alkylation of amines with carboxylic acids

N-propargylhexylamine

To a 20 mL centrifuge tube were added hexanoic acid (10 mM, 5.81 mg), NADPH (0.04 mM, 10 mM in ddH$_2$O), ATP (0.2 mM, 10 mM in ddH$_2$O), glucose (20 mM, 1M in ddH$_2$O), MgCl$_2$ (25 mM, 1M in ddH$_2$O), PolyP$_6$ (8 mM, 500 mM in 100 mM pH 7.5 MOPS buffer), BID-*Asp*RedAm (1 U/mL), BID-*Bp*GDH (1 U/mL), BID-*Ni*CAR (1 U/mL), BID-PPK12(5 U/mL), BID-*Ec*PPase (5 U/mL) and a pH balanced stock solution of propargylamine (20 mM, 1M in 100 mM pH 7.5 MOPS buffer). The reaction volume was adjusted to 5 mL with MOPS buffer (100 mM, pH 7.5) and the tube was sealed before incubation at 30°C in a thermomixer at 250 rpm. The reaction was decanted into falcon tubes and basified to a pH of 12 with 5M NaOH solution before extraction into MTBE (2 × 10 mL) with intermediate centrifugation to improve the separation of phases (4°C, 12000 rpm, 5 min). The combined organic extracts were dried over MgSO$_4$ before concentration under reduced pressure. The crude product was

purified by column chromatography (silica, 95:5 petroleum ether: ethyl acetate) to yield the title compound as a orange oil (6.96 mg, 7.9 mM, 79%).

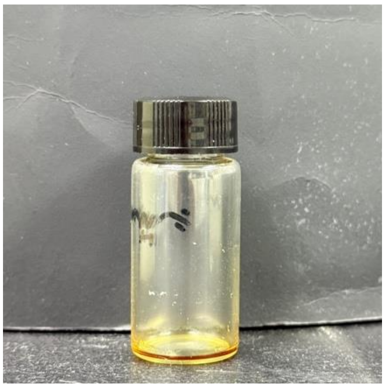

N-cyclopropylhexylamine

To a 20 mL centrifuge tube were added hexanoic acid (10 mM), NADPH (0,04 mM), ATP (0.2 mM), glucose (20 mM), $MgCl_2$ (25 mM), $PolyP_6$ (8 mM), BID-*Asp*RedAm (1 U/mL), BID-*Bp*GDH (1 U/mL), BID-*Ni*CAR (1 U/mL), BID-PPK12(5 U/mL), BID-*Ec*PPase (5 U/mL) and a pH balanced stock solution of cyclopropylamine (20 mM, 1M in 100 mM pH 7.5 MOPS buffer). The reaction volume was adjusted to 5 mL with MOPS buffer (100 mM, pH 7.5) and the tube was sealed before incubation at 30°C in a thermomixer at 250 rpm. The reaction was decanted into falcon tubes and basified to a pH of 12 with 5M NaOH solution before extraction into MTBE (2 × 10 mL) with intermediate centrifugation to improve the separation of phases (4°C, 12000 rpm, 5 min). The combined organic extracts were dried over $MgSO_4$ before concentration under reduced pressure. The crude product was purified by column chromatography (silica, 95:5 petroleum ether: ethyl acetate) to yield the title compound as a yellow oil (6.36 mg, 6.4 mM, 64%).

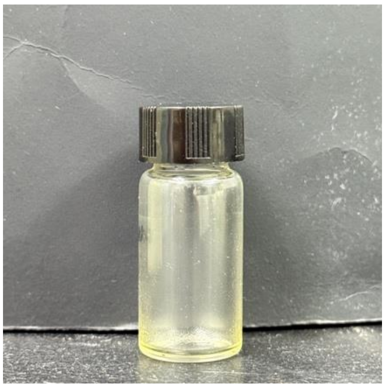

2. Nucleotide sequence information

(1) PopZ-*Ni*CAR

ATGTCTGACCAGTCTCAAGAACCGACTATGGAAGAGATTCTGGCGTCTATCCGTCGTATCAT
CTCTGAAGACGACGCTCCGGCAGAACCGGCTGCAGAAGCTGCACCGCCGCCACCACCGGA
ACCTGAACCAGAACCAGTTTCTTTCGACGACGAAGTTCTGGAACTGACCGATCCGATCGCT
CCGGAACCGGAACTGCCACCGCTGGAAACCGTTGGTGACATCGACGTTTACTCTCCGCCAG
AACCGGAATCTGAACCAGCGTACACTCCGCCACCAGCTGCTCCAGTGTTCGACCGTGACG
AAGTTGCGGAACAGCTGGTTGGTGTTTCTGCGGCATCTGCAGCTGCTTCTGCGTTCGGTTC
TCTGTCTTCTGCACTGCTGATGCCGAAAGACGGTCGTACTCTGGAAGACGTTGTTCGTGAA
CTGCTGCGTCCACTGCTGAAAGAATGGCTGGATCAGAACCTGCCGCGTATCGTTGAAACCA
AAGTTGAAGAAGAAGTTCAGCGTATCTCTCGTGGTCGTGGTGCTATGGCTGTTGATTCCCCT
GACGAACGTCTGCAACGTCGCATTGCGCAGCTGTTCGCAGAAGACGAACAGGTGAAGGCA
GCTCGTCCGCTGGAAGCGGTGAGCGCGGCAGTTAGCGCTCCAGGTATGCGCTTGGCACAG
ATTGCGGCTACTGTCATGGCGGGTTACGCAGATCGTCCGGCTGCGGGTCAACGCGCATTCG
AACTGAACACCGACGATGCCACTGGTCGCACCTCTCTCCGTCTGCTGCCGCGTTTCGAAAC
CATCACCTACCGTGAACTGTGGCAGCGTGTGGGTGAAGTTGCTGCTGCATGGCACCATGAT
CCGGAGAATCCGCTCCGTGCAGGTGACTTTGTCGCACTGCTGGGCTTCACCAGCATCGACT
ATGCGACCTTGGATTTGGCAGACATCCACCTGGGTGCAGTAACCGTTCCGCTGCAGGCGTC
TGCGGCCGTGAGCCAGCTGATCGCCATTCTGACTGAGACCAGTCCACGTCTGTTGGCATCC
ACTCCGGAACACCTGGATGCAGCTGTAGAATGTCTGCTGGCAGGTACTACTCCGGAACGTT
TGGTAGTATTCGACTACCATCCGGAAGACGACGATCAGCGTGCTGCATTCGAGAGCGCACG
TCGCCGCTTGGCAGATGCGGGTTCTTCCGTCATCGTGGAAACTCTTGATGCCGTTCGTGCTC
GTGGCCGTGATTTGCCGGCAGCACCGCTGTTTGTTCCAGATACCGATGATGATCCGCTGGCT
TTGCTGATCTACACCAGCGGTAGCACTGGTACGCCGAAAGGTGCGATGTACACTAACCGCC
TGGCAGCAACTATGTGGCAGGGTAACTCCATGCTGCAGGGCAACTCTCAACGTGTGGGTAT
CAACCTGAACTACATGCCGATGTCTCATATCGCTGGTCGTATCAGCTTGTTCGGTGTTCTGG
CTCGTGGTGGCACTGCATACTTTGCTGCAAAGAGCGACATGTCTACCTTGTTCGAAGACATC
GGCTTGGTGCGTCCAACCGAAATCTTCTTTGTTCCACGTGTCTGCGACATGGTCTTCCAACG
CTATCAGAGCGAACTGGATCGCCGTTCTGTAGCTGGTGCGGATTTGGACACTCTGGACCGT
GAAGTGAAGGCCGACCTGCGTCAGAACTACCTGGGTGGTCGCTTTCTGGTAGCAGTTGTTG
GTTCCGCTCCACTGGCTGCTGAAATGAAGACGTTCATGGAGTCCGTTCTGGACCTGCCACT
CCACGACGGCTATGGTTCTACTGAAGCCGGTGCTAGTGTTCTGCTGGACAACCAGATCCAA
CGTCCACCAGTTCTGGACTACAAACTGGTTGACGTTCCAGAACTGGGCTATTTCCGTACTG
ATCGTCCGCATCCACGTGGTGAACTGTTGCTGAAAGCTGAGACCACTATCCCTGGCTACTAC
AAACGTCCGGAAGTAACCGCTGAAATCTTCGATGAAGATGGCTTCTACAAGACCGGCGACA
TCGTAGCAGAGCTGGAACATGACCGTTTGGTTTATGTGGATCGTCGTAATAACGTGCTGAAA
CTGAGCCAGGGCGAGTTCGTGACCGTTGCGCACTTGGAAGCTGTCTTCGCGTCTTCTCCAC
TGATCCGTCAAATCTTCATCTACGGCAGCTCCGAACGTAGCTACCTGCTGGCGGTTATCGTT
CCAACTGACGATGCACTGCGTGGTCGTGACACTGCCACCTTGAAGTCTGCTCTGGCAGAAT
CCATCCAGCGCATCGCGAAAGACGCGAACTTGCAGCCATACGAAATCCCACGTGACTTTCT
GATCGAAACCGAACCATTTACGATCGCTAACGGTCTGCTGAGCGGTATTGCTAAGCTGTTGC
GTCCGAACCTGAAAGAACGTTATGGTGCTCAGCTGGAACAGATGTACACTGACCTGGCAAC

TGGTCAAGCGGATGAACTGCTGGCACTGCGTCGCGAAGCGGCTGATCTGCCGGTGTTGGA
AACTGTTAGCCGTGCAGCCAAAGCGATGCTGGGTGTTGCATCCGCTGACATGCGTCCAGAT
GCTCACTTCACCGATCTGGGTGGTGACTCTCTGAGCGCACTGTCTTTCAGCAACCTGCTCC
ACGAAATCTTTGGCGTCGAAGTTCCGGTAGGTGTAGTTGTAAGTCCAGCAAACGAACTGCG
CGATCTGGCAAACTACATTGAGGCGGAACGTAACAGCGGCGCTAAACGCCCAACGTTTACC
TCTGTACACGGCGGTGGTAGCGAAATCCGTGCTGCAGACCTGACTCTGGACAAGTTCATCG
ACGCGCGTACTTTGGCAGCAGCGGATTCCATTCCGCACGCACCAGTACCGGCTCAGACCGT
GCTGCTGACTGGTGCGAACGGTTACCTGGGTCGTTTCTTGTGCTTGGAATGGCTGGAACGT
CTGGATAAGACTGGTGGCACTCTGATCTGTGTGGTACGTGGCTCCGATGCTGCTGCTGCAC
GTAAGCGTCTGGACTCTGCTTTCGACAGCGGCGATCCGGGTCTGTTGGAACACTATCAGCA
GCTCGCTGCTCGTACCTTGGAAGTTCTGGCTGGTGACATCGGTGATCCGAACTTGGGCCTG
GATGACGCTACTTGGCAACGTCTGGCGGAAACCGTTGATCTGATCGTGCACCCGGCAGCTC
TCGTTAACCATGTGCTGCCATACACGCAACTGTTCGGTCCGAACGTTGTTGGTACGGCGGA
AATCGTGCGTCTGGCAATCACCGCGCGTCGTAAGCCGGTAACCTACCTGTCTACCGTGGGT
GTAGCAGATCAAGTGGACCCTGCTGAGTACCAAGAAGATAGCGATGTGCGTGAGATGTCTG
CGGTACGCGTTGTTCGCGAATCTTACGCTAACGGTTATGGTAACAGCAAATGGGCAGGTGA
AGTTCTGCTGCGCGAAGCTCATGACCTGTGTGGTTTGCCGGTTGCAGTATTCCGCAGCGAC
ATGATCTTGGCGCACAGCCGTTACGCAGGCCAGCTGAACGTTCAAGATGTGTTCACGCGTC
TGATCTTGAGCCTGGTTGCTACTGGCATCGCACCATATAGCTTCTATCGTACCGATGCTGATG
GTAACCGTCAGCGCGCTCATTACGACGGCCTGCCAGCCGACTTCACCGCAGCTGCTATCAC
CGCTCTGGGTATTCAGGCTACTGAGGGCTTTCGTACCTACGATGTGCTGAACCCGTATGATG
ATGGCATCAGCCTGGATGAGTTTGTGGACTGGCTTGTTGAATCTGGTCATCCGATCCAGCGT
ATCACCGATTACAGCGACTGGTTCCACCGCTTTGAAACTGCGATCCGCGCTTTGCCAGAGA
AACAGCGTCAAGCGTCTGTGTTGCCACTGCTGGACGCTTACCGCAATCCGTGTCCAGCTGT
TCGTGGTGCAATCTTGCCGGCTAAAGAATTTCAGGCTGCGGTTCAGACCGCGAAGATCGGT
CCGGAACAAGACATTCCGCATCTGAGCGCGCCGCTGATCGACAAGTACGTCTCCGACTTGG
AACTGCTCCAGCTGCTGTAA

(2) ASR-*Ni*CAR

ATGAAAAAAGTATTAGCTCTGGTTGTTGCCGCTGCTATGGGTCTGTCTTCTGCCGCCTTTGC
TGCAGAGACTACGACCACACCTGCTCCGACTGCGACGACCACCAAAGCAGCGCCGGCGAA
AACTACACATCATAAAAAACAGCATAAAGCAGCACCTGCCCAGAAAGCGCAGGCGGCTAA
AAAGCATCATAAAAATACGAAAGCTGAACAGAAAGCCCCTGAACAAAAAGCGCAGGCAGC
GAAGAAACACGCCAAGAAACACAGCCATCAGCAACCGGCAAAACCTGCTGCACAACCCG
CAGCGATGGCTGTTGATTCCCCTGACGAACGTCTGCAACGTCGCATTGCGCAGCTGTTCGC
AGAAGACGAACAGGTGAAGGCAGCTCGTCCGCTGGAAGCGGTGAGCGCGGCAGTTAGCG
CTCCAGGTATGCGCTTGGCACAGATTGCGGCTACTGTCATGGCGGGTTACGCAGATCGTCCG
GCTGCGGGTCAACGCGCATTCGAACTGAACACCGACGATGCCACTGGTCGCACCTCTCTCC
GTCTGCTGCCGCGTTTCGAAACCATCACCTACCGTGAACTGTGGCAGCGTGTGGGTGAAGT
TGCTGCTGCATGGCACCATGATCCGGAGAATCCGCTCCGTGCAGGTGACTTTGTCGCACTG
CTGGGCTTCACCAGCATCGACTATGCGACCTTGGATTTGGCAGACATCCACCTGGGTGCAG
TAACCGTTCCGCTGCAGGCGTCTGCGGCCGTGAGCCAGCTGATCGCCATTCTGACTGAGAC
CAGTCCACGTCTGTTGGCATCCACTCCGGAACACCTGGATGCAGCTGTAGAATGTCTGCTG
GCAGGTACTACTCCGGAACGTTTGGTAGTATTCGACTACCATCCGGAAGACGACGATCAGC
GTGCTGCATTCGAGAGCGCACGTCGCCGCTTGGCAGATGCGGGTTCTTCCGTCATCGTGGA

AACTCTTGATGCCGTTCGTGCTCGTGGCCGTGATTTGCCGGCAGCACCGCTGTTTGTTCCAG
ATACCGATGATGATCCGCTGGCTTTGCTGATCTACACCAGCGGTAGCACTGGTACGCCGAAA
GGTGCGATGTACACTAACCGCCTGGCAGCAACTATGTGGCAGGGTAACTCCATGCTGCAGG
GCAACTCTCAACGTGTGGGTATCAACCTGAACTACATGCCGATGTCTCATATCGCTGGTCGT
ATCAGCTTGTTCGGTGTTCTGGCTCGTGGTGGCACTGCATACTTTGCTGCAAAGAGCGACAT
GTCTACCTTGTTCGAAGACATCGGCTTGGTGCGTCCAACCGAAATCTTCTTTGTTCCACGTG
TCTGCGACATGGTCTTCCAACGCTATCAGAGCGAACTGGATCGCCGTTCTGTAGCTGGTGC
GGATTTGGACACTCTGGACCGTGAAGTGAAGGCCGACCTGCGTCAGAACTACCTGGGTGG
TCGCTTTCTGGTAGCAGTTGTTGGTTCCGCTCCACTGGCTGCTGAAATGAAGACGTTCATGG
AGTCCGTTCTGGACCTGCCACTCCACGACGGCTATGGTTCTACTGAAGCCGGTGCTAGTGT
TCTGCTGGACAACCAGATCCAACGTCCACCAGTTCTGGACTACAAACTGGTTGACGTTCCA
GAACTGGGCTATTTCCGTACTGATCGTCCGCATCCACGTGGTGAACTGTTGCTGAAAGCTG
AGACCACTATCCCTGGCTACTACAAACGTCCGGAAGTAACCGCTGAAATCTTCGATGAAGA
TGGCTTCTACAAGACCGGCGACATCGTAGCAGAGCTGGAACATGACCGTTTGGTTTATGTG
GATCGTCGTAATAACGTGCTGAAACTGAGCCAGGGCGAGTTCGTGACCGTTGCGCACTTGG
AAGCTGTCTTCGCGTCTTCTCCACTGATCCGTCAAATCTTCATCTACGGCAGCTCCGAACGT
AGCTACCTGCTGGCGGTTATCGTTCCAACTGACGATGCACTGCGTGGTCGTGACACTGCCA
CCTTGAAGTCTGCTCTGGCAGAATCCATCCAGCGCATCGCGAAAGACGCGAACTTGCAGCC
ATACGAAATCCCACGTGACTTTCTGATCGAAACCGAACCATTTACGATCGCTAACGGTCTGC
TGAGCGGTATTGCTAAGCTGTTGCGTCCGAACCTGAAAGAACGTTATGGTGCTCAGCTGGA
ACAGATGTACACTGACCTGGCAACTGGTCAAGCGGATGAACTGCTGGCACTGCGTCGCGA
AGCGGCTGATCTGCCGGTGTTGGAAACTGTTAGCCGTGCAGCCAAAGCGATGCTGGGTGTT
GCATCCGCTGACATGCGTCCAGATGCTCACTTCACCGATCTGGGTGGTGACTCTCTGAGCG
CACTGTCTTTCAGCAACCTGCTCCACGAAATCTTTGGCGTCGAAGTTCCGGTAGGTGTAGTT
GTAAGTCCAGCAAACGAACTGCGCGATCTGGCAAACTACATTGAGGCGGAACGTAACAGC
GGCGCTAAACGCCCAACGTTTACCTCTGTACACGGCGGTGGTAGCGAAATCCGTGCTGCAG
ACCTGACTCTGGACAAGTTCATCGACGCGCGTACTTTGGCAGCAGCGGATTCCATTCCGCA
CGCACCAGTACCGGCTCAGACCGTGCTGCTGACTGGTGCGAACGGTTACCTGGGTCGTTTC
TTGTGCTTGGAATGGCTGGAACGTCTGGATAAGACTGGTGGCACTCTGATCTGTGTGGTAC
GTGGCTCCGATGCTGCTGCTGCACGTAAGCGTCTGGACTCTGCTTTCGACAGCGGCGATCC
GGGTCTGTTGGAACACTATCAGCAGCTCGCTGCTCGTACCTTGGAAGTTCTGGCTGGTGAC
ATCGGTGATCCGAACTTGGGCCTGGATGACGCTACTTGGCAACGTCTGGCGGAAACCGTTG
ATCTGATCGTGCACCCGGCAGCTCTCGTTAACCATGTGCTGCCATACACGCAACTGTTCGGT
CCGAACGTTGTTGGTACGGCGGAAATCGTGCGTCTGGCAATCACCGCGCGTCGTAAGCCGG
TAACCTACCTGTCTACCGTGGGTGTAGCAGATCAAGTGGACCCTGCTGAGTACCAAGAAGA
TAGCGATGTGCGTGAGATGTCTGCGGTACGCGTTGTTCGCGAATCTTACGCTAACGGTTATG
GTAACAGCAAATGGGCAGGTGAAGTTCTGCTGCGCGAAGCTCATGACCTGTGTGGTTTGCC
GGTTGCAGTATTCCGCAGCGACATGATCTTGGCGCACAGCCGTTACGCAGGCCAGCTGAAC
GTTCAAGATGTGTTCACGCGTCTGATCTTGAGCCTGGTTGCTACTGGCATCGCACCATATAG
CTTCTATCGTACCGATGCTGATGGTAACCGTCAGCGCGCTCATTACGACGGCCTGCCAGCCG
ACTTCACCGCAGCTGCTATCACCGCTCTGGGTATTCAGGCTACTGAGGGCTTTCGTACCTAC
GATGTGCTGAACCCGTATGATGATGGCATCAGCCTGGATGAGTTTGTGGACTGGCTTGTTGA
ATCTGGTCATCCGATCCAGCGTATCACCGATTACAGCGACTGGTTCCACCGCTTTGAAACTG
CGATCCGCGCTTTGCCAGAGAAACAGCGTCAAGCGTCTGTGTTGCCACTGCTGGACGCTTA

CCGCAATCCGTGTCCAGCTGTTCGTGGTGCAATCTTGCCGGCTAAAGAATTTCAGGCTGCG
GTTCAGACCGCGAAGATCGGTCCGGAACAAGACATTCCGCATCTGAGCGCGCCGCTGATCG
ACAAGTACGTCTCCGACTTGGAACTGCTCCAGCTGCTGTAA

(3) UTX-*Ni*CAR

GTGCCTAGCGTCTCTCAGCCTGGAGTCCGTCCTGCCTGCCCTGGGCAGCCTTTGGCCAATG
GACCCTTTTCTGCAGGCCATGTTCCCTGTAGCACATCAAGAACGCTGGGAAGTACAGACAC
TATTTTGATAGGCAATAATCATATAACAGGAAGTGGAAGTAATGGAAACGTGCCTTACCTGC
AGCGAAACGCACTCACTCTACCTCATAACCGCACAAACCTGACCAGCAGCGCAGAGGAGC
CGTGGAAAAACCAACTATCTAACTCCACTCAGGGGCTTCACAAAGGTCAGAGTTCACATTC
GGCAGGTCCTAATGGTGAACGACCTCTCTCTTCCACTGGGCCTTCCCAGCATCTCCAGGCA
GCTGGCTCTGGTATTCAGAATCAGAACGGACATCCCACCCTGCCTAGCAATTCAGTAACAC
AGGGGGCTGCTCTCAATCACCTCTCCTCTCACACTGCTACCTCAGGTGGACAACAAGGCAT
TACCTTAACCAAAGAGAGCAAGCCTTCAGGAAACATATTGACGGTGCCTGAAACAAGCAG
GCACACTGGAGAGACACCTAACAGCACTGCCAGTGTCGAGGGACTTCCTAATCATGTCCAT
CAGATGACGGCAGATGCTGTTTGCAGTCCTAGCCATGGAGATTCTAAGTCACCAGGTTTACT
AAGTTCAGACAATCCTCAGCTCTCTGCCTTGTTGATGGGAAAAGCCAATAACAATGTGGGT
ACTGGAACCTGTGACAAAGTCAATAACATCCACCCAGCTGTTCATACAAAGACTGATAACT
CTGTTGCCTCTTCACCATCTTCAGCCATTTCAACAGCAACACCTTCTCCAAAATCCACTGAG
CAGACAACCACAAACAGTGTTACCAGCCTTAACAGCCCTCACGGAGGATCAGGAGGATCA
GGAGGATCAATGGCTGTTGATTCCCCTGACGAACGTCTGCAACGTCGCATTGCGCAGCTGT
TCGCAGAAGACGAACAGGTGAAGGCAGCTCGTCCGCTGGAAGCGGTGAGCGCGGCAGTT
AGCGCTCCAGGTATGCGCTTGGCACAGATTGCGGCTACTGTCATGGCGGGTTACGCAGATC
GTCCGGCTGCGGGTCAACGCGCATTCGAACTGAACACCGACGATGCCACTGGTCGCACCTC
TCTCCGTCTGCTGCCGCGTTTCGAAACCATCACCTACCGTGAACTGTGGCAGCGTGTGGGT
GAAGTTGCTGCTGCATGGCACCATGATCCGGAGAATCCGCTCCGTGCAGGTGACTTTGTCG
CACTGCTGGGCTTCACCAGCATCGACTATGCGACCTTGGATTTGGCAGACATCCACCTGGG
TGCAGTAACCGTTCCGCTGCAGGCGTCTGCGGCCGTGAGCCAGCTGATCGCCATTCTGACT
GAGACCAGTCCACGTCTGTTGGCATCCACTCCGGAACACCTGGATGCAGCTGTAGAATGTC
TGCTGGCAGGTACTACTCCGGAACGTTTGGTAGTATTCGACTACCATCCGGAAGACGACGA
TCAGCGTGCTGCATTCGAGAGCGCACGTCGCCGCTTGGCAGATGCGGGTTCTTCCGTCATC
GTGGAAACTCTTGATGCCGTTCGTGCTCGTGGCCGTGATTTGCCGGCAGCACCGCTGTTTG
TTCCAGATACCGATGATGATCCGCTGGCTTTGCTGATCTACACCAGCGGTAGCACTGGTACG
CCGAAAGGTGCGATGTACACTAACCGCCTGGCAGCAACTATGTGGCAGGGTAACTCCATGC
TGCAGGGCAACTCTCAACGTGTGGGTATCAACCTGAACTACATGCCGATGTCTCATATCGCT
GGTCGTATCAGCTTGTTCGGTGTTCTGGCTCGTGGTGGCACTGCATACTTTGCTGCAAAGAG
CGACATGTCTACCTTGTTCGAAGACATCGGCTTGGTGCGTCCAACCGAAATCTTCTTTGTTC
CACGTGTCTGCGACATGGTCTTCCAACGCTATCAGAGCGAACTGGATCGCCGTTCTGTAGCT
GGTGCGGATTTGGACACTCTGGACCGTGAAGTGAAGGCCGACCTGCGTCAGAACTACCTG
GGTGGTCGCTTTCTGGTAGCAGTTGTTGGTTCCGCTCCACTGGCTGCTGAAATGAAGACGT
TCATGGAGTCCGTTCTGGACCTGCCACTCCACGACGGCTATGGTTCTACTGAAGCCGGTGC
TAGTGTTCTGCTGGACAACCAGATCCAACGTCCACCAGTTCTGGACTACAAACTGGTTGAC
GTTCCAGAACTGGGCTATTTCCGTACTGATCGTCCGCATCCACGTGGTGAACTGTTGCTGAA
AGCTGAGACCACTATCCCTGGCTACTACAAACGTCCGGAAGTAACCGCTGAAATCTTCGAT
GAAGATGGCTTCTACAAGACCGGCGACATCGTAGCAGAGCTGGAACATGACCGTTTGGTTT

ATGTGGATCGTCGTAATAACGTGCTGAAACTGAGCCAGGGCGAGTTCGTGACCGTTGCGCA
CTTGGAAGCTGTCTTCGCGTCTTCTCCACTGATCCGTCAAATCTTCATCTACGGCAGCTCCG
AACGTAGCTACCTGCTGGCGGTTATCGTTCCAACTGACGATGCACTGCGTGGTCGTGACAC
TGCCACCTTGAAGTCTGCTCTGGCAGAATCCATCCAGCGCATCGCGAAAGACGCGAACTTG
CAGCCATACGAAATCCCACGTGACTTTCTGATCGAAACCGAACCATTTACGATCGCTAACGG
TCTGCTGAGCGGTATTGCTAAGCTGTTGCGTCCGAACCTGAAAGAACGTTATGGTGCTCAG
CTGGAACAGATGTACACTGACCTGGCAACTGGTCAAGCGGATGAACTGCTGGCACTGCGT
CGCGAAGCGGCTGATCTGCCGGTGTTGGAAACTGTTAGCCGTGCAGCCAAAGCGATGCTG
GGTGTTGCATCCGCTGACATGCGTCCAGATGCTCACTTCACCGATCTGGGTGGTGACTCTCT
GAGCGCACTGTCTTTCAGCAACCTGCTCCACGAAATCTTTGGCGTCGAAGTTCCGGTAGGT
GTAGTTGTAAGTCCAGCAAACGAACTGCGCGATCTGGCAAACTACATTGAGGCGGAACGTA
ACAGCGGCGCTAAACGCCCAACGTTTACCTCTGTACACGGCGGTGGTAGCGAAATCCGTGC
TGCAGACCTGACTCTGGACAAGTTCATCGACGCGCGTACTTTGGCAGCAGCGGATTCCATT
CCGCACGCACCAGTACCGGCTCAGACCGTGCTGCTGACTGGTGCGAACGGTTACCTGGGTC
GTTTCTTGTGCTTGGAATGGCTGGAACGTCTGGATAAGACTGGTGGCACTCTGATCTGTGTG
GTACGTGGCTCCGATGCTGCTGCTGCACGTAAGCGTCTGGACTCTGCTTTCGACAGCGGCG
ATCCGGGTCTGTTGGAACACTATCAGCAGCTCGCTGCTCGTACCTTGGAAGTTCTGGCTGGT
GACATCGGTGATCCGAACTTGGGCCTGGATGACGCTACTTGGCAACGTCTGGCGGAAACCG
TTGATCTGATCGTGCACCCGGCAGCTCTCGTTAACCATGTGCTGCCATACACGCAACTGTTC
GGTCCGAACGTTGTTGGTACGGCGGAAATCGTGCGTCTGGCAATCACCGCGCGTCGTAAGC
CGGTAACCTACCTGTCTACCGTGGGTGTAGCAGATCAAGTGGACCCTGCTGAGTACCAAGA
AGATAGCGATGTGCGTGAGATGTCTGCGGTACGCGTTGTTCGCGAATCTTACGCTAACGGTT
ATGGTAACAGCAAATGGGCAGGTGAAGTTCTGCTGCGCGAAGCTCATGACCTGTGTGGTTT
GCCGGTTGCAGTATTCCGCAGCGACATGATCTTGGCGCACAGCCGTTACGCAGGCCAGCTG
AACGTTCAAGATGTGTTCACGCGTCTGATCTTGAGCCTGGTTGCTACTGGCATCGCACCATA
TAGCTTCTATCGTACCGATGCTGATGGTAACCGTCAGCGCGCTCATTACGACGGCCTGCCAG
CCGACTTCACCGCAGCTGCTATCACCGCTCTGGGTATTCAGGCTACTGAGGGCTTTCGTACC
TACGATGTGCTGAACCCGTATGATGATGGCATCAGCCTGGATGAGTTTGTGGACTGGCTTGT
TGAATCTGGTCATCCGATCCAGCGTATCACCGATTACAGCGACTGGTTCCACCGCTTTGAAA
CTGCGATCCGCGCTTTGCCAGAGAAACAGCGTCAAGCGTCTGTGTTGCCACTGCTGGACGC
TTACCGCAATCCGTGTCCAGCTGTTCGTGGTGCAATCTTGCCGGCTAAAGAATTTCAGGCTG
CGGTTCAGACCGCGAAGATCGGTCCGGAACAAGACATTCCGCATCTGAGCGCGCCGCTGAT
CGACAAGTACGTCTCCGACTTGGAACTGCTCCAGCTGCTGTAA

(4) BID-*Ni*CAR

ATGGATTGTGAAGTTAACAACGGTAGCAGCCTGCGTGATGAATGTATTACCAATCTGCTGGT
TTTCGGTTTTCTGCAGAGCTGTAGCGATAATAGCTTTCGTCGTGAACTGGATGCGCTGGGTC
ATGAACTGCCGGTGCTGGCACCTCAGTGGGAAGGTTATGATGAACTGCAGACCGATGGTAA
TCGTAGCAGCCATTCTCGTCTGGGTCGTATTGAAGCAGATTCTGAAAGCCAGGAAGATATTA
TTCGTAATATTGCACGTCATCTGGCACAGGTGGGTGATAGCATGGATCGTAGTATCCCGCCG
GGTCTGGTTAATGGTCTGGCACTGCAGCTGCGTAATACCAGCCGTAGCGAAGAAGATCGTA
ACCGCGATCTGGCAACCGCACTGGAACAGCTGCTGCAGGCATATCCGCGCGATATGGAAAA
GGAAAAGACAATGCTGGTCCTGGCACTGCTGCTGGCAAAGAAGGTGGCAAGTCATACACC
TAGCCTGCTGCGCGATGTTTTCCATACCACCGTTAATTTTATTAACCAGAACCTGCGCACGTA
TGTTCGTAGCCTGGCTCGTAATGGTATGGATATGGCTGTTGATTCCCCTGACGAACGTCTGC

AACGTCGCATTGCGCAGCTGTTCGCAGAAGACGAACAGGTGAAGGCAGCTCGTCCGCTGG
AAGCGGTGAGCGCGGCAGTTAGCGCTCCAGGTATGCGCTTGGCACAGATTGCGGCTACTGT
CATGGCGGGTTACGCAGATCGTCCGGCTGCGGGTCAACGCGCATTCGAACTGAACACCGAC
GATGCCACTGGTCGCACCTCTCTCCGTCTGCTGCCGCGTTTCGAAACCATCACCTACCGTGA
ACTGTGGCAGCGTGTGGGTGAAGTTGCTGCTGCATGGCACCATGATCCGGAGAATCCGCTC
CGTGCAGGTGACTTTGTCGCACTGCTGGGCTTCACCAGCATCGACTATGCGACCTTGGATTT
GGCAGACATCCACCTGGGTGCAGTAACCGTTCCGCTGCAGGCGTCTGCGGCCGTGAGCCA
GCTGATCGCCATTCTGACTGAGACCAGTCCACGTCTGTTGGCATCCACTCCGGAACACCTG
GATGCAGCTGTAGAATGTCTGCTGGCAGGTACTACTCCGGAACGTTTGGTAGTATTCGACTA
CCATCCGGAAGACGACGATCAGCGTGCTGCATTCGAGAGCGCACGTCGCCGCTTGGCAGAT
GCGGGTTCTTCCGTCATCGTGGAAACTCTTGATGCCGTTCGTGCTCGTGGCCGTGATTTGCC
GGCAGCACCGCTGTTTGTTCCAGATACCGATGATGATCCGCTGGCTTTGCTGATCTACACCA
GCGGTAGCACTGGTACGCCGAAAGGTGCGATGTACACTAACCGCCTGGCAGCAACTATGTG
GCAGGGTAACTCCATGCTGCAGGGCAACTCTCAACGTGTGGGTATCAACCTGAACTACATG
CCGATGTCTCATATCGCTGGTCGTATCAGCTTGTTCGGTGTTCTGGCTCGTGGTGGCACTGC
ATACTTTGCTGCAAAGAGCGACATGTCTACCTTGTTCGAAGACATCGGCTTGGTGCGTCCA
ACCGAAATCTTCTTTGTTCCACGTGTCTGCGACATGGTCTTCCAACGCTATCAGAGCGAACT
GGATCGCCGTTCTGTAGCTGGTGCGGATTTGGACACTCTGGACCGTGAAGTGAAGGCCGAC
CTGCGTCAGAACTACCTGGGTGGTCGCTTTCTGGTAGCAGTTGTTGGTTCCGCTCCACTGG
CTGCTGAAATGAAGACGTTCATGGAGTCCGTTCTGGACCTGCCACTCCACGACGGCTATGG
TTCTACTGAAGCCGGTGCTAGTGTTCTGCTGGACAACCAGATCCAACGTCCACCAGTTCTG
GACTACAAACTGGTTGACGTTCCAGAACTGGGCTATTTCCGTACTGATCGTCCGCATCCACG
TGGTGAACTGTTGCTGAAAGCTGAGACCACTATCCCTGGCTACTACAAACGTCCGGAAGTA
ACCGCTGAAATCTTCGATGAAGATGGCTTCTACAAGACCGGCGACATCGTAGCAGAGCTGG
AACATGACCGTTTGGTTTATGTGGATCGTCGTAATAACGTGCTGAAACTGAGCCAGGGCGA
GTTCGTGACCGTTGCGCACTTGGAAGCTGTCTTCGCGTCTTCTCCACTGATCCGTCAAATCT
TCATCTACGGCAGCTCCGAACGTAGCTACCTGCTGGCGGTTATCGTTCCAACTGACGATGCA
CTGCGTGGTCGTGACACTGCCACCTTGAAGTCTGCTCTGGCAGAATCCATCCAGCGCATCG
CGAAAGACGCGAACTTGCAGCCATACGAAATCCCACGTGACTTTCTGATCGAAACCGAACC
ATTTACGATCGCTAACGGTCTGCTGAGCGGTATTGCTAAGCTGTTGCGTCCGAACCTGAAAG
AACGTTATGGTGCTCAGCTGGAACAGATGTACACTGACCTGGCAACTGGTCAAGCGGATGA
ACTGCTGGCACTGCGTCGCGAAGCGGCTGATCTGCCGGTGTTGGAAACTGTTAGCCGTGCA
GCCAAAGCGATGCTGGGTGTTGCATCCGCTGACATGCGTCCAGATGCTCACTTCACCGATCT
GGGTGGTGACTCTCTGAGCGCACTGTCTTTCAGCAACCTGCTCCACGAAATCTTTGGCGTC
GAAGTTCCGGTAGGTGTAGTTGTAAGTCCAGCAAACGAACTGCGCGATCTGGCAAACTACA
TTGAGGCGGAACGTAACAGCGGCGCTAAACGCCCAACGTTTACCTCTGTACACGGCGGTG
GTAGCGAAATCCGTGCTGCAGACCTGACTCTGGACAAGTTCATCGACGCGCGTACTTTGGC
AGCAGCGGATTCCATTCCGCACGCACCAGTACCGGCTCAGACCGTGCTGCTGACTGGTGCG
AACGGTTACCTGGGTCGTTTCTTGTGCTTGGAATGGCTGGAACGTCTGGATAAGACTGGTG
GCACTCTGATCTGTGTGGTACGTGGCTCCGATGCTGCTGCTGCACGTAAGCGTCTGGACTCT
GCTTTCGACAGCGGCGATCCGGGTCTGTTGGAACACTATCAGCAGCTCGCTGCTCGTACCT
TGGAAGTTCTGGCTGGTGACATCGGTGATCCGAACTTGGGCCTGGATGACGCTACTTGGCA
ACGTCTGGCGGAAACCGTTGATCTGATCGTGCACCCGGCAGCTCTCGTTAACCATGTGCTG
CCATACACGCAACTGTTCGGTCCGAACGTTGTTGGTACGGCGGAAATCGTGCGTCTGGCAA

TCACCGCGCGTCGTAAGCCGGTAACCTACCTGTCTACCGTGGGTGTAGCAGATCAAGTGGA
CCCTGCTGAGTACCAAGAAGATAGCGATGTGCGTGAGATGTCTGCGGTACGCGTTGTTCGC
GAATCTTACGCTAACGGTTATGGTAACAGCAAATGGGCAGGTGAAGTTCTGCTGCGCGAAG
CTCATGACCTGTGTGGTTTGCCGGTTGCAGTATTCCGCAGCGACATGATCTTGGCGCACAGC
CGTTACGCAGGCCAGCTGAACGTTCAAGATGTGTTCACGCGTCTGATCTTGAGCCTGGTTG
CTACTGGCATCGCACCATATAGCTTCTATCGTACCGATGCTGATGGTAACCGTCAGCGCGCTC
ATTACGACGGCCTGCCAGCCGACTTCACCGCAGCTGCTATCACCGCTCTGGGTATTCAGGCT
ACTGAGGGCTTTCGTACCTACGATGTGCTGAACCCGTATGATGATGGCATCAGCCTGGATGA
GTTTGTGGACTGGCTTGTTGAATCTGGTCATCCGATCCAGCGTATCACCGATTACAGCGACT
GGTTCCACCGCTTTGAAACTGCGATCCGCGCTTTGCCAGAGAAACAGCGTCAAGCGTCTGT
GTTGCCACTGCTGGACGCTTACCGCAATCCGTGTCCAGCTGTTCGTGGTGCAATCTTGCCG
GCTAAAGAATTTCAGGCTGCGGTTCAGACCGCGAAGATCGGTCCGGAACAAGACATTCCG
CATCTGAGCGCGCCGCTGATCGACAAGTACGTCTCCGACTTGGAACTGCTCCAGCTGCTGT
AA

(5) BID-PPK12

ATGGATTGTGAAGTTAACAACGGTAGCAGCCTGCGTGATGAATGTATTACCAATCTGCTGGT
TTTCGGTTTTCTGCAGAGCTGTAGCGATAATAGCTTTCGTCGTGAACTGGATGCGCTGGGTC
ATGAACTGCCGGTGCTGGCACCTCAGTGGGAAGGTTATGATGAACTGCAGACCGATGGTAA
TCGTAGCAGCCATTCTCGTCTGGGTCGTATTGAAGCAGATTCTGAAAGCCAGGAAGATATTA
TTCGTAATATTGCACGTCATCTGGCACAGGTGGGTGATAGCATGGATCGTAGTATCCCGCCG
GGTCTGGTTAATGGTCTGGCACTGCAGCTGCGTAATACCAGCCGTAGCGAAGAAGATCGTA
ACCGCGATCTGGCAACCGCACTGGAACAGCTGCTGCAGGCATATCCGCGCGATATGGAAAA
GGAAAAGACAATGCTGGTCCTGGCACTGCTGCTGGCAAAGAAGGTGGCAAGTCATACACC
TAGCCTGCTGCGCGATGTTTTCCATACCACCGTTAATTTTATTAACCAGAACCTGCGCACGTA
TGTTCGTAGCCTGGCTCGTAATGGTATGGATATGATCAACATCTACAAGATCGATAAACTGAA
CAACTTCAACCTGAACAACCACAAGACCGACGATTACAGCCTGTGCAAAGACAAAGATAC
CGCGCTGGAACTGACTCAGAAGAACATCCAGAAGATCTACGACTACCAGCAGAAACTGTA
CGCGGAGAAGAAAGAAGGTCTGATCATCGCGTTCCAGGCGATGGATGCGGCTGGTAAAGA
CGGTACTATCCGTGAAGTGTTGAAAGCTCTGGCTCCGCAGGGTGTTCACGAGAAACCATTC
AAATCTCCATCTTCCACCGAACTGGCGCACGACTACCTGTGGCGTGTTCACAACGCAGTAC
CGGAGAAAGGTGAAATCACCATCTTCAACCGTTCTCACTACGAAGATGTTCTGATCGGCAA
AGTTAAAGAACTGTACAAATTCCAGAACAAAGCTGACCGTATCGATGAGAACACCGTTGTG
GACAACCGCTACGAAGATATCCGTAACTTCGAGAAATACCTGTACAACAACTCTGTTCGTAT
CATCAAGATCTTTCTGAACGTTTCTAAAAAAGAACAGGCTGAACGCTTTCTGAGCCGTATC
GAAGAACCAGAGAAGAACTGGAAGTTCTCTGACTCCGACTTCGAAGAACGTGTATACTGG
GACAAATACCAGCAGGCGTTCGAAGACGCGATCAACGCGACCTCCACCAAAGACTGTCCG
TGGTATGTTGTTCCGGCTGATCGTAAATGGTACATGCGTTACGTGGTGTCTGAAATCGTAGTT
AAGACCTTGGAAGAAATGAATCCGAAATATCCGACCGTTACCAAAGAAACTCTGGAACGTT
TCGAAGGTTACCGTACCAAACTGCTGGAAGAATACAACTACGATCTGGACACCATCCGTCC
GATCGAGAAG

(3) BID-*Ec*PPase

ATGGATTGTGAAGTTAACAACGGTAGCAGCCTGCGTGATGAATGTATTACCAATCTGCTGGT
TTTCGGTTTTCTGCAGAGCTGTAGCGATAATAGCTTTCGTCGTGAACTGGATGCGCTGGGTC
ATGAACTGCCGGTGCTGGCACCTCAGTGGGAAGGTTATGATGAACTGCAGACCGATGGTAA

TCGTAGCAGCCATTCTCGTCTGGGTCGTATTGAAGCAGATTCTGAAAGCCAGGAAGATATTA
TTCGTAATATTGCACGTCATCTGGCACAGGTGGGTGATAGCATGGATCGTAGTATCCCGCCG
GGTCTGGTTAATGGTCTGGCACTGCAGCTGCGTAATACCAGCCGTAGCGAAGAAGATCGTA
ACCGCGATCTGGCAACCGCACTGGAACAGCTGCTGCAGGCATATCCGCGCGATATGGAAAA
GGAAAAGACAATGCTGGTCCTGGCACTGCTGCTGGCAAAGAAGGTGGCAAGTCATACACC
TAGCCTGCTGCGCGATGTTTTCCATACCACCGTTAATTTTATTAACCAGAACCTGCGCACGTA
TGTTCGTAGCCTGGCTCGTAATGGTATGGATATGAGCCTGCTGAACGTTCCGGCGGGTAAAG
ACCTGCCGGAAGACATCTACGTGGTTATCGAAATCCCAGCAAACGCTGATCCGATCAAATA
CGAGATCGACAAAGAAAGCGGTGCGCTGTTCGTAGATCGTTTCATGTCCACCGCGATGTTC
TATCCGTGCAACTACGGTTACATCAACCACACCTTGTCTCTGGACGGTGATCCGGTTGATGT
GCTGGTTCCGACGCCGTATCCGTTGCAGCCGGGTTCCGTTATCCGTTGTCGTCCGGTTGGTG
TGCTGAAGATGACTGATGAAGCTGGTGAAGACGCTAAACTGATCGCTGTTCCGCACACCAA
ACTGTCTAAAGAATACGACCACATCAAAGACGTTAACGATCTGCCGGAACTGCTGAAAGCG
CAGATCGCTCACTTCTTCGAACACTACAAAGATCTGGAGAAAGGTAAGTGGGTTAAAGTTG
AAGGCTGGGAGAACGCAGAAGCTGCTAAAGCAGAAATCGTTGCGTCTTTCGAACGTGCTA
AGAACAAG

(6) BID-*Bp*GDH

ATGGATTGTGAAGTTAACAACGGTAGCAGCCTGCGTGATGAATGTATTACCAATCTGCTGGT
TTTCGGTTTTCTGCAGAGCTGTAGCGATAATAGCTTTCGTCGTGAACTGGATGCGCTGGGTC
ATGAACTGCCGGTGCTGGCACCTCAGTGGGAAGGTTATGATGAACTGCAGACCGATGGTAA
TCGTAGCAGCCATTCTCGTCTGGGTCGTATTGAAGCAGATTCTGAAAGCCAGGAAGATATTA
TTCGTAATATTGCACGTCATCTGGCACAGGTGGGTGATAGCATGGATCGTAGTATCCCGCCG
GGTCTGGTTAATGGTCTGGCACTGCAGCTGCGTAATACCAGCCGTAGCGAAGAAGATCGTA
ACCGCGATCTGGCAACCGCACTGGAACAGCTGCTGCAGGCATATCCGCGCGATATGGAAAA
GGAAAAGACAATGCTGGTCCTGGCACTGCTGCTGGCAAAGAAGGTGGCAAGTCATACACC
TAGCCTGCTGCGCGATGTTTTCCATACCACCGTTAATTTTATTAACCAGAACCTGCGCACGTA
TGTTCGTAGCCTGGCTCGTAATGGTATGGATATGTACAGTGATCTGGAAGGTAAAGTGGTGG
TGATTACCGGCAGCGCCAGTGGTCTGGGCCGCGCAATGGGCGTTCGCTTTGCCCGCGAAAA
AGCAAAAGTGGTGATTAATTATCGTAGTCGTGAAAGCGAAGCCAATGATGTGCTGGAAGAA
ATTAAAAAAGTGGGTGGTGAAGCAATTGCAGTTAAAGGCGATGTGACCGTTGAAAGTGATG
TTGTTAATCTGATTCAGAGTGCCGTGAAAGAATTTGGTACCCTGGATGTGATGATTAATAATG
CAGGTATTGAAAACGCAGTTCCGAGCCATGAAATGCCGCTGGAAGATTGGAATCGTGTTAT
TAATACCAATCTGACCGGCGCATTTCTGGGCAGTCGCGAAGCCATTAAATATTTTGTTGAAC
ATGACATCAAGGGCAGCGTTATTAATATGAGCAGCGTTCATGAAAAAATCCCGTGGCCGCTG
TTTGTTCATTATGCCGCAAGCAAAGGCGGTATGAAACTGATGACCGAAACCCTGGCCATGG
AATATGCACCGAAAGGTATTCGCGTGAATAATATTGGCCCGGGTGCAATTAATACCCCGATTA
ATGCAGAAAAATTCGCCGATCCGAAAAAACGCGCCGATGTGGAAAGCATGATTCCGATGGG
CTATATTGGCAAACCGGAAGAAATTGCAGCCGTGGCAACCTGGCTGGCCAGCAGCGAAGC
CAGTTATGTTACCGGCATTACCCTGTTTGCAGATGGCGGCATGACCCTGTATCCGAGTTTTCA
GGCCGGTCGCGGTTAA

(7) BFP-BID-*Ec*PPase

CATATGAGCGAACTGATTAAAGAAAACATGCACATGAAACTGTACATGGAAGGGACGGTGG
ACAACCACCACTTTAAATGCACCAGCGAAGGAGAAGGAAAACCGTACGAAGGAACCCAG
ACCATGCGGATTAAAGTGGTGGAAGGCGGACCGCTGCCGTTTGCGTTTGATATTCTGGCAA

CCAGTTTTCTGTACGGTAGCAAAACCTTTATAAATCACACCCAGGGAATACCGGATTTTTTT
AAACAGTCCTTTCCGGAAGGTTTTACCTGGGAACGTGTTACCACGTACGAAGATGGCGGAG
TTCTGACCGCAACACAAGACACCTCACTGCAGGACGGCTGTCTGATATATAATGTTAAAATT
CGCGGTGTTAACTTCACCTCAAATGGACCTGTTATGCAAAAGAAAACTCTGGGTTGGGAAG
CATTTACCGAAACGCTGTATCCTGCAGATGGTGGTCTGGAAGGCCGTAATGACATGGCACT
GAAACTGGTTGGCGGTTCTCATCTGATTGCCAATATCAAAACAACCTATAGAAGCAAAAAG
CCGGCAAAAAATCTGAAAATGCCGGGTGTTTATTATGTTGATTATCGTCTGGAACGTATTAA
AGAAGCAAATAATGAAACCTATGTTGAACAGCATGAAGTTGCAGTTGCACGTTATTGTGATC
TGCCGAGCAAACTGGGTCATAAACTGAATCTCGAGATGGATTGTGAAGTTAACAACGGTAG
CAGCCTGCGTGATGAATGTATTACCAATCTGCTGGTTTTCGGTTTTCTGCAGAGCTGTAGCG
ATAATAGCTTTCGTCGTGAACTGGATGCGCTGGGTCATGAACTGCCGGTGCTGGCACCTCA
GTGGGAAGGTTATGATGAACTGCAGACCGATGGTAATCGTAGCAGCCATTCTCGTCTGGGT
CGTATTGAAGCAGATTCTGAAAGCCAGGAAGATATTATTCGTAATATTGCACGTCATCTGGC
ACAGGTGGGTGATAGCATGGATCGTAGTATCCCGCCGGGTCTGGTTAATGGTCTGGCACTGC
AGCTGCGTAATACCAGCCGTAGCGAAGAAGATCGTAACCGCGATCTGGCAACCGCACTGGA
ACAGCTGCTGCAGGCATATCCGCGCGATATGGAAAAGGAAAAGACAATGCTGGTCCTGGCA
CTGCTGCTGGCAAAGAAGGTGGCAAGTCATACACCTAGCCTGCTGCGCGATGTTTTCCATA
CCACCGTTAATTTTATTAACCAGAACCTGCGCACGTATGTTCGTAGCCTGGCTCGTAATGGTA
TGGATATGAGCCTGCTGAACGTTCCGGCGGGTAAAGACCTGCCGGAAGACATCTACGTGGT
TATCGAAATCCCAGCAAACGCTGATCCGATCAAATACGAGATCGACAAAGAAAGCGGTGCG
CTGTTCGTAGATCGTTTCATGTCCACCGCGATGTTCTATCCGTGCAACTACGGTTACATCAAC
CACACCTTGTCTCTGGACGGTGATCCGGTTGATGTGCTGGTTCCGACGCCGTATCCGTTGCA
GCCGGGTTCCGTTATCCGTTGTCGTCCGGTTGGTGTGCTGAAGATGACTGATGAAGCTGGT
GAAGACGCTAAACTGATCGCTGTTCCGCACACCAAACTGTCTAAAGAATACGACCACATCA
AAGACGTTAACGATCTGCCGGAACTGCTGAAAGCGCAGATCGCTCACTTCTTCGAACACTA
CAAAGATCTGGAGAAAGGTAAGTGGGTTAAAGTTGAAGGCTGGGAGAACGCAGAAGCTG
CTAAAGCAGAAATCGTTGCGTCTTTCGAACGTGCTAAGAACAAG

(8) sfGFP-BID-*Ni*CAR

ATGCGTAAAGGCGAAGAGCTGTTCACTGGTGTCGTCCCTATTCTGGTGGAACTGGATGGTG
ATGTCAACGGTCATAAGTTTTCCGTGCGTGGCGAGGGTGAAGGTGACGCAACTAATGGTAA
ACTGACGCTGAAGTTCATCTGTACTACTGGTAAACTGCCGGTACCTTGGCCGACTCTGGTAA
CGACGCTGACTTATGGTGTTCAGTGCTTTGCTCGTTATCCGGACCATATGAAGCAGCATGAC
TTCTTCAAGTCCGCCATGCCGGAAGGCTATGTGCAGGAACGCACGATTTCCTTTAAGGATG
ACGGCACGTACAAAACGCGTGCGGAAGTGAAATTTGAAGGCGATACCCTGGTAAACCGCA
TTGAGCTGAAAGGCATTGACTTTAAAGAAGACGGCAATATCCTGGGCCATAAGCTGGAATA
CAATTTTAACAGCCACAATGTTTACATCACCGCCGATAAACAAAAAAATGGCATTAAAGCG
AATTTTAAAATTCGCCACAACGTGGAGGATGGCAGCGTGCAGCTGGCTGATCACTACCAGC
AAAACACTCCAATCGGTGATGGTCCTGTTCTGCTGCCAGACAATCACTATCTGAGCACGCA
AAGCGTTCTGTCTAAAGATCCGAACGAGAAACGCGATCATATGGTTCTGCTGGAGTTCGTA
ACCGCAGCGGGCATCACGCATGGTATGGATGAACTGTACAAAATGGATTGTGAAGTTAACA
ACGGTAGCAGCCTGCGTGATGAATGTATTACCAATCTGCTGGTTTTCGGTTTTCTGCAGAGC
TGTAGCGATAATAGCTTTCGTCGTGAACTGGATGCGCTGGGTCATGAACTGCCGGTGCTGGC
ACCTCAGTGGGAAGGTTATGATGAACTGCAGACCGATGGTAATCGTAGCAGCCATTCTCGT
CTGGGTCGTATTGAAGCAGATTCTGAAAGCCAGGAAGATATTATTCGTAATATTGCACGTCA

TCTGGCACAGGTGGGTGATAGCATGGATCGTAGTATCCCGCCGGGTCTGGTTAATGGTCTGG
CACTGCAGCTGCGTAATACCAGCCGTAGCGAAGAAGATCGTAACCGCGATCTGGCAACCGC
ACTGGAACAGCTGCTGCAGGCATATCCGCGCGATATGGAAAAGGAAAAGACAATGCTGGTC
CTGGCACTGCTGCTGGCAAAGAAGGTGGCAAGTCATACACCTAGCCTGCTGCGCGATGTTT
TCCATACCACCGTTAATTTTATTAACCAGAACCTGCGCACGTATGTTCGTAGCCTGGCTCGTA
ATGGTATGGATATGGCTGTTGATTCCCCTGACGAACGTCTGCAACGTCGCATTGCGCAGCTG
TTCGCAGAAGACGAACAGGTGAAGGCAGCTCGTCCGCTGGAAGCGGTGAGCGCGGCAGT
TAGCGCTCCAGGTATGCGCTTGGCACAGATTGCGGCTACTGTCATGGCGGGTTACGCAGATC
GTCCGGCTGCGGGTCAACGCGCATTCGAACTGAACACCGACGATGCCACTGGTCGCACCTC
TCTCCGTCTGCTGCCGCGTTTCGAAACCATCACCTACCGTGAACTGTGGCAGCGTGTGGGT
GAAGTTGCTGCTGCATGGCACCATGATCCGGAGAATCCGCTCCGTGCAGGTGACTTTGTCG
CACTGCTGGGCTTCACCAGCATCGACTATGCGACCTTGGATTTGGCAGACATCCACCTGGG
TGCAGTAACCGTTCCGCTGCAGGCGTCTGCGGCCGTGAGCCAGCTGATCGCCATTCTGACT
GAGACCAGTCCACGTCTGTTGGCATCCACTCCGGAACACCTGGATGCAGCTGTAGAATGTC
TGCTGGCAGGTACTACTCCGGAACGTTTGGTAGTATTCGACTACCATCCGGAAGACGACGA
TCAGCGTGCTGCATTCGAGAGCGCACGTCGCCGCTTGGCAGATGCGGGTTCTTCCGTCATC
GTGGAAACTCTTGATGCCGTTCGTGCTCGTGGCCGTGATTTGCCGGCAGCACCGCTGTTTG
TTCCAGATACCGATGATGATCCGCTGGCTTTGCTGATCTACACCAGCGGTAGCACTGGTACG
CCGAAAGGTGCGATGTACACTAACCGCCTGGCAGCAACTATGTGGCAGGGTAACTCCATGC
TGCAGGGCAACTCTCAACGTGTGGGTATCAACCTGAACTACATGCCGATGTCTCATATCGCT
GGTCGTATCAGCTTGTTCGGTGTTCTGGCTCGTGGTGGCACTGCATACTTTGCTGCAAAGAG
CGACATGTCTACCTTGTTCGAAGACATCGGCTTGGTGCGTCCAACCGAAATCTTCTTTGTTC
CACGTGTCTGCGACATGGTCTTCCAACGCTATCAGAGCGAACTGGATCGCCGTTCTGTAGCT
GGTGCGGATTTGGACACTCTGGACCGTGAAGTGAAGGCCGACCTGCGTCAGAACTACCTG
GGTGGTCGCTTTCTGGTAGCAGTTGTTGGTTCCGCTCCACTGGCTGCTGAAATGAAGACGT
TCATGGAGTCCGTTCTGGACCTGCCACTCCACGACGGCTATGGTTCTACTGAAGCCGGTGC
TAGTGTTCTGCTGGACAACCAGATCCAACGTCCACCAGTTCTGGACTACAAACTGGTTGAC
GTTCCAGAACTGGGCTATTTCCGTACTGATCGTCCGCATCCACGTGGTGAACTGTTGCTGAA
AGCTGAGACCACTATCCCTGGCTACTACAAACGTCCGGAAGTAACCGCTGAAATCTTCGAT
GAAGATGGCTTCTACAAGACCGGCGACATCGTAGCAGAGCTGGAACATGACCGTTTGGTTT
ATGTGGATCGTCGTAATAACGTGCTGAAACTGAGCCAGGGCGAGTTCGTGACCGTTGCGCA
CTTGGAAGCTGTCTTCGCGTCTTCTCCACTGATCCGTCAAATCTTCATCTACGGCAGCTCCG
AACGTAGCTACCTGCTGGCGGTTATCGTTCCAACTGACGATGCACTGCGTGGTCGTGACAC
TGCCACCTTGAAGTCTGCTCTGGCAGAATCCATCCAGCGCATCGCGAAAGACGCGAACTTG
CAGCCATACGAAATCCCACGTGACTTTCTGATCGAAACCGAACCATTTACGATCGCTAACGG
TCTGCTGAGCGGTATTGCTAAGCTGTTGCGTCCGAACCTGAAAGAACGTTATGGTGCTCAG
CTGGAACAGATGTACACTGACCTGGCAACTGGTCAAGCGGATGAACTGCTGGCACTGCGT
CGCGAAGCGGCTGATCTGCCGGTGTTGGAAACTGTTAGCCGTGCAGCCAAAGCGATGCTG
GGTGTTGCATCCGCTGACATGCGTCCAGATGCTCACTTCACCGATCTGGGTGGTGACTCTCT
GAGCGCACTGTCTTTCAGCAACCTGCTCCACGAAATCTTTGGCGTCGAAGTTCCGGTAGGT
GTAGTTGTAAGTCCAGCAAACGAACTGCGCGATCTGGCAAACTACATTGAGGCGGAACGTA
ACAGCGGCGCTAAACGCCCAACGTTTACCTCTGTACACGGCGGTGGTAGCGAAATCCGTGC
TGCAGACCTGACTCTGGACAAGTTCATCGACGCGCGTACTTTGGCAGCAGCGGATTCCATT
CCGCACGCACCAGTACCGGCTCAGACCGTGCTGCTGACTGGTGCGAACGGTTACCTGGGTC

GTTTCTTGTGCTTGGAATGGCTGGAACGTCTGGATAAGACTGGTGGCACTCTGATCTGTGTG
GTACGTGGCTCCGATGCTGCTGCTGCACGTAAGCGTCTGGACTCTGCTTTCGACAGCGGCG
ATCCGGGTCTGTTGGAACACTATCAGCAGCTCGCTGCTCGTACCTTGGAAGTTCTGGCTGGT
GACATCGGTGATCCGAACTTGGGCCTGGATGACGCTACTTGGCAACGTCTGGCGGAAACCG
TTGATCTGATCGTGCACCCGGCAGCTCTCGTTAACCATGTGCTGCCATACACGCAACTGTTC
GGTCCGAACGTTGTTGGTACGGCGGAAATCGTGCGTCTGGCAATCACCGCGCGTCGTAAGC
CGGTAACCTACCTGTCTACCGTGGGTGTAGCAGATCAAGTGGACCCTGCTGAGTACCAAGA
AGATAGCGATGTGCGTGAGATGTCTGCGGTACGCGTTGTTCGCGAATCTTACGCTAACGGTT
ATGGTAACAGCAAATGGGCAGGTGAAGTTCTGCTGCGCGAAGCTCATGACCTGTGTGGTTT
GCCGGTTGCAGTATTCCGCAGCGACATGATCTTGGCGCACAGCCGTTACGCAGGCCAGCTG
AACGTTCAAGATGTGTTCACGCGTCTGATCTTGAGCCTGGTTGCTACTGGCATCGCACCATA
TAGCTTCTATCGTACCGATGCTGATGGTAACCGTCAGCGCGCTCATTACGACGGCCTGCCAG
CCGACTTCACCGCAGCTGCTATCACCGCTCTGGGTATTCAGGCTACTGAGGGCTTTCGTACC
TACGATGTGCTGAACCCGTATGATGATGGCATCAGCCTGGATGAGTTTGTGGACTGGCTTGT
TGAATCTGGTCATCCGATCCAGCGTATCACCGATTACAGCGACTGGTTCCACCGCTTTGAAA
CTGCGATCCGCGCTTTGCCAGAGAAACAGCGTCAAGCGTCTGTGTTGCCACTGCTGGACGC
TTACCGCAATCCGTGTCCAGCTGTTCGTGGTGCAATCTTGCCGGCTAAAGAATTTCAGGCTG
CGGTTCAGACCGCGAAGATCGGTCCGGAACAAGACATTCCGCATCTGAGCGCGCCGCTGAT
CGACAAGTACGTCTCCGACTTGGAACTGCTCCAGCTGCTGTAA

(9) EYFP-BID-*Bp*GDH

ATGGTGAGCAAAGGCGAAGAACTGTTTACCGGTGTGGTTCCGATTCTGGTGGAACTGGATG
GCGATGTTAATGGTCATAAATTTAGCGTTAGTGGTGAAGGTGAAGGTGATGCAACCTATGGC
AAACTGACCCTGAAATTTATTTGCACCACCGGCAAACTGCCGGTGCCGTGGCCGACCCTGG
TGACAACCTTTGGTTATGGTCTGCAGTGCTTTGCACGTTATCCGGATCATATGAAACAGCAT
GATTTTTTTAAGAGCGCAATGCCGGAAGGCTATGTTCAGGAACGCACCATTTTTTTTAAAGA
TGATGGTAATTACAAGACCCGCGCCGAAGTGAAATTTGAAGGTGATACCCTGGTTAATCGTA
TTGAACTGAAAGGTATTGATTTCAAAGAGGATGGTAATATTCTGGGTCATAAACTGGAATAT
AACTATAATAGCCACAACGTTTACATCATGGCAGATAAACAGAAAAATGGTATTAAGGTTAA
CTTCAAGATCCGTCATAATATTGAAGATGGCAGCGTTCAGCTGGCCGATCATTATCAGCAGA
ATACCCCGATTGGTGATGGTCCGGTGCTGCTGCCGGATAATCATTATCTGAGCTATCAGAGTG
CACTGAGCAAAGATCCGAATGAAAAACGTGATCATATGGTTCTGCTGGAATTTGTTACCGCA
GCCGGCATTACCCTGGGCATGGATGAACTGTATAAAATGGATTGTGAAGTTAACAACGGTAG
CAGCCTGCGTGATGAATGTATTACCAATCTGCTGGTTTTCGGTTTTCTGCAGAGCTGTAGCG
ATAATAGCTTTCGTCGTGAACTGGATGCGCTGGGTCATGAACTGCCGGTGCTGGCACCTCA
GTGGGAAGGTTATGATGAACTGCAGACCGATGGTAATCGTAGCAGCCATTCTCGTCTGGGT
CGTATTGAAGCAGATTCTGAAAGCCAGGAAGATATTATTCGTAATATTGCACGTCATCTGGC
ACAGGTGGGTGATAGCATGGATCGTAGTATCCCGCCGGGTCTGGTTAATGGTCTGGCACTGC
AGCTGCGTAATACCAGCCGTAGCGAAGAAGATCGTAACCGCGATCTGGCAACCGCACTGGA
ACAGCTGCTGCAGGCATATCCGCGCGATATGGAAAAGGAAAAGACAATGCTGGTCCTGGCA
CTGCTGCTGGCAAAGAAGGTGGCAAGTCATACACCTAGCCTGCTGCGCGATGTTTTCCATA
CCACCGTTAATTTTATTAACCAGAACCTGCGCACGTATGTTCGTAGCCTGGCTCGTAATGGTA
TGGATATGTACAGTGATCTGGAAGGTAAAGTGGTGGTGATTACCGGCAGCGCCAGTGGTCT
GGGCCGCGCAATGGGCGTTCGCTTTGCCCGCGAAAAGCAAAAGTGGTGATTAATTATCGT
AGTCGTGAAAGCGAAGCCAATGATGTGCTGGAAGAAATTAAAAAAGTGGGTGGTGAAGCA

ATTGCAGTTAAAGGCGATGTGACCGTTGAAAGTGATGTTGTTAATCTGATTCAGAGTGCCGT
GAAAGAATTTGGTACCCTGGATGTGATGATTAATAATGCAGGTATTGAAAACGCAGTTCCGA
GCCATGAAATGCCGCTGGAAGATTGGAATCGTGTTATTAATACCAATCTGACCGGCGCATTT
CTGGGCAGTCGCGAAGCCATTAAATATTTTGTTGAACATGACATCAAGGGCAGCGTTATTAA
TATGAGCAGCGTTCATGAAAAAATCCCGTGGCCGCTGTTTGTTCATTATGCCGCAAGCAAAG
GCGGTATGAAACTGATGACCGAAACCCTGGCCATGGAATATGCACCGAAAGGTATTCGCGT
GAATAATATTGGCCCGGGTGCAATTAATACCCCGATTAATGCAGAAAAATTCGCCGATCCGA
AAAAACGCGCCGATGTGGAAAGCATGATTCCGATGGGCTATATTGGCAAACCGGAAGAAAT
TGCAGCCGTGGCAACCTGGCTGGCCAGCAGCGAAGCCAGTTATGTTACCGGCATTACCCTG
TTTGCAGATGGCGGCATGACCCTGTATCCGAGTTTTCAGGCCGGTCGCGGTTAA

(10) mCherry-BID-PPK12

ATGGCAAGCATGACGGGTGGTCAGCAGATGGGTCGTGATCTGTATGATGATGATGATAAAG
ATCCGGCGACGATGGTTAGCAAAGGTGAAGAAGATAATATGGCAATTATCAAAGAATTTATG
CGCTTTAAAGTGCATATGGAAGGTAGTGTTAATGGTCATGAATTTGAAATTGAAGGTGAAGG
TGAAGGCCGTCCGTATGAAGGTACCCAGACAGCGAAACTGAAAGTGACCAAAGGTGGCCC
GCTGCCGTTTGCGTGGGATATTCTGAGCCCGCAGTTTATGTATGGTAGCAAAGCATATGTGA
AACATCCGGCAGATATTCCGGATTATCTGAAACTGTCCTTTCCAGAAGGTTTTAAATGGGAA
CGTGTTATGAATTTTGAAGATGGTGGTGTTGTTACGGTTACCCAGGATAGCAGTCTGCAGGA
TGGTGAATTTATTTATAAAGTGAAACTGCGCGGTACCAATTTTCCGAGCGATGGTCCTGTTAT
GCAGAAAAAGACCATGGGTTGGGAAGCAAGCAGCGAACGTATGTATCCAGAAGATGGTGC
ACTGAAAGGTGAAATTAAACAGCGTCTGAAACTGAAAGATGGTGGTCATTATGATGCCGAA
GTGAAAACAACCTATAAAGCAAAAAAACCGGTTCAGCTGCCGGGTGCATATAATGTTAATAT
TAAACTGGATATTACCAGCCATAATGAAGATTATACCATTGTTGAACAGTATGAACGTGCAG
AAGGTCGTCATAGCACCGGTGGTATGGATGAACTGTATAAAATGGATTGTGAAGTTAACAAC
GGTAGCAGCCTGCGTGATGAATGTATTACCAATCTGCTGGTTTTCGGTTTTCTGCAGAGCTG
TAGCGATAATAGCTTTCGTCGTGAACTGGATGCGCTGGGTCATGAACTGCCGGTGCTGGCA
CCTCAGTGGGAAGGTTATGATGAACTGCAGACCGATGGTAATCGTAGCAGCCATTCTCGTCT
GGGTCGTATTGAAGCAGATTCTGAAAGCCAGGAAGATATTATTCGTAATATTGCACGTCATC
TGGCACAGGTGGGTGATAGCATGGATCGTAGTATCCCGCCGGGTCTGGTTAATGGTCTGGCA
CTGCAGCTGCGTAATACCAGCCGTAGCGAAGAAGATCGTAACCGCGATCTGGCAACCGCAC
TGGAACAGCTGCTGCAGGCATATCCGCGCGATATGGAAAAGGAAAAGACAATGCTGGTCCT
GGCACTGCTGCTGGCAAAGAAGGTGGCAAGTCATACACCTAGCCTGCTGCGCGATGTTTTC
CATACCACCGTTAATTTTATTAACCAGAACCTGCGCACGTATGTTCGTAGCCTGGCTCGTAAT
GGTATGGATATGATCAACATCTACAAGATCGATAAACTGAACAACTTCAACCTGAACAACCA
CAAGACCGACGATTACAGCCTGTGCAAAGACAAAGATACCGCGCTGGAACTGACTCAGAA
GAACATCCAGAAGATCTACGACTACCAGCAGAAACTGTACGCGGAGAAGAAAGAAGGTCT
GATCATCGCGTTCCAGGCGATGGATGCGGCTGGTAAAGACGGTACTATCCGTGAAGTGTTG
AAAGCTCTGGCTCCGCAGGGTGTTCACGAGAAACCATTCAAATCTCCATCTTCCACCGAAC
TGGCGCACGACTACCTGTGGCGTGTTCACAACGCAGTACCGGAGAAAGGTGAAATCACCA
TCTTCAACCGTTCTCACTACGAAGATGTTCTGATCGGCAAAGTTAAAGAACTGTACAAATTC
CAGAACAAAGCTGACCGTATCGATGAGAACACCGTTGTGGACAACCGCTACGAAGATATCC
GTAACTTCGAGAAATACCTGTACAACAACTCTGTTCGTATCATCAAGATCTTTCTGAACGTT
TCTAAAAAAGAACAGGCTGAACGCTTTCTGAGCCGTATCGAAGAACCAGAGAAGAACTGG

AAGTTCTCTGACTCCGACTTCGAAGAACGTGTATACTGGGACAAATACCAGCAGGCGTTCG
AAGACGCGATCAACGCGACCTCCACCAAAGACTGTCCGTGGTATGTTGTTCCGGCTGATCG
TAAATGGTACATGCGTTACGTGGTGTCTGAAATCGTAGTTAAGACCTTGGAAGAAATGAATC
CGAAATATCCGACCGTTACCAAAGAAACTCTGGAACGTTTCGAAGGTTACCGTACCAAACT
GCTGGAAGAATACAACTACGATCTGGACACCATCCGTCCGATCGAGAAG

(11) BID-*Asp*RedAm

ATGGATTGTGAAGTTAACAACGGTAGCAGCCTGCGTGATGAATGTATTACCAATCTGCTGGT
TTTCGGTTTTCTGCAGAGCTGTAGCGATAATAGCTTTCGTCGTGAACTGGATGCGCTGGGTC
ATGAACTGCCGGTGCTGGCACCTCAGTGGGAAGGTTATGATGAACTGCAGACCGATGGTAA
TCGTAGCAGCCATTCTCGTCTGGGTCGTATTGAAGCAGATTCTGAAAGCCAGGAAGATATTA
TTCGTAATATTGCACGTCATCTGGCACAGGTGGGTGATAGCATGGATCGTAGTATCCCGCCG
GGTCTGGTTAATGGTCTGGCACTGCAGCTGCGTAATACCAGCCGTAGCGAAGAAGATCGTA
ACCGCGATCTGGCAACCGCACTGGAACAGCTGCTGCAGGCATATCCGCGCGATATGGAAAA
GGAAAAGACAATGCTGGTCCTGGCACTGCTGCTGGCAAAGAAGGTGGCAAGTCATACACC
TAGCCTGCTGCGCGATGTTTTCCATACCACCGTTAATTTTATTAACCAGAACCTGCGCACGTA
TGTTCGTAGCCTGGCTCGTAATGGTATGGATGGAGGATCAGGAGGATCAGGAGGATCAATG
AGCAAACACATCGGCATCTTCGGTCTGGGTGCTATGGGTACCGCACTGGCGGCGAAATACC
TCGAACATGGTTACAAGACTAGCGTATGGAACCGTACCACCGCTAAAGCGATTCCGCTGGT
TGAACAGGGCGCTAAACTGGCTTCTACCATCTCCGAAGGTGTTAACGCGAACGACCTGATC
ATCATCTGCCTGCTGAACAACCAGGTTGTTGAAGATGCGTTGCGTGATGCTCTGCAGACTCT
GCCGTCTAAGACTATCGTTAACCTGACCAACGGTACTCCGAACCAGGCGCGTAAACTGGCA
GATTTCGTGACTAGCCACGGTGCGCGCTACATCCACGGTGGTATCATGGCAGTTCCGACCAT
GATCGGTTCTCCGCACGCAGTCTTGCTGTACTCCGGTGAATCTCTGGAACTGTTCCAGTCTA
TCGAAAGCCACCTGAGCCTTCTGGGTATGTCCAAATACCTGGGTACCGACGCTGGTTCCGC
TTCTCTGCACGATTTGGCGCTGCTGTCTGGTATGTATGGTCTGTTCTCCGGTTTTCTGCACGC
TGTTGCACTGATCAAATCCGGTCAAGACACCTCTACCACCGCAACTGGTCTGCTGCCGCTG
CTGACTCCGTGGCTGAGCGCTATGACTGGTTACCTGTCTAGCATCGCGAAACAGATCGACG
ATGGCGACTACGCTACTCAGGGTTCCAACCTGGGCATGCAGCTCGCAGGTGTTGAGAACAT
CATCCGTGCGGGTGAAGAACAGCGTGTTTCTTCTCAGATGATCTTGCCGATCAAAGCACTG
ATCGAACAGGCGGTTGGCGAAGGTCATGGTGGTGAAGACCTGTCCGCTCTGATCGAATACT
TCAAAGTTGGCAAGAACGTTGAC

3. Figures and tables

Table S1. Primers used for PCR amplification.

Primer	Sequence
pRSF-MCS1-F	GAATTCGGATCCTGGCTGTG
pRSF-MCS1-R	AAGCTTGCGGCCGCATAATGC
BID-F	CACAGCCAGGATCCGAATTCCATGGATTGTGAAGTTAACAAC
BID-*Ni*CAR-R	GAATCAACAGCCATATCCATACCATTACGAGCCAGG
PopZ-F	AGCAAATGGGTCGCGATGTCTGACCAGTCTCA
PopZ-*Ni*CAR-R	AGGGGAATCAACAGCCATAGCACCACGACCACGAGA
ASR-F	CAGGATCCGAATTCCATGAAAAAAGTATTAGCTCTGG
ASR-*Ni*CAR-R	GGAATCAACAGCCATCGCTGCGGGTTGTGCAGC
UTX-F	TGGGTCGCGGATCCGAATTCGTGCCTAGCGTCTCTCAGCC
UTX-*Ni*CAR-R	GGAATCAACAGCCATGTGAGGGCTGTTAAGGCTGG
*Ni*CAR-BID-F	CCGGAATTCATGGCAAGTAATGATTATAC
*Ni*CAR-PopZ-F	AGGGGAATCAACAGCCATAGCACCACGACCACGAGA
*Ni*CAR-ASR-F	CTGCTGCACAACCCGCAGCGATGGCTGTTGATTCCC
*Ni*CAR-UTX-F	CTTAACAGCCCTCACATGGCTGTTGATTCCCCTGA
*Ni*CAR-R	TGGTGGTGGTGCTCGAGTTACAGCAGCTGGAGCA
pRSF-MCS2-F	AAGGAGATATACATATG
pRSF-MCS2-R	TTTCTTTACCAGACTCGAG
PPTase-F	AAGGAGATATACATATGATGGTTGACATGAAGACT
PPTase-R	TCTTTACCAGACTCGAGGTCGTGCTGACACAGAGT
pET28a-*EcoR*I-F	GAATTCGGATCCGCGACC
pET28a-*Hind*III-R	AAGCTTGCGGCCGCACT
BID-PPK12-R	TTGTAGATGTTGATCATATCCATACCATTACGAGC
BID-*Ec*PPase-R	AACGTTCAGCAGGCTCATATCCATACCATTACGAG
BID-*Bp*GDH-R	TTCCAGATCACTGTACATATCCATACCATTACGAG
PPK-BID-F	GCTCGTAATGGTATGGATATGATCAACATCTACAA
PPK-BID-R	TCCGTCCGATCGAGAAGAAGCTTGCGGCCGCACT
*Ec*PPase-BID-F	CTCGTAATGGTATGGATATGAGCCTGCTGAACGTT
*Ec*PPase-BID-R	AACGTGCTAAGAACAAGAAGCTTGCGGCCGCACTC
*Bp*GDH-BID-F	CTCGTAATGGTATGGATATGTACAGTGATCTGGAA
*Bp*GDH -BID-R	AGGCCGGTCGCGGTTAAAAGCTTGCGGCCGCACTC
BFP-F	GGTCGCGGATCCGAATTCCATATGAGCGAACTGATTA
BFP-R	TTGTTAACTTCACAATCCATCTCGAGATTCAGTTTA
sfGFP-F	AGCCAGGATCCGAATTCCATGCGTAAAGGCGAAGAGC
sfGFP-R	GTTAACTTCACAATCCATTTTGTACAGTTCATCCAT
EYFP-F	CGCGGATCCGAATTCATGGTGAGCAAAGGCGAAG
EYFP-R	AACTTCACAATCCATTTTATACAGTTCATCCATGCCCAGG
mCherry-F	GTCGCGGATCCGAATTCATGGCAAGCATGACGGGTGG
mCherry-R	ATGGATGAACTGTATAAAATGGATTGTGAAGTTAAC
*Asp*RedAm-BID-F	CGTAATGGTATGGATATGAGCAAACACATCGGCATC
*Asp*RedAm-BID-R	TGCGGCCGCAAGCTTGTCAACGTTCTTGCCAACTTTG

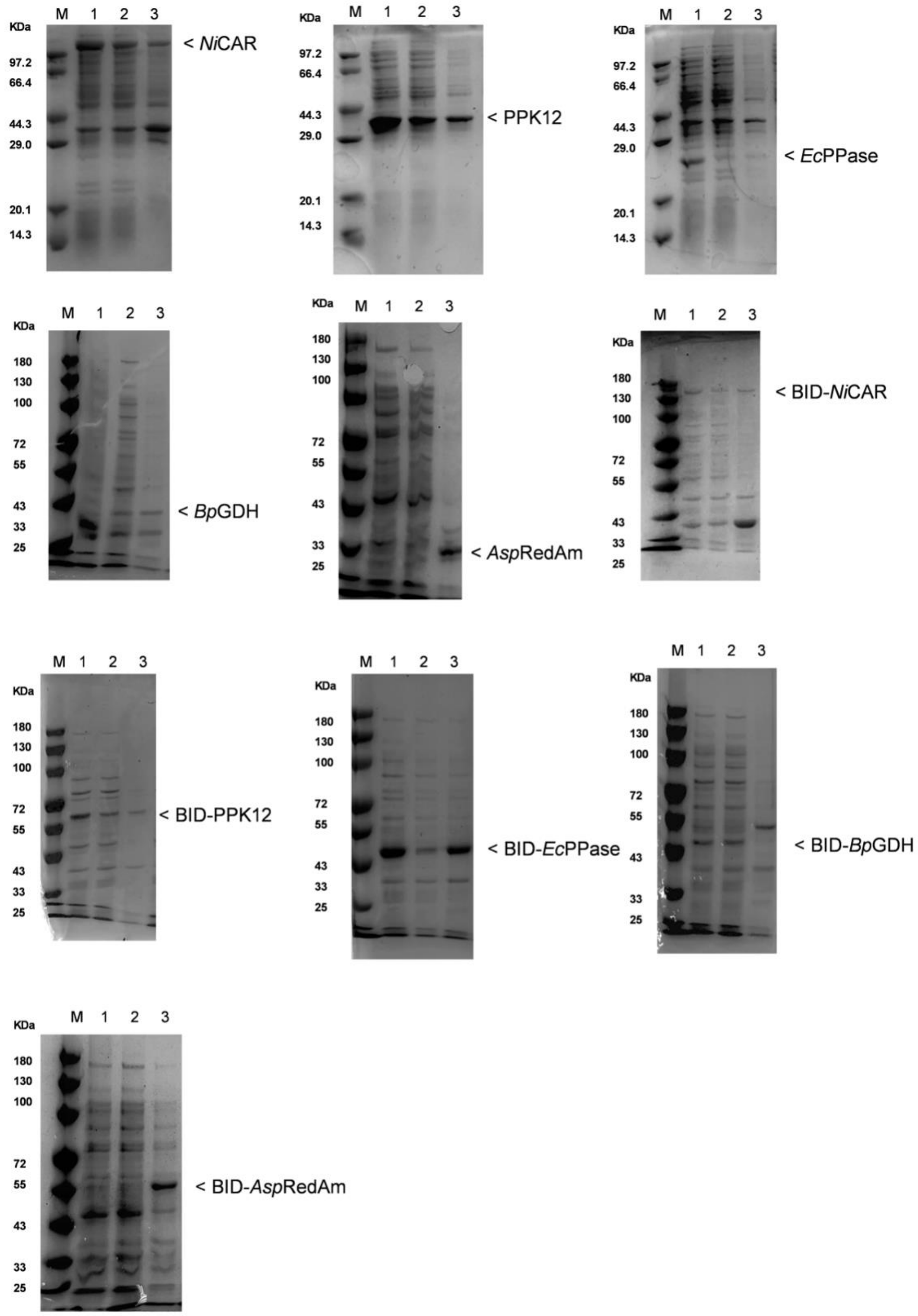

Figure S1. SDS-PAGE of free enzyme and BID-linked fusion enzymes. Lane M: protein marker; lane 1: supernatant of cell lysates; lane 2: Ni-NTA column flow through; lane 3: insoluble protein precipitate.

Table S2. Kinetic parameters of fusion proteins and free enzymes

Entry	Enzyme	K_m (mM)	k_{cat} (min^{-1})	k_{cat}/K_m (min^{-1} mM^{-1})
1	*Ni*CAR[a]	0.45 ± 0.03	235.36 ± 2.27	523.02 ± 35.23
2	BID-*Ni*CAR[a]	1.06 ± 0.03	243.37 ± 2.65	229.59 ± 6.96
3	*Ec*PPase[b]	7.47 ± 1.98	295.15 ± 44.96	39.53 ± 12.13
4	BID-*Ec*PPase[b]	5.77 ± 0.76	102.89 ± 6.41	17.83 ± 2.60
5	*Bp*GDH[c]	0.97 ± 0.05	1472.02 ± 16.53	1512.25 ± 77.51
6	BID-*Bp*GDH[c]	1.51 ± 0.10	269.27 ± 2.52	178.35 ± 11.61
7	PPK12[d]	21.22±2.67	1027.22±81.75	48.41±7.21
8	BID-PPK12[d]	26.55±7.17	1810.20±310.62	68.18±21.82
9	*Asp*RedAm[e]	5.24 ± 0.50	76.64 ± 1.25	14.62 ± 1.43
10	BID-*Asp*RedAm[e]	5.53 ± 0.41	90.86 ± 2.77	16.41 ± 1.30
11	*Ni*CAR+ *Ec*PPase+ *Bp*GDH+ PPK12 (toward substrate **1a**)[f]	3.41 ± 0.12	965.64 ± 31.41	283.18 ± 14.57
12	BID-*Ni*CAR+ BID-*Ec*PPase+ BID-*Bp*GDH+ BID-PPK12+ BID protein (toward substrate **1a**)[g]	0.48 ± 0.03	286.49 ± 0.89	594.69 ± 38.62

a: Benzoic acid **1a** (0.5-20 mM), $MgCl_2$ (10 mM), DTT (1 mM), ATP (1 mM), NADPH (0.2 mM), 0.5 μM enzyme. Reaction was performed in 100 mM MOPS, pH 7.5 at 30°C; b: Activities of pyrophosphatase preparations were assessed using an assay described by Sigma-Aldrich for the characterization of inorganic phosphatase; c: Glucose (1-20 mM), $NADP^+$ (0.2 mM), 0.1 μM enzyme. Reaction was performed in 100 mM MOPS, pH 7.5 at 30°C; d: Activities of PPK preparations were assessed using an assay described in the reference[1]; e: Hexanol (0.5-20 mM), propargylamine (20 mM), NADPH (0.2 mM), 0.5 μM enzyme. Reaction was performed in 100 mM Tris-HCl, pH 9.0 at 30°C; f: Benzoic acid **1a** (1-25 mM), ATP (1 mM), NADPH (0.2 mM), *Ni*CAR (8 μM), PPK12 (5 U/mL), *Ec*PPase (5 U/mL), *Bp*GDH (8 μM), β-D-glucose (100 mM), $MgCl_2$ (25 mM), $PolyP_6$ (8 mM), 100 mM MOPS buffer pH 7.5, at 30 °C, 750 rpm. g: Benzoic acid **1a** (1-25 mM), ATP (1 mM), NADPH (0.2 mM), BID-*Ni*CAR (8 μM), BID-PPK12 (5 U/mL), BID-*Ec*PPase (5 U/mL), BID-*Bp*GDH (8 μM), BID protein (2 μM), β-D-glucose (100 mM), $MgCl_2$ (25 mM), $PolyP_6$ (8 mM), 100 mM MOPS buffer pH 7.5, at 30 °C, 750 rpm.

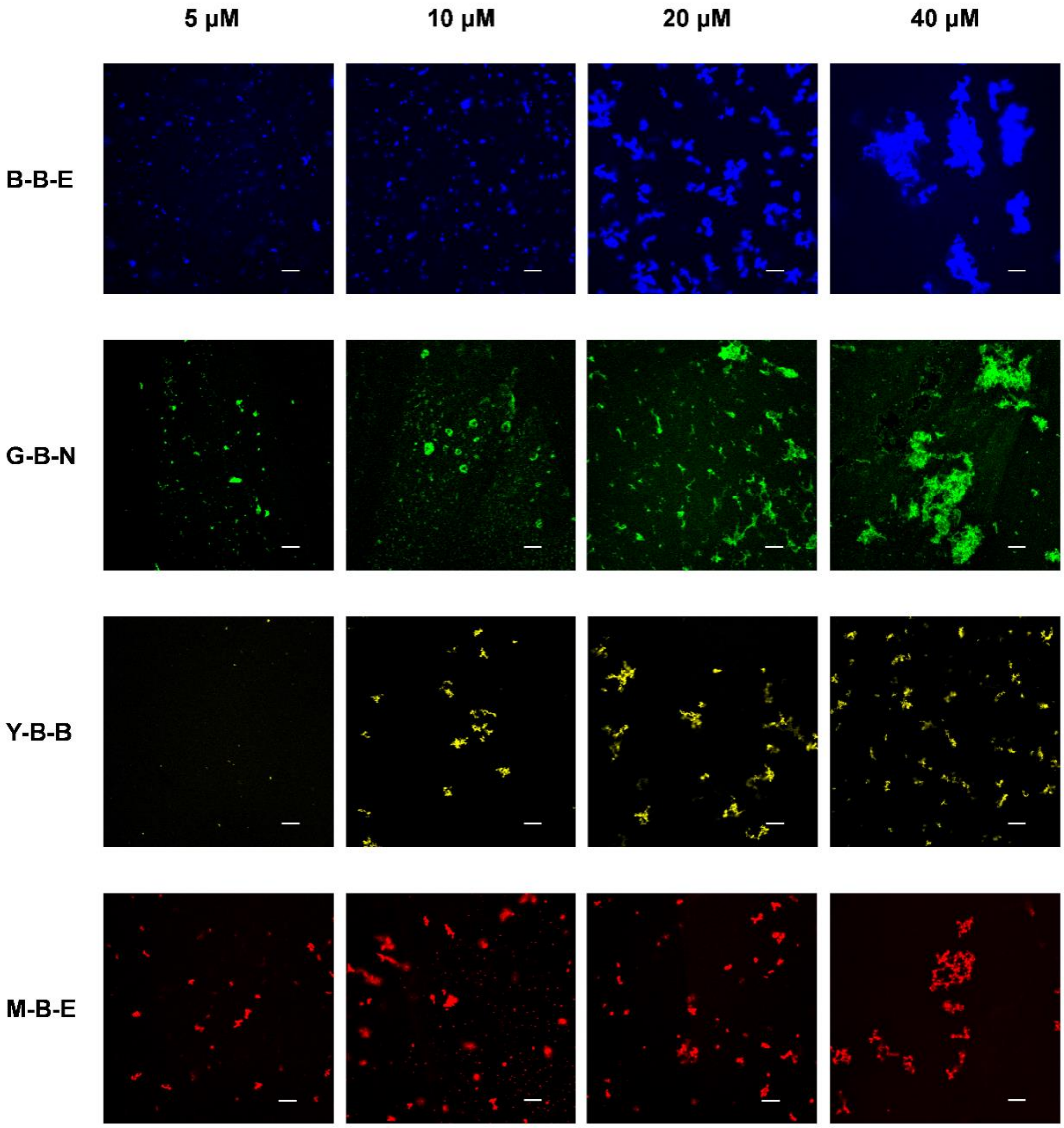

Figure S2. Confocal fluorescence images of four fusion proteins at different concentrations (5, 10, 20, 40 μM). Scale bar, 10 μm.

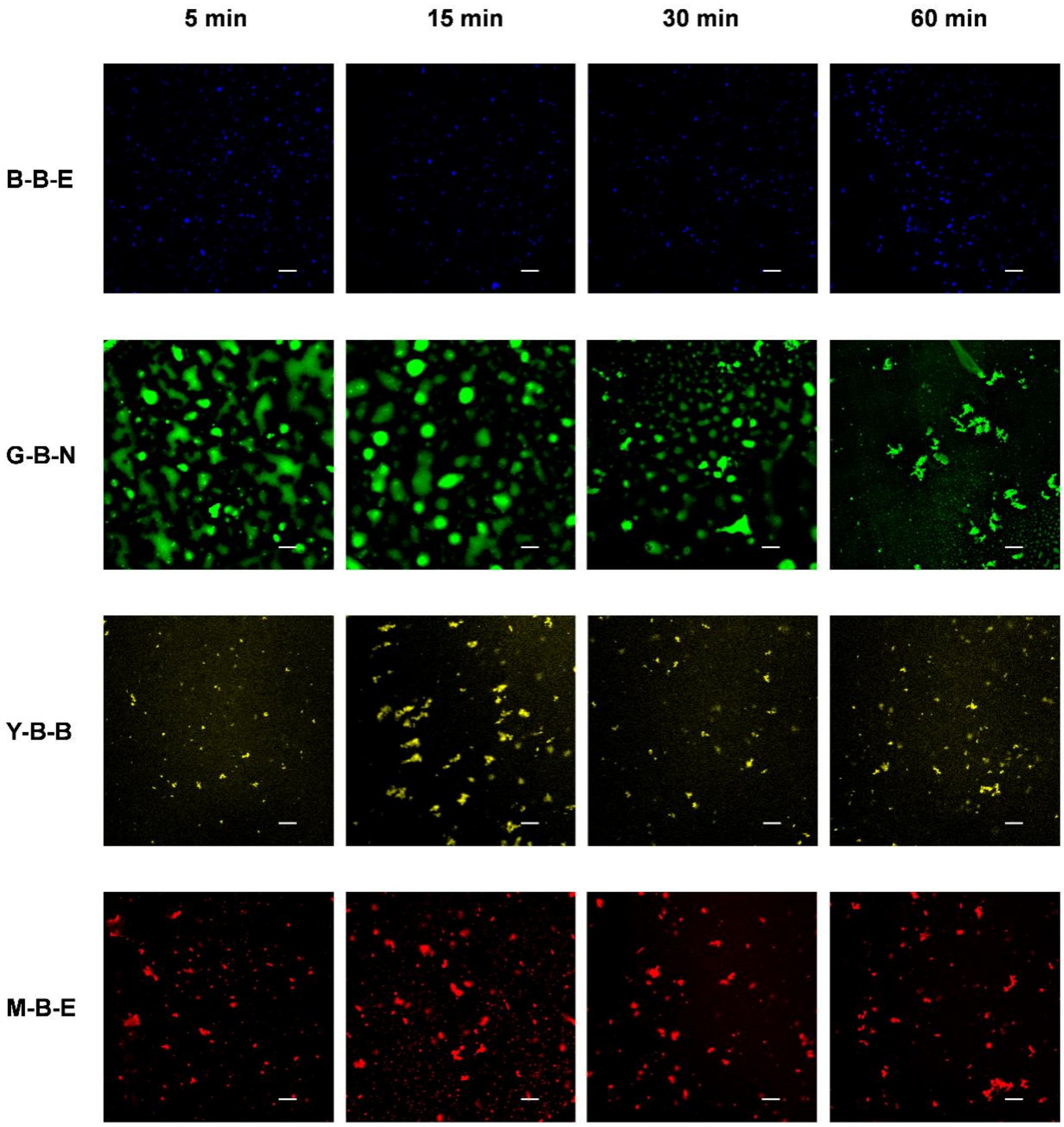

Figure S3. Confocal fluorescence images of four fusion proteins under different incubation time (5, 15, 30, 60 min). Scale bar, 10 μm.

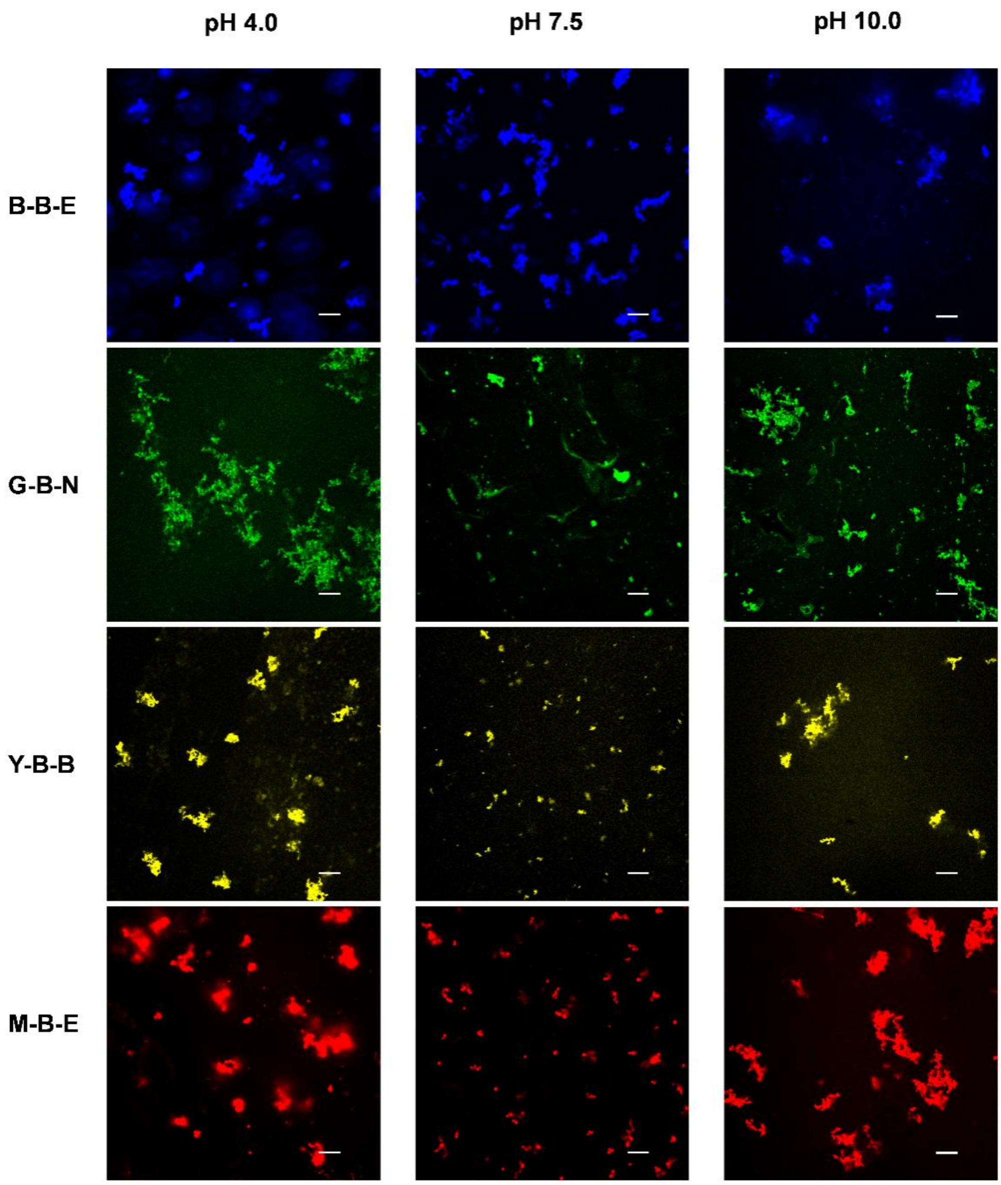

Figure S4. Confocal fluorescence images of four fusion proteins under different pH (4.0, 7.5, 10.0). Scale bar, 10 μm.

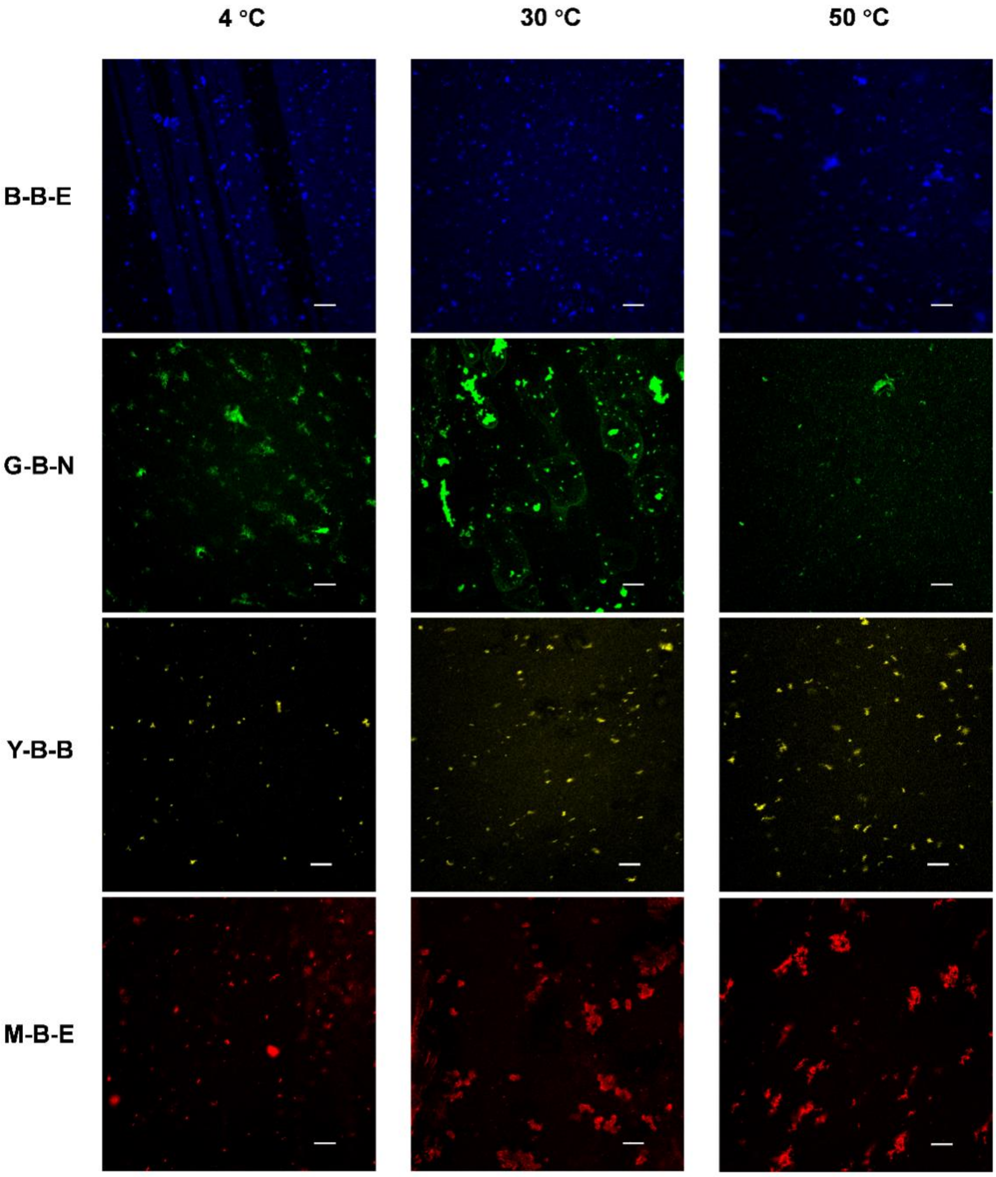

Figure S5. Confocal fluorescence images of four fusion proteins under different incubate temperatures (4, 30, 50 °C). Scale bar, 10 μm.

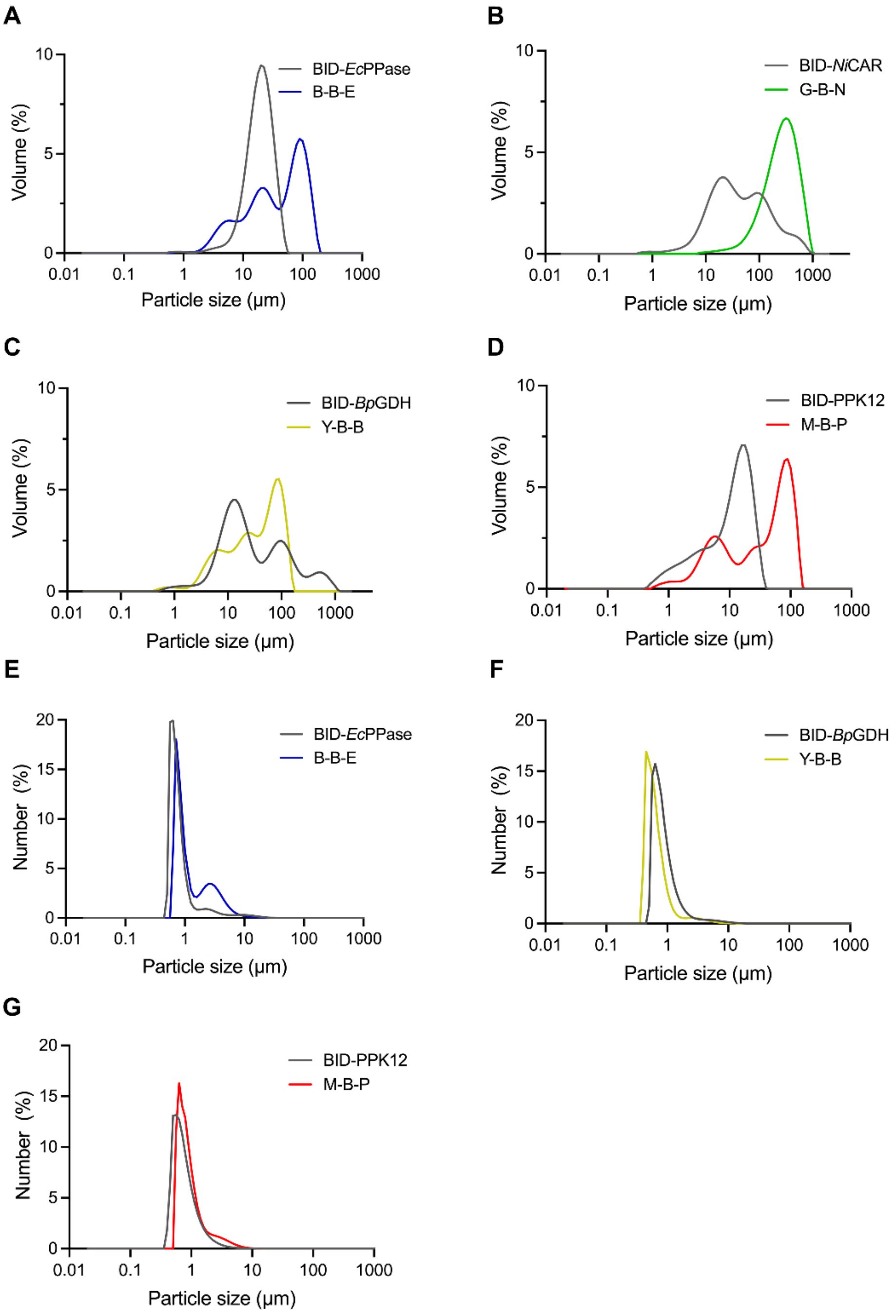

Figure S6. Particle size and size distribution analysis of free enzyme and phase-separated condensates. (A) Analysis of the particle size of BID-*Ec*PPase and BFP-BID-*Ec*PPase with the same concentration of 1 mg/mL. (B) Analysis of the particle size of BID-*Ni*CAR and sfGFP-BID-*Ni*CAR with the same concentration of 1 mg/mL. (C) Analysis of the particle size of BID-*Bp*GDH and EYFP-BID-*Bp*GDH with the same concentration of 1 mg/mL. (D) Analysis of the particle size of BID-PPK12 and mCherry-BID-PPK12 with the same concentration of 1 mg/mL. (E) Size distribution of the particle size of BID-*Ec*PPase and BFP-BID-*Ec*PPase with the same concentration of 1 mg/mL. (F) Size distribution of the particle size of BID-*Bp*GDH and EYFP-BID-*Bp*GDH with the same concentration of 1 mg/mL. (G) Size distribution of condensates generated by 1 mg/mL BID-PPK12 and 1 mg/mL mCherry-BID-PPK12.

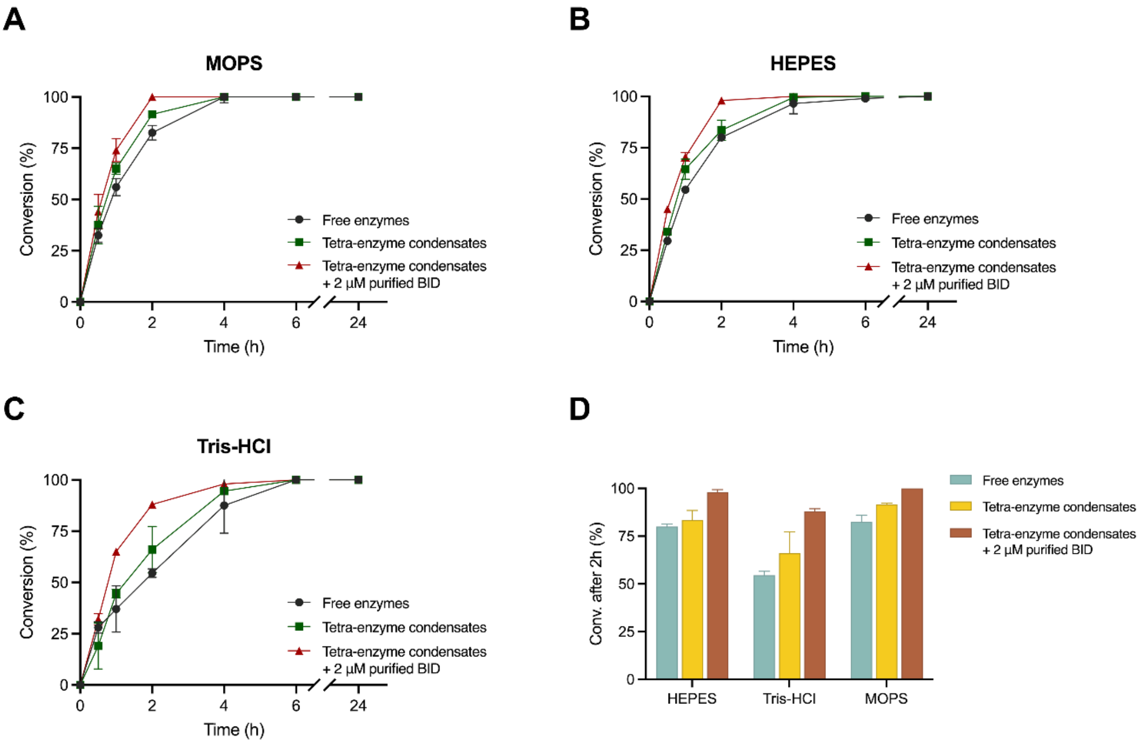

Figure S7. *In vitro* reduction of **1a** under the free enzyme system and the tetra-enzyme systems in the presence of different buffers. (A) MOPS. (B) HEPES. (C) Tris-HCl**.** (D) Conversions of substrate **1a** under free enzyme system and tetra-enzyme condensate systems after 2 h.

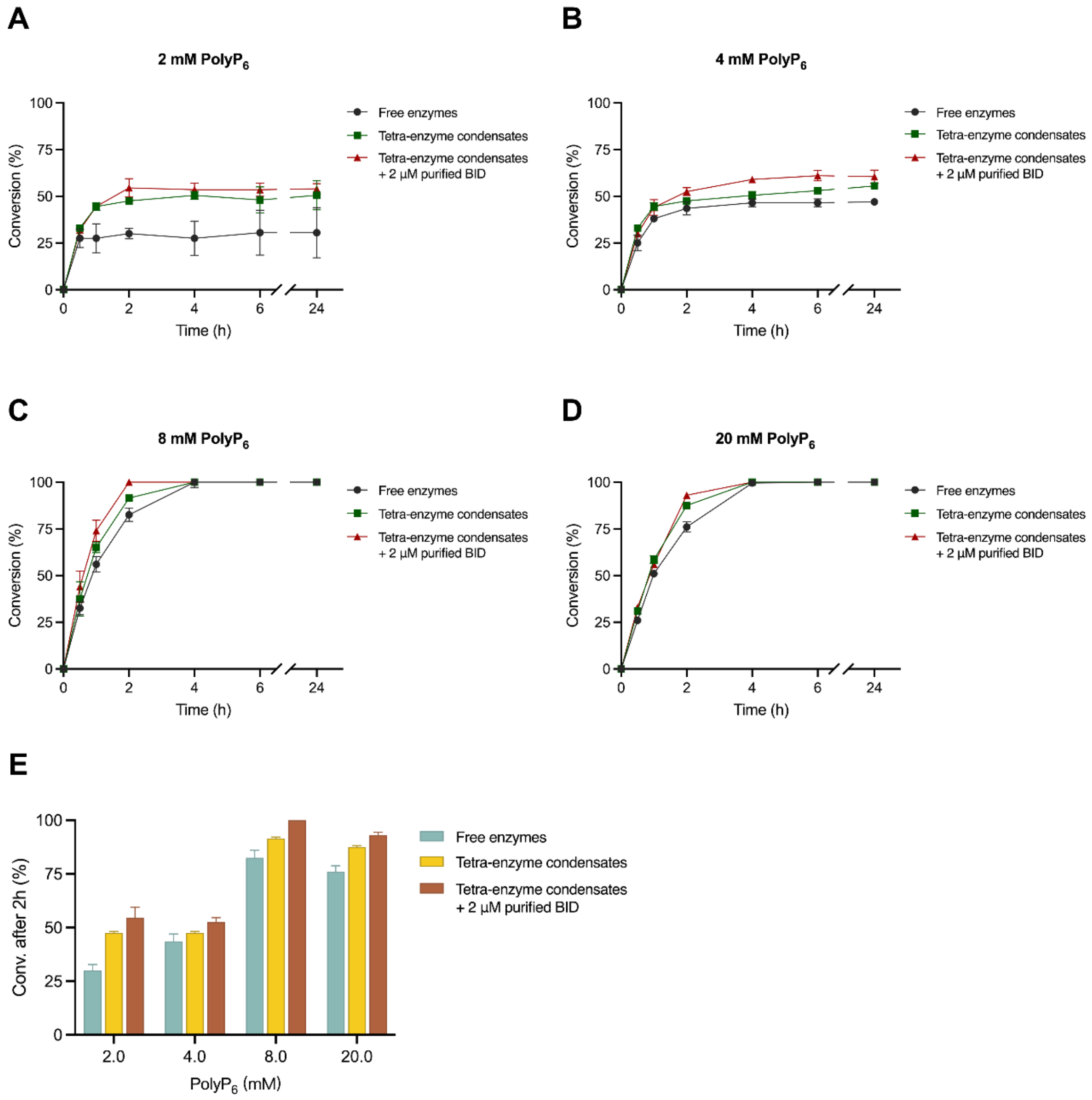

Figure S8. *In vitro* reduction of **1a** under the free enzyme system and the tetra-enzyme systems in the presence of different concentration of $PolyP_6$. (A) 2 mM $PolyP_6$. (B) 4 mM $PolyP_6$. (C) 8 mM $PolyP_6$. (D) 20 mM $PolyP_6$. (E) Conversions of substrate **1a** under free enzyme system and tetra-enzyme condensate systems after 2 h.

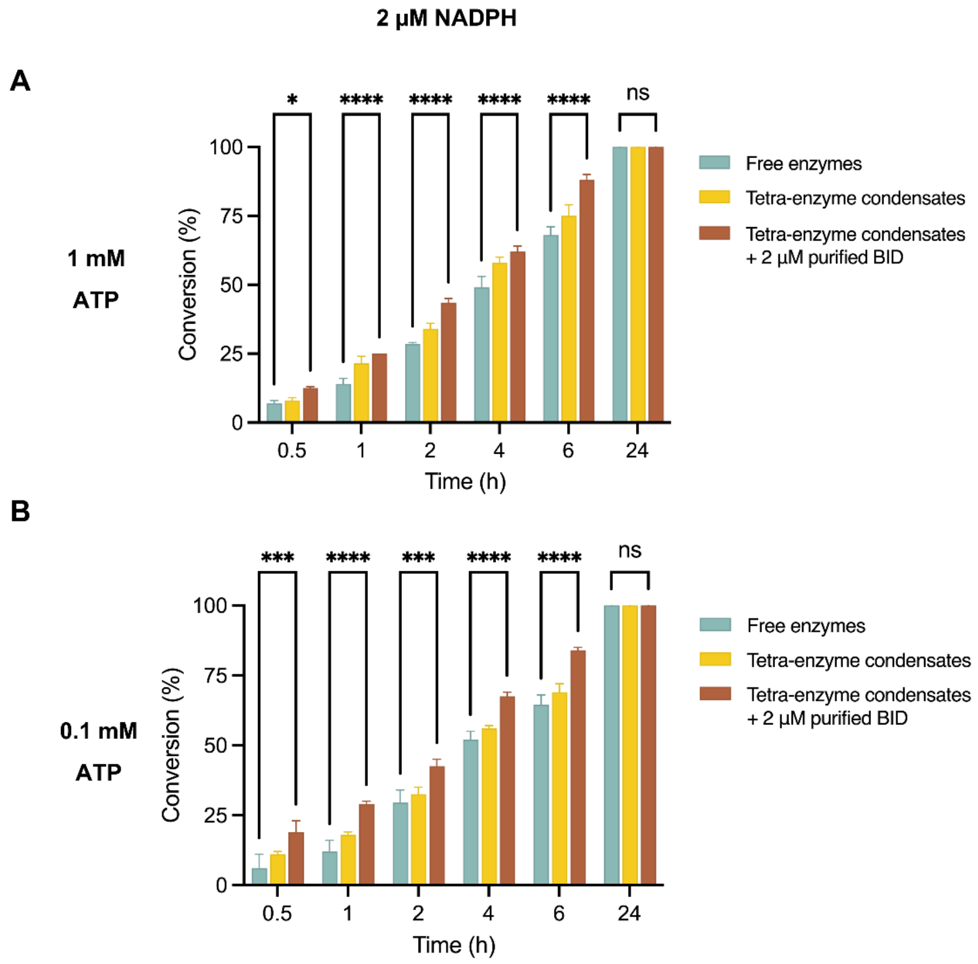

Figure S9. The conversion of 1a over time in the free enzyme system and the tetra-enzyme condensate systems. (A) Reaction conditions: substrate 1a (15 mM), ATP (1 mM), $NADP^+$ (2 μM), $MgCl_2$ (25 mM), $PolyP_6$ (8 mM), β-D-glucose (100 mM), *Ni*CAR/BID-*Ni*CAR (0.2 U/mL), *Bp*GDH/BID-*Bp*GDH (0.2 U/mL), PPK12/BID-PPK12 (5 U/mL), *Ec*PPase/BID-*Ec*PPase (5 U/mL), 2 μM BID protein, MOPS Buffer (100 mM, pH 7.5), at 30°C. (B) Reaction conditions: substrate 1a (15 mM), ATP (0.1 mM), $NADP^+$ (2 μM), $MgCl_2$ (25 mM), $PolyP_6$ (8 mM), β-D-glucose (100 mM), *Ni*CAR/BID-*Ni*CAR (0.2 U/mL), *Bp*GDH/BID-*Bp*GDH (0.2 U/mL), PPK12/BID-PPK12 (5 U/mL), *Ec*PPase/BID-*Ec*PPase (5 U/mL), 2 μM BID protein, MOPS Buffer (100 mM, pH 7.5), at 30°C. Data in panel (A) and (B) are presented as mean ± SD from three independent experiments (n=3). Statistical significance was determined by two-way ANOVA followed by Sidak's multiple comparisons test. Asterisks indicate significant differences compared to the free enzyme system at the corresponding time point (*$p < 0.05$; ** $p < 0.01$; *** $p < 0.001$; **** $p < 0.0001$).

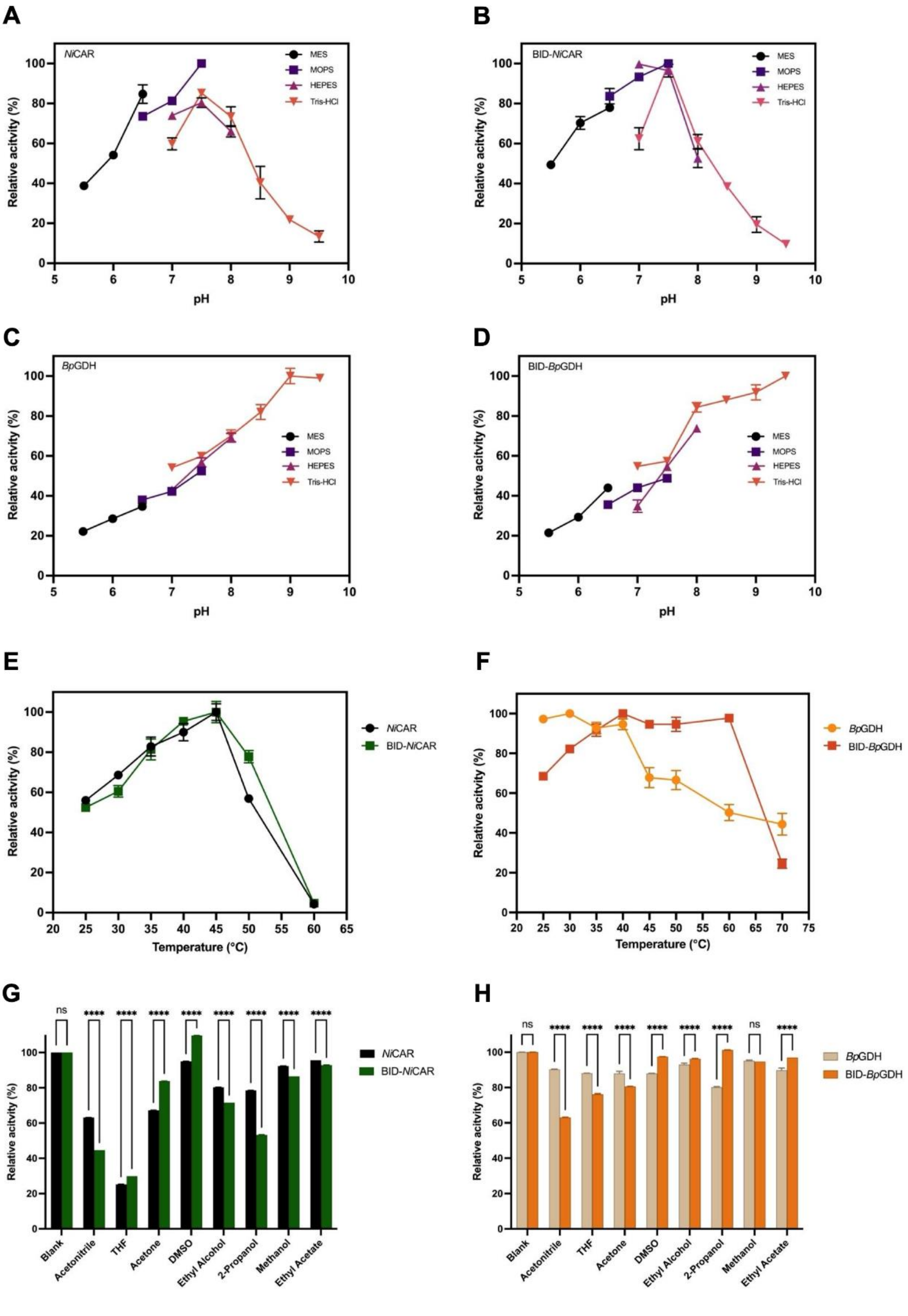

Figure S10. Characterization of CARs and GDHs. (A) Optimal pH of *Ni*CAR. Buffers with various pH were used: MES pH 5.5-6.5, MOPS pH 6.5-7.5, HEPES pH 7.0-8.0, Tris-HCl pH 7.0-9.5. (B) Optimal pH of BID-*Ni*CAR. Buffers with various pH were used as mentioned before. (C) Optimal pH of *Bp*GDH. Buffers with various pH were used as mentioned before. (D) Optimal pH of BID-

*Bp*GDH. Buffers with various pH were used as mentioned before. (E) Optimum reaction temperature of *Ni*CAR and BID-*Ni*CAR. (F) Optimum reaction temperature of *Bp*GDH and BID-*Bp*GDH. (G) Tolerance of *Ni*CAR and BID-*Ni*CAR to common organic solvents. (H) Tolerance of *Bp*GDH and BID-*Bp*GDH to common organic solvents.

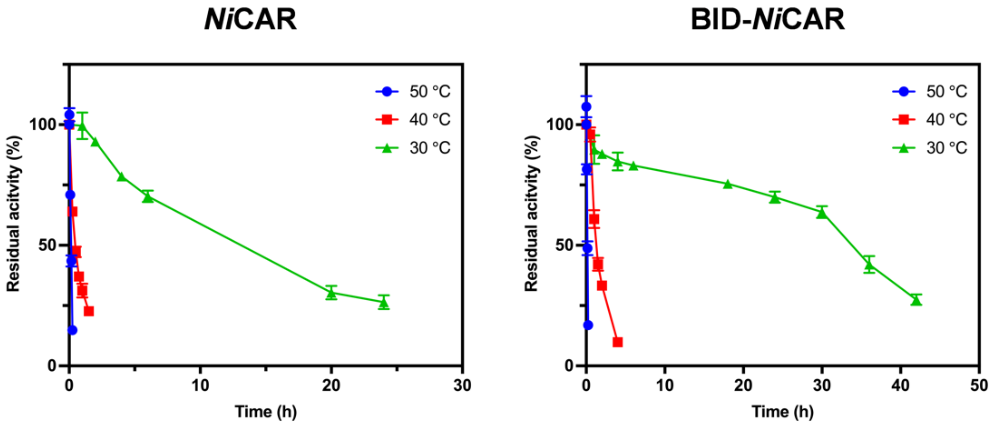

Enzyme	30 ℃		40 ℃		50 ℃	
	k_d/h^{-1}	$t_{1/2}$/h	k_d/h^{-1}	$t_{1/2}$/h	k_d/min^{-1}	$t_{1/2}$/min
*Ni*CAR	0.058	11.95	0.9576	0.72	0.1243	5.58
BID-*Ni*CAR	0.0231	30	0.6053	1.14	0.1164	5.95

Figure S11. Thermostability of *Ni*CAR and BID-*Ni*CAR at 30°C, 40°C, 50°C.

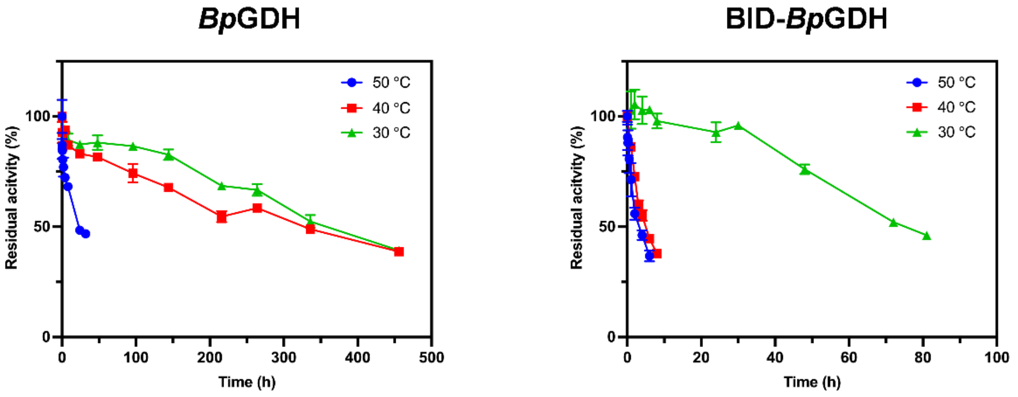

Enzyme	30°C		40°C		50°C	
	k_d/h^{-1}	$t_{1/2}$/h	k_d/h^{-1}	$t_{1/2}$/h	k_d/h^{-1}	$t_{1/2}$/h
*Bp*GDH	0.0018	385	0.0019	364.74	0.0208	33.32
BID-*Bp*GDH	0.0095	72.95	0.1219	5.68	0.1604	4.32

Figure S12. Thermostability of *Bp*GDH and BID-*Bp*GDH at 30°C, 40°C and 50°C.

Table S3. Substrate scope and catalytic activity of *Ni*CAR and BID-*Ni*CAR.

		**1a**	**1b**	**1c**	**1d**	**1e**
*Ni*CAR	K_m (mM)	0.45±0.03	1.04±0.09	4.49±0.62	1.58±0.14	1.85±0.16
	k_{cat} (min^{-1})	235.36±2.27	189.00±3.53	95.04±5.25	113.62±2.32	189.98±4.75
	k_{cat}/K_m (min^{-1} mM^{-1})	520.72±38.96	181.52±15.38	21.18±3.14	72.09±6.55	102.43±9.39
BID-*Ni*CAR	K_m (mM)	1.06±0.03	2.02±0.22	6.27±0.70	1.03±0.06	1.41±0.11
	k_{cat} (min^{-1})	243.37±2.36	138.16±4.26	106.98±5.52	68.23±0.99	174.17±2.56
	k_{cat}/K_m (min^{-1} mM^{-1})	229.58±7.48	68.25±7.65	17.05±2.09	65.93±4.09	123.01±9.62

		**1f**	**1g**	**1h**	**1i**
*Ni*CAR	K_m (mM)	101.93±21.09	1.06±0.09	0.75±0.05	1.42±0.05
	k_{cat} (min^{-1})	333.83±63.15	191.30±2.15	183.86±2.22	86.02±1.35
	k_{cat}/K_m (min^{-1} mM^{-1})	3.27±0.92	180.08±14.99	243.67±17.27	60.75±2.48
BID-*Ni*CAR	K_m (mM)	81.19±22.07	1.87±0.16	0.75±0.05	1.21±0.08
	k_{cat} (min^{-1})	215.71±52.36	165.10±3.22	197.78±2.38	94.33±075
	k_{cat}/K_m (min^{-1} mM^{-1})	2.66±0.97	88.36±7.91	263.65±16.95	77.96±4.88

Reaction conditions: Carboxylic acid **1a-1i** (10 mM), $MgCl_2$ (10 mM), DTT (1 mM), ATP (1 mM), NADPH (0.2 mM), 0.5 μM enzyme. Reaction was performed in 100 mM MOPS, pH 7.5 at 30°C.

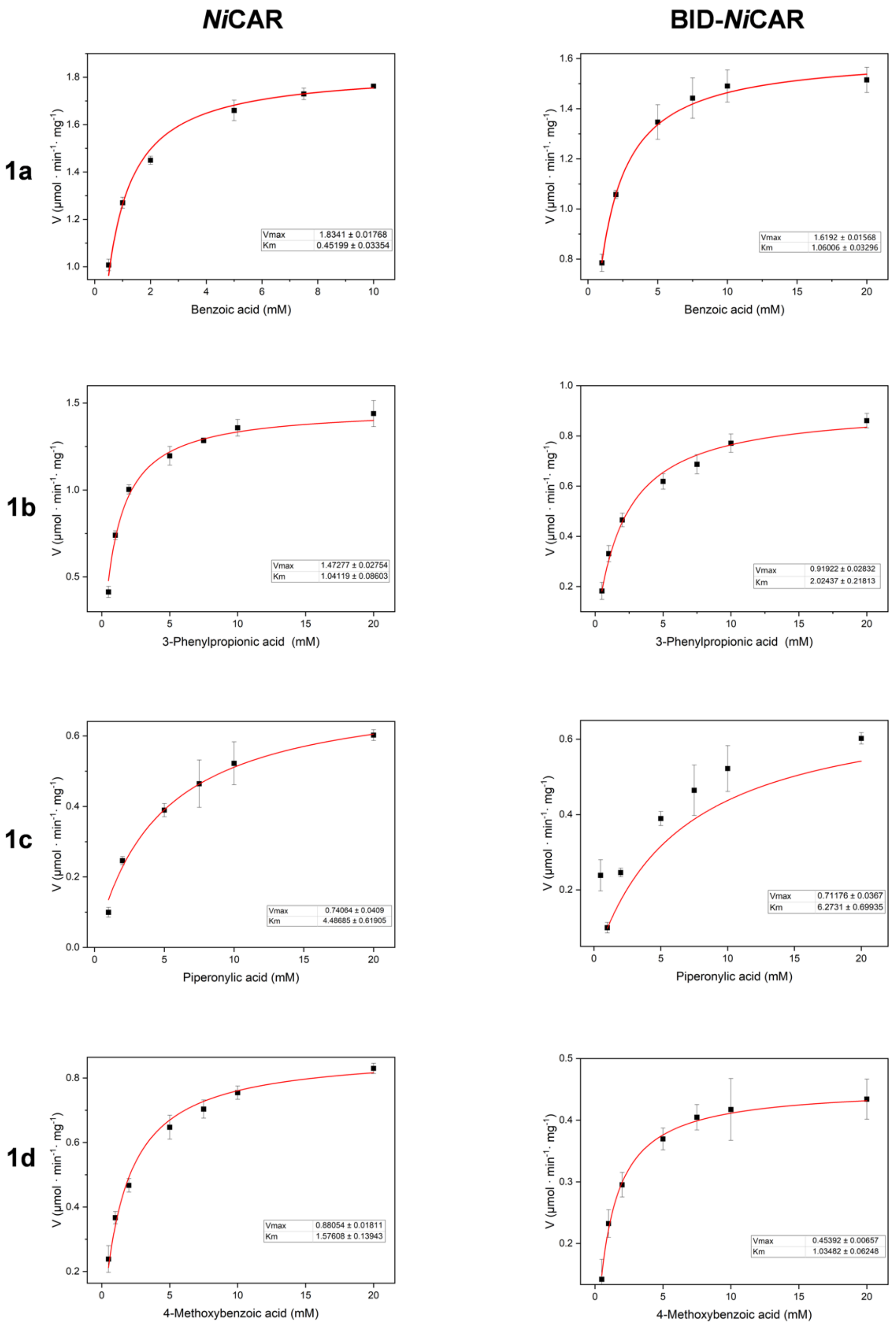
NiCAR
BID-NiCAR
1a
1b
1c
1d
V (µmol · min-1 · mg-1)
Benzoic acid (mM)
Vmax 1.8341 ± 0.01768
Km 0.45199 ± 0.03354
Vmax 1.6192 ± 0.01568
Km 1.06006 ± 0.03296
3-Phenylpropionic acid (mM)
Vmax 1.47277 ± 0.02754
Km 1.04119 ± 0.08603
Vmax 0.91922 ± 0.02832
Km 2.02437 ± 0.21813
Piperonylic acid (mM)
Vmax 0.74064 ± 0.0409
Km 4.48685 ± 0.61905
Vmax 0.71176 ± 0.0367
Km 6.2731 ± 0.69935
4-Methoxybenzoic acid (mM)
Vmax 0.88054 ± 0.01811
Km 1.57608 ± 0.13943
Vmax 0.45392 ± 0.00657
Km 1.03482 ± 0.06248

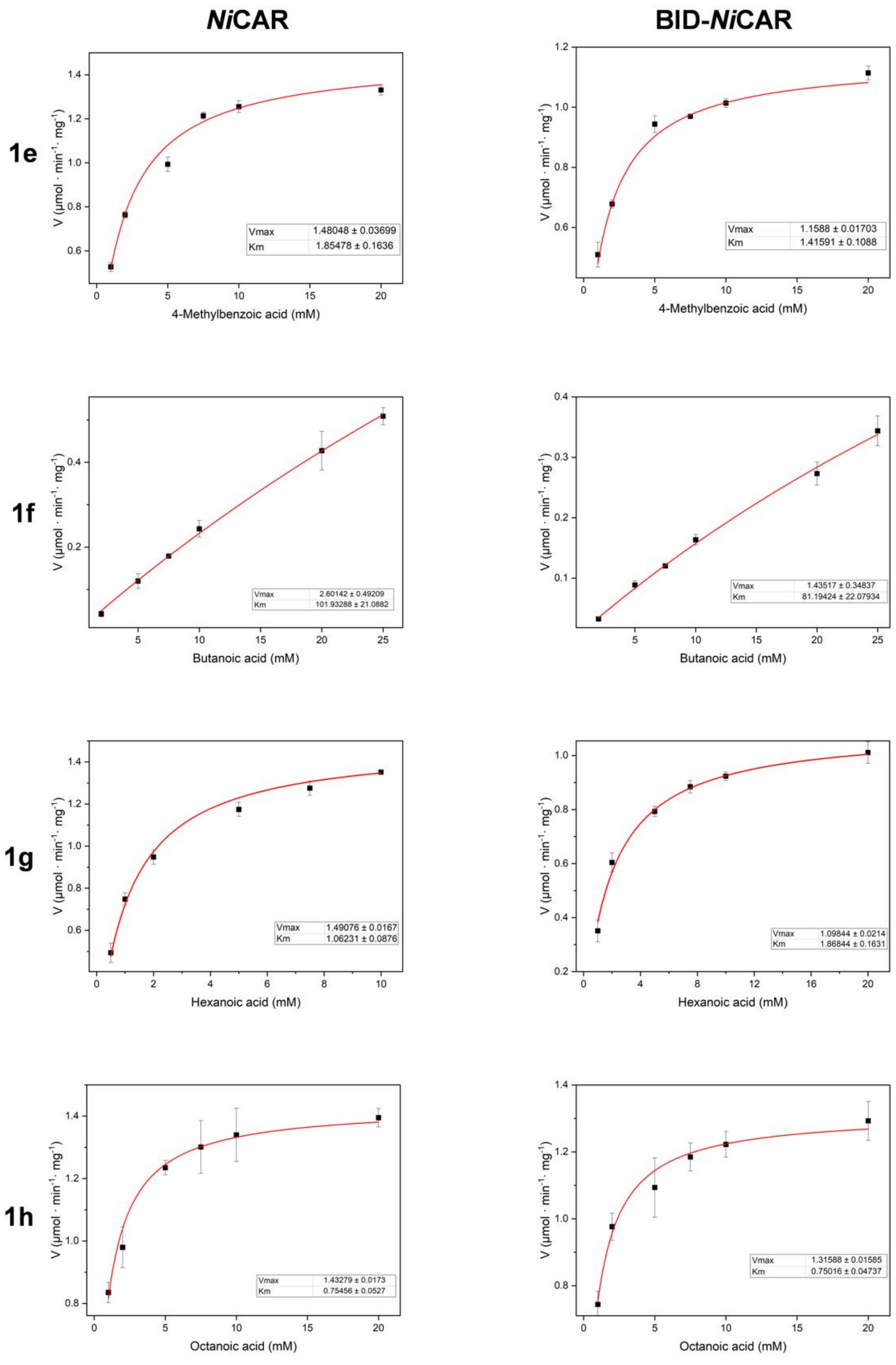

*Ni*CAR
BID-*Ni*CAR
1e
Vmax 1.48048 ± 0.03699
Km 1.85478 ± 0.1636
4-Methylbenzoic acid (mM)
Vmax 1.1588 ± 0.01703
Km 1.41591 ± 0.1088
4-Methylbenzoic acid (mM)
1f
Vmax 2.60142 ± 0.49209
Km 101.93288 ± 21.0882
Butanoic acid (mM)
Vmax 1.43517 ± 0.34837
Km 81.19424 ± 22.07934
Butanoic acid (mM)
1g
Vmax 1.49076 ± 0.0167
Km 1.06231 ± 0.0876
Hexanoic acid (mM)
Vmax 1.09844 ± 0.0214
Km 1.86844 ± 0.1631
Hexanoic acid (mM)
1h
Vmax 1.43279 ± 0.0173
Km 0.75456 ± 0.0527
Octanoic acid (mM)
Vmax 1.31588 ± 0.01585
Km 0.75016 ± 0.04737
Octanoic acid (mM)
V (µmol · min-1 · mg-1)

1i

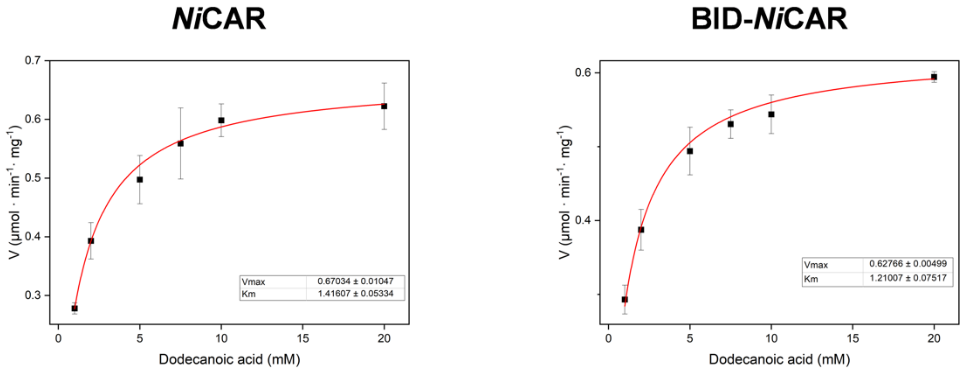

Figure S13. The kinetic constants of *Ni*CAR and BID-*Ni*CAR toward different acid substrates. The enzyme activities were assayed in 100mM MOPS pH 7.5, 10 mM $MgCl_2$, 1 mM DTT, 1 mM ATP, 0.2 mM NADPH, 0.5-20 mM substrate, 0.5 μM enzyme, at 30°C.

4. HPLC and GC chromatograms

(1) HPLC

Benzoic acid **1a** 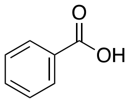

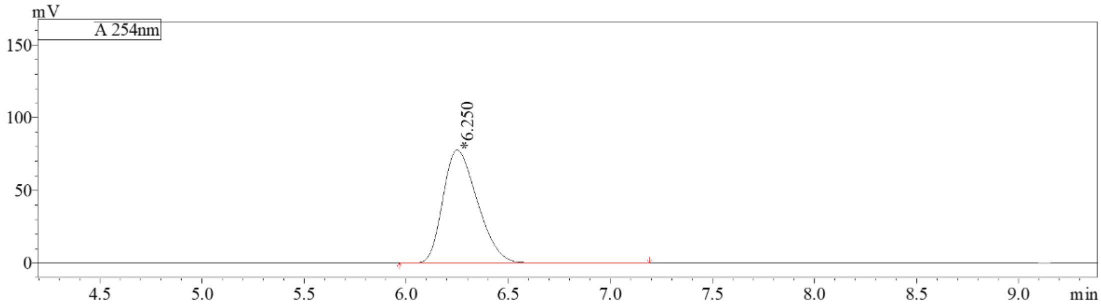

Benzaldehyde **2a** 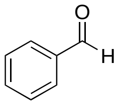

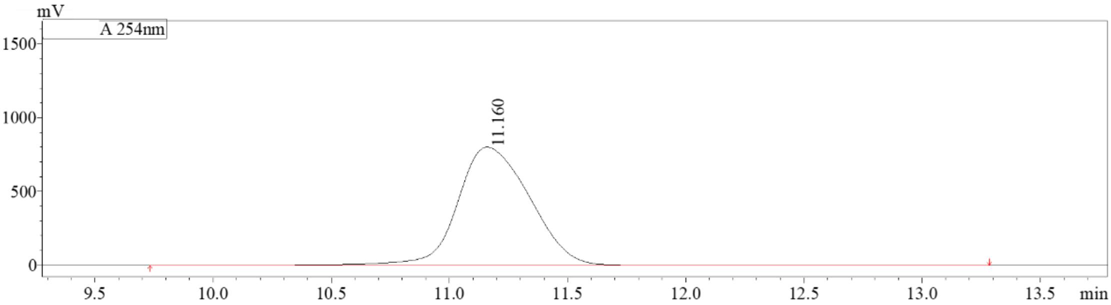

(2) GC-FID

Hexanoic acid **1g** 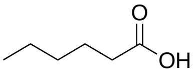

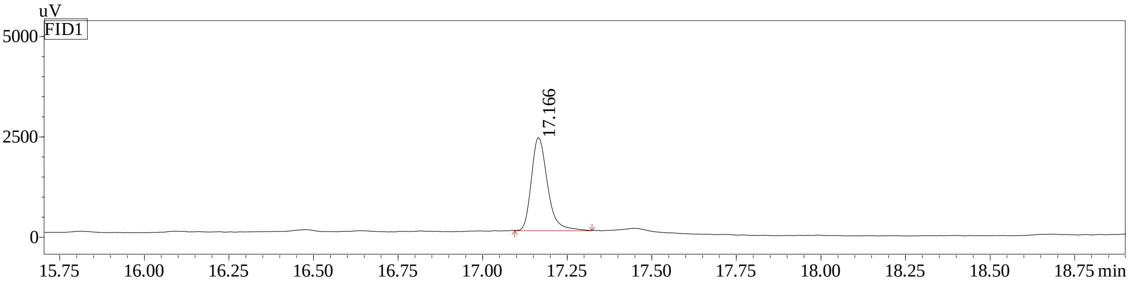

Hexanal **2g** 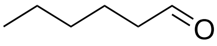

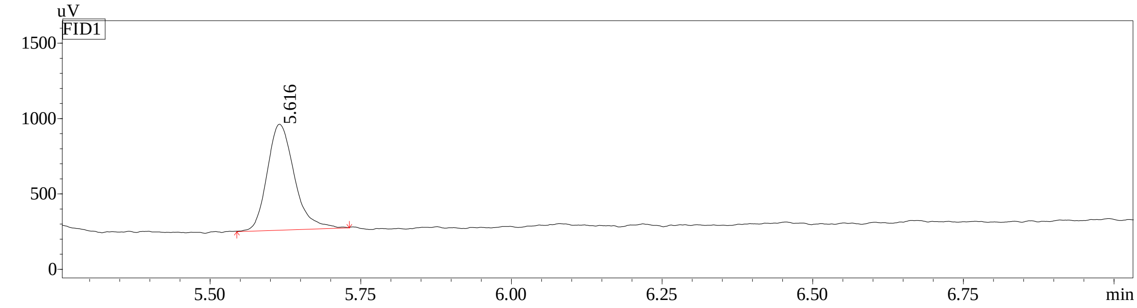

N-propargylhexylamine 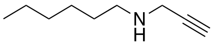

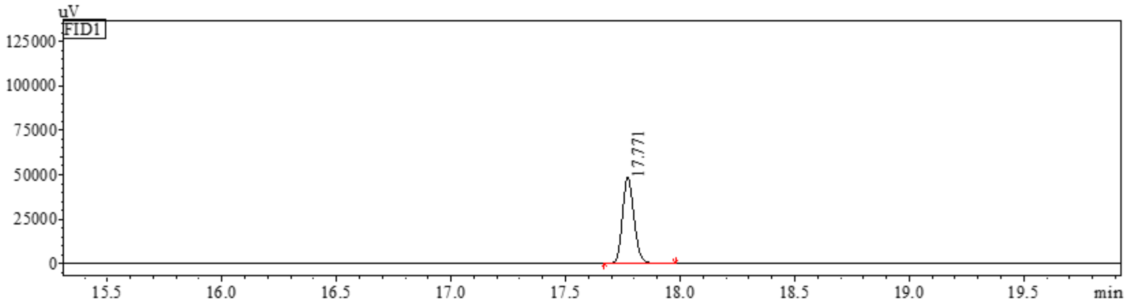

N-cyclopropylhexylamine 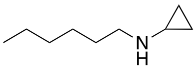

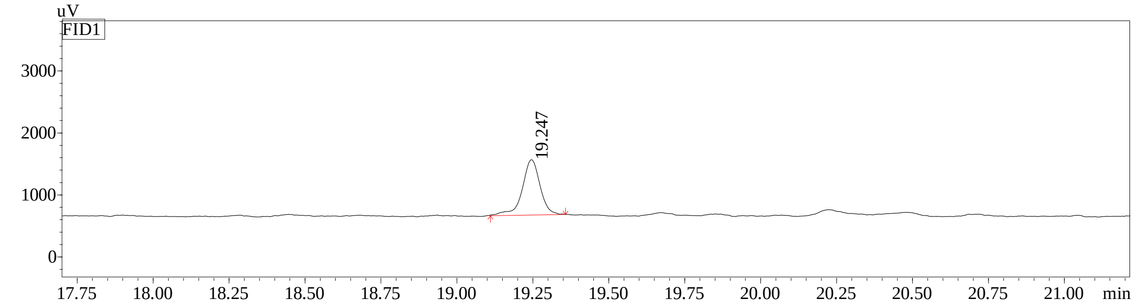

5. Calibration curves and representative HPLC traces

Calibration Curves

(1) Benzoic acid **1a**

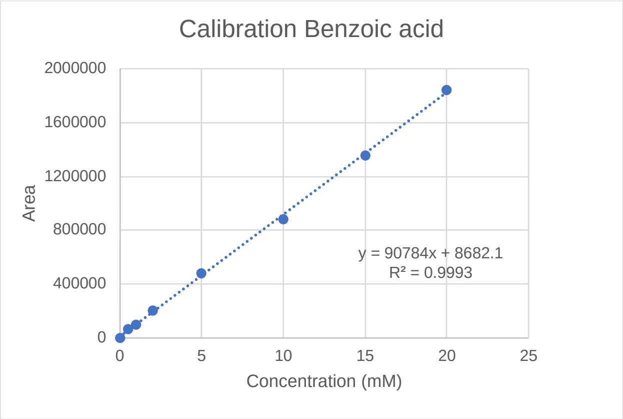

(2) Benzaldehyde **2a**

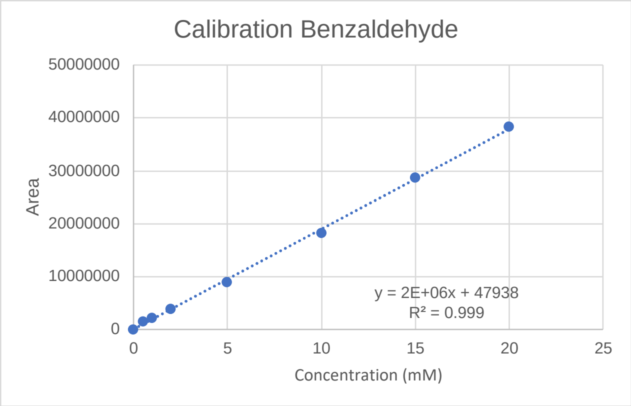

(3) Hexanoic acid **1g**

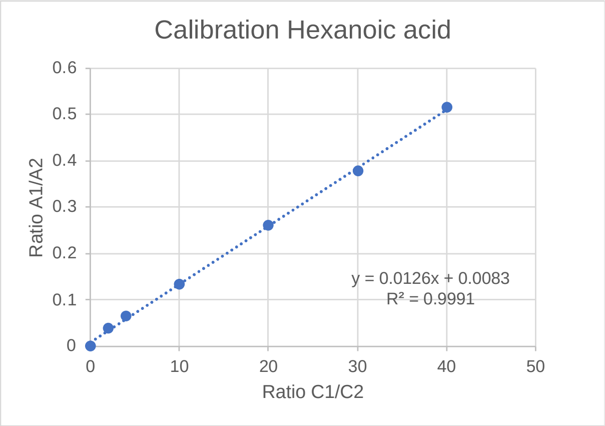

(4) N-propargylhexylamine

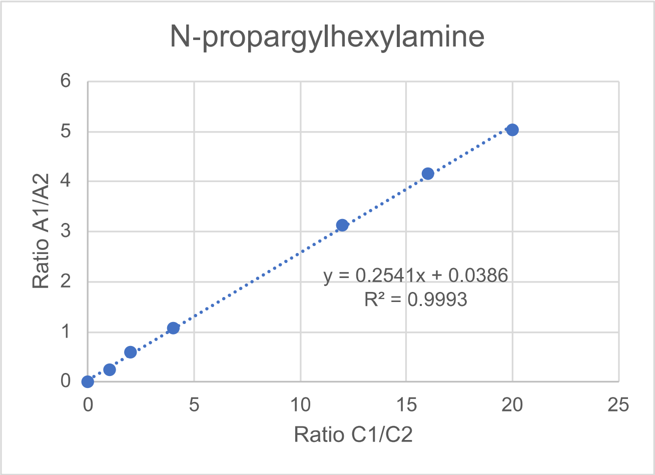

(5) N-cyclopropylhexylamine

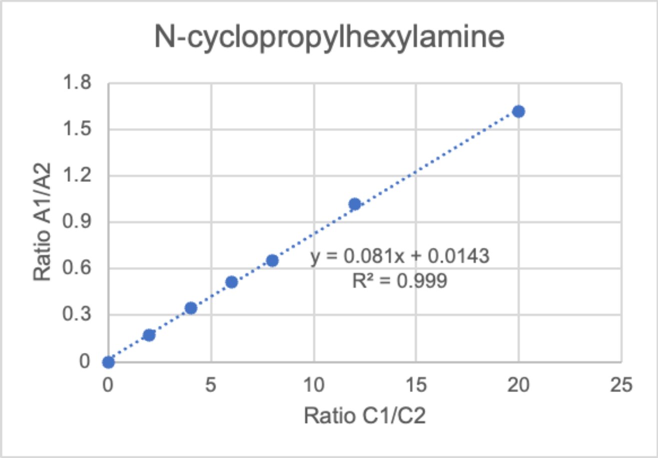

HPLC traces

(1) Tetra-enzyme condensates, 0.5 h

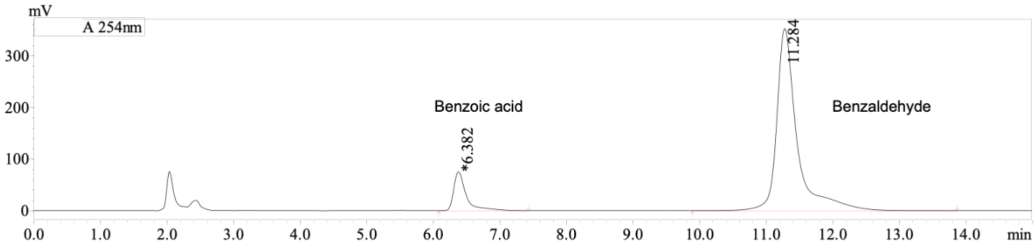

(2) Tetra-enzyme condensates, 4 h

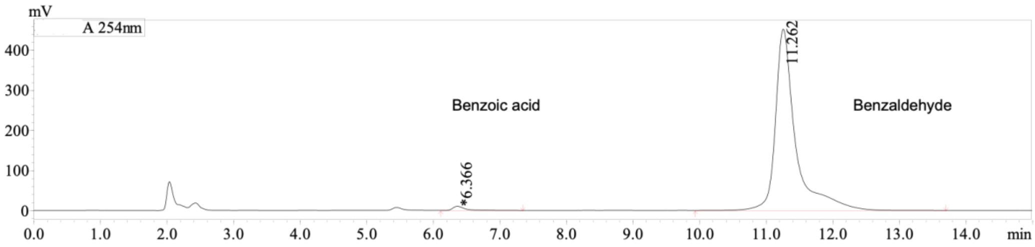

6. ^{1}H-NMR and ^{13}C-NMR

N-propargylhexylamine

^{1}H NMR (400 MHz, $CDCl_3$) δ 3.46 (d, J = 2.5 Hz, 2H), 2.58 – 2.50 (m, 1H), 2.23 (t, J = 2.4 Hz, 1H), 1.53 – 1.42 (m, 1H), 1.39 – 1.29 (m, 3H), 1.29 – 1.22 (m, 2H), 0.92 – 0.84 (m, 2H).

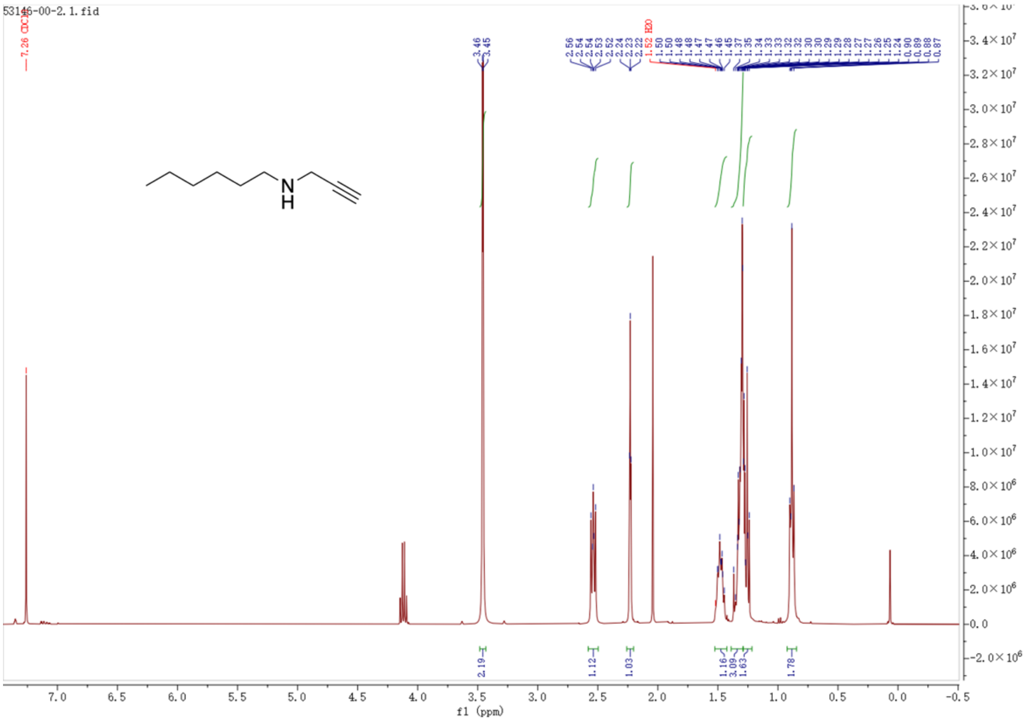

^{13}C NMR (101 MHz, $CDCl_3$) δ 78.42, 72.98, 60.27, 52.92, 41.94, 31.56, 29.57, 27.18, 26.87, 22.47, 13.92.

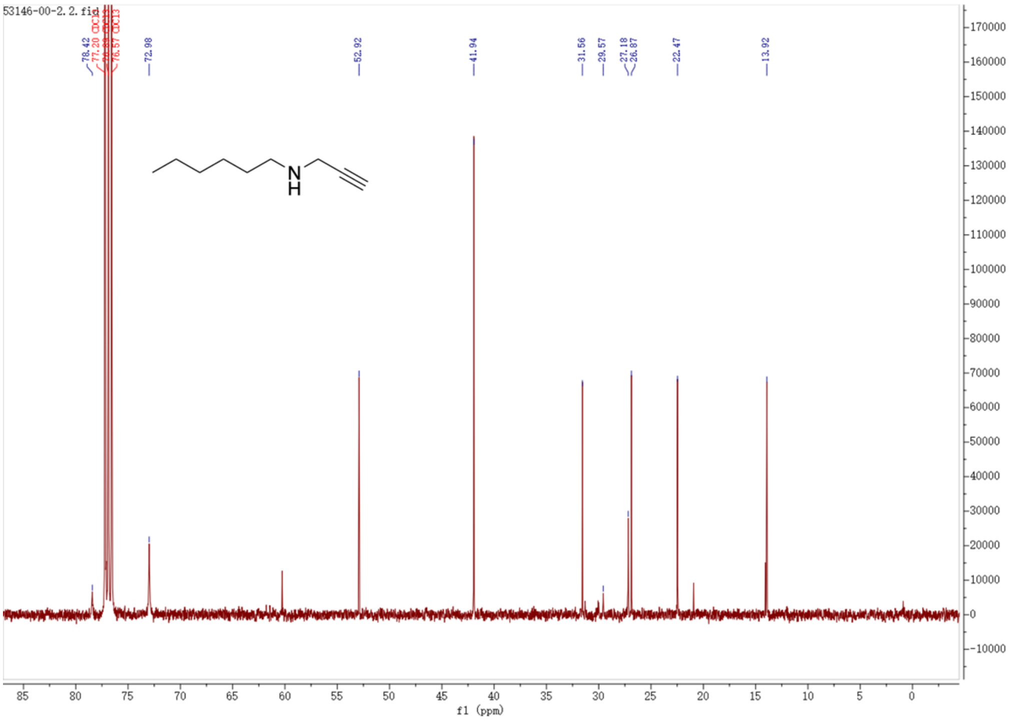

N-cyclopropylhexylamine

^{1}H NMR (400 MHz, $CDCl_3$) δ 4.88 – 4.83 (m, 2H), 4.17 – 4.00 (m, 13H), 3.60 (ddt, J = 34.8, 11.5, 5.5 Hz, 3H), 2.55 (s, 4H), 1.98 (s, 16H), 1.19 (t, J = 7.1 Hz, 19H), 0.80 (s, 1H).

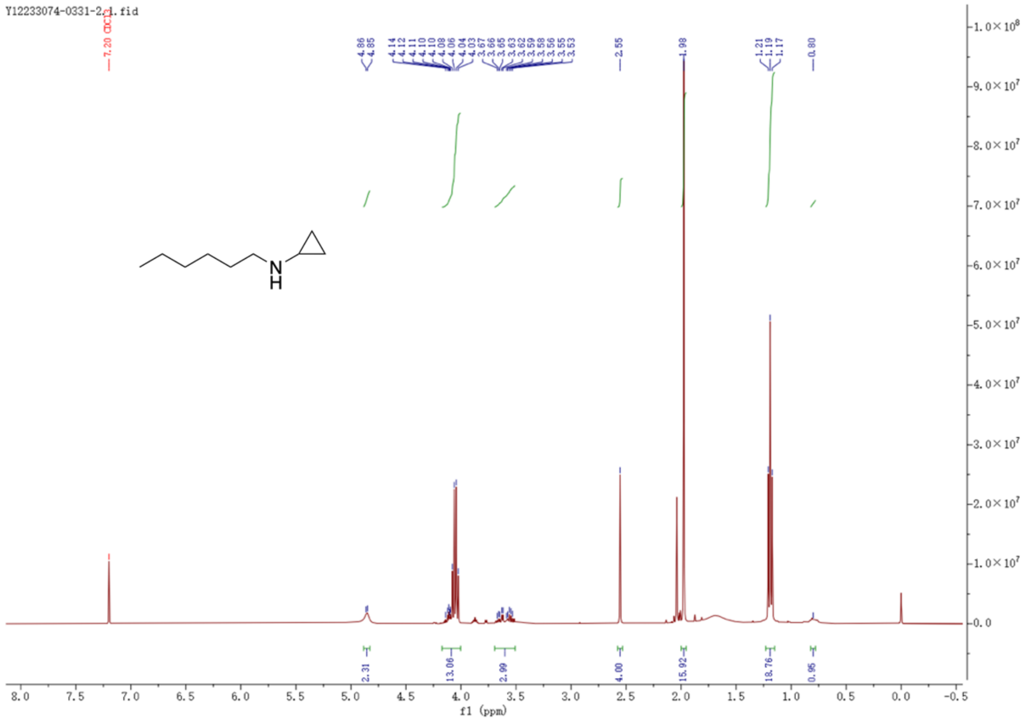

^{13}C NMR (101 MHz, $CDCl_3$) δ 70.19, 65.41, 65.24, 63.35, 60.42, 40.91, 21.06, 20.85, 20.80, 14.20.

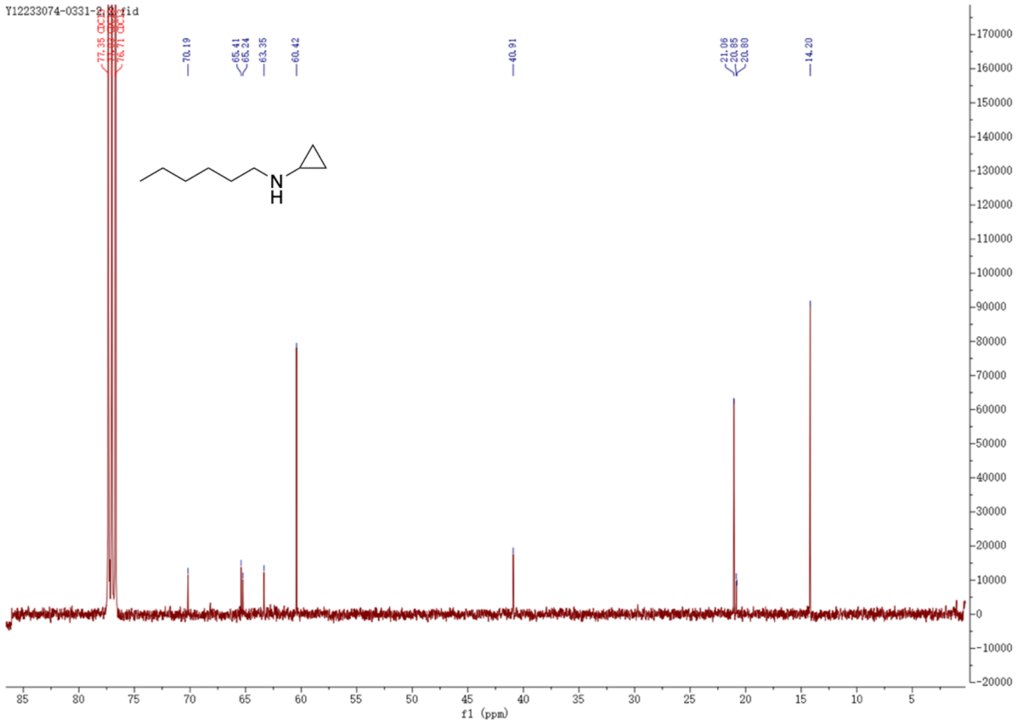

7. References

1. Nocek, B.; Kochinyan, S.; Proudfoot, M.; Brown, G.; Evdokimova, E.; Osipiuk, J.; Edwards, A.M.; Savchenko, A.; Joachimiak, A.; Yakunin, A.F. Polyphosphate-Dependent Synthesis of ATP and ADP by the Family-2 Polyphosphate Kinases in Bacteria. *Proc. Natl. Acad. Sci.* **2008**, *105*, 17730–17735, doi:10.1073/pnas.0807563105.
